# Supplementary material for: Combinatorial analysis reveals highly coordinated early-stage immune reactions that predict later antiviral immunity in mild COVID-19 patients
Source: Cell Rep Med. 2022 Mar 29;3(4):100600. doi: 10.1016/j.xcrm.2022.100600 (PMC8960124; doi:10.1016/j.xcrm.2022.100600)
Supplement: Document S1. Figures S1–S8 and Table S1 [file mmc1.pdf]

**Cell Reports Medicine, Volume *3***

## Supplemental information

# Combinatorial analysis reveals highly coordinated early-stage immune reactions that predict later antiviral immunity in mild COVID-19 patients

**Christophe M. Capelle, Séverine Ciré, Olivia Domingues, Isabelle Ernens, Fanny Hedin, Aurélie Fischer, Chantal J. Snoeck, Wim Ammerlaan, Maria Konstantinou, Kamil Grzyb, Alexander Skupin, Cara L. Carty, Christiane Hilger, Georges Gilson, Aljosa Celebic, Paul Wilmes, Antonio Del Sol, Ian M. Kaplan, Fay Betsou, Tamir Abdelrahman, Antonio Cosma, Michel Vaillant, Guy Fagherazzi, Markus Ollert, and Feng Q. Hefeng**

# Supplemental Information

## This file includes:

**Figure S1-S8**

**Table S1**

## Supplementary Figures:

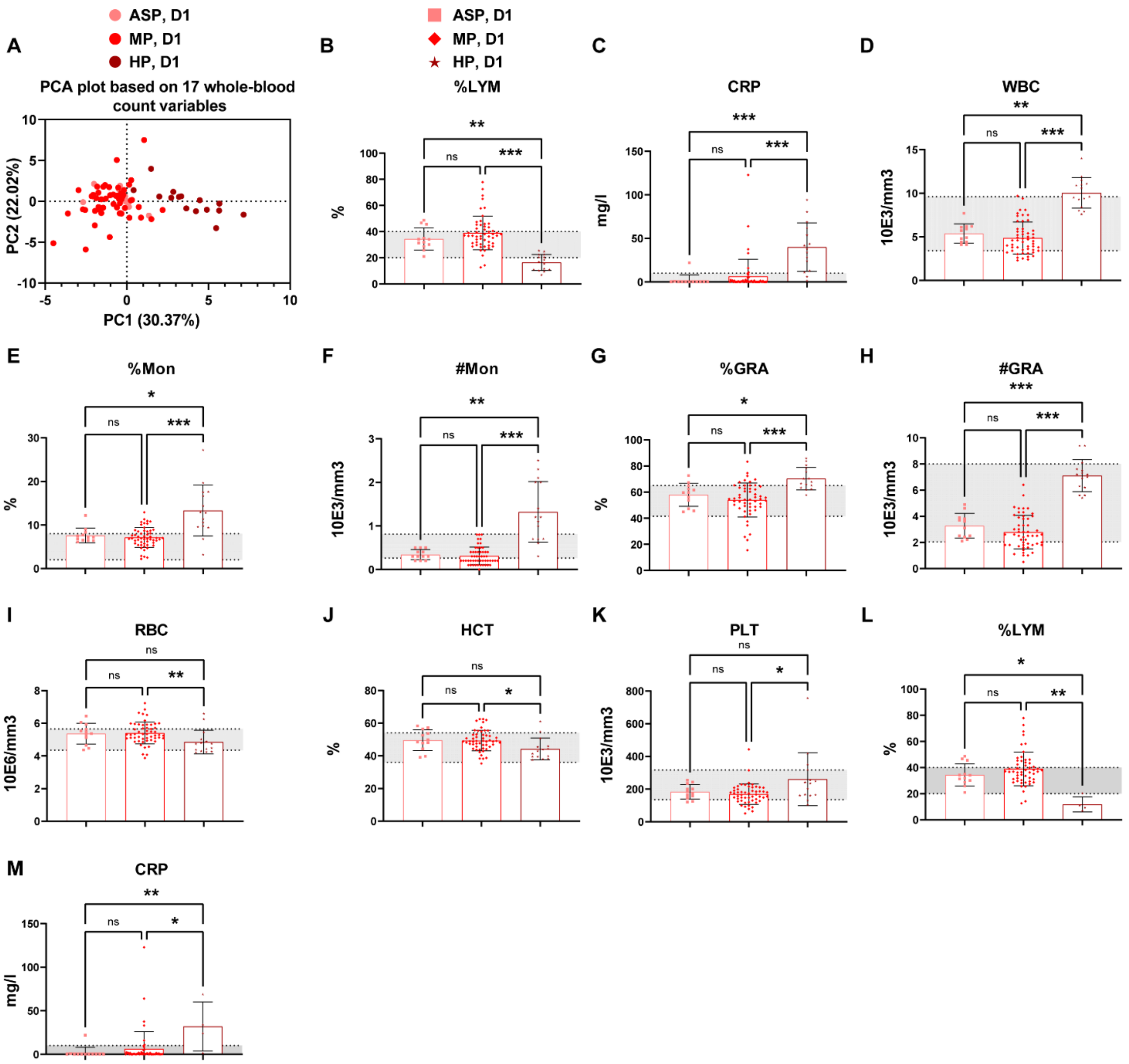


**Figure S1. Whole-blood-count analysis of asymptomatic, mild and hospitalized patients at day 1 of inclusion.**

**A**, PCA plot of the samples from different patient groups at D1 of inclusion based on 17-parameter whole-blood-count analysis. The percentage of explained variance is shown for each axis. ASP, asymptomatic patients, n=12; HP, hospitalized patients, n=15; MP, mild patients, n=53; D1, day 1.
**B, E, G**, the percentages of Lymphocytes (LYM, **B**), monocytes (Mon, **E**) and granulocytes (GRA, **G**) among white blood cells.
**C**, CRP (C-reactive protein) levels in different patient groups at D1 of inclusion.
**D, F, H, I, K**, Number of white blood cells (WBC, **D**), monocytes (Mon, **F**), granulocytes (GRA, **H**), red blood cells (RBC, **I**) and platelets (PLT, **K**) per µl (mm$^3$).
**J**, the hematocrit (HCT) levels (%) from different patients groups at D1 of inclusion.
**L, M**, Percentages of LYM in white blood cells (**L**) or CRP levels (**M**) from all ASP, all MP and four selected HP. To ensure the comparability with MP, we only selected four HP with at most 7-day delay from the onset of first symptoms to the inclusion day in these two panels. The data in **L** and **M** are identical to that in **B** and **C** except for HP.
Data represent individual values from all biological replicates; Mean ± standard deviation (S.D.); P-value was determined by the Kruskal-Wallis (non-parametric) test and corrected using the Dunn's multiple comparisons test. ns, not significant, *p<=0.05, **p<=0.01 and ***p<=0.001. Gray shading indicates the reported normal range for those different laboratory parameters. Related to Figure 1.

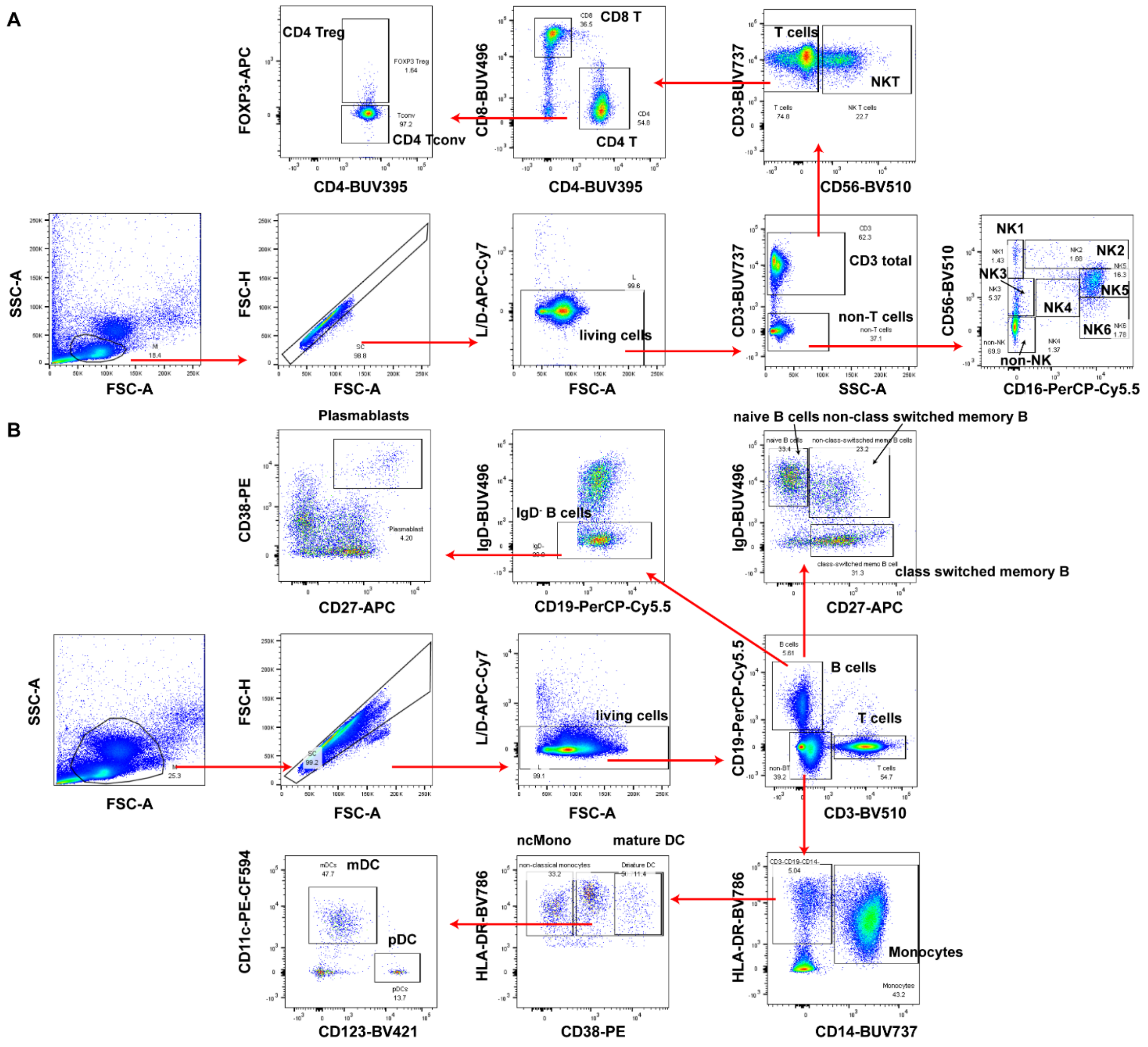


**Figure S2. General gating strategy to identify different immune subsets analysed in our study.**

**A**, Lymphocyte gating strategy (not including B cells). Treg, FOXP3$^{+}$CD4 regulatory T cells; Tconv, FOXP3$^{-}$CD4 conventional T cells; NK, natural killer cells; NKT, natural killer T cells.

**B,** Gating strategy to identify B cells, (non-) class-switched memory B cells, naïve B cells, T cells, subsets of monocytes, DC, mDC, pDC, mature DC and plasmablasts. ncMono, non-classical monocytes; DC, dendritic cells; mDC, myeloid DC; pDC, plasmacytoid DC.

Due to limited space, the functional markers or their combinations among each subset were not displayed here. It is also worthy to note that we used three different panels to perform a deep immunophenotyping analysis by flow cytometry. For some markers, different fluorochromes were used in different panels. Related to Figures 2 and 3.

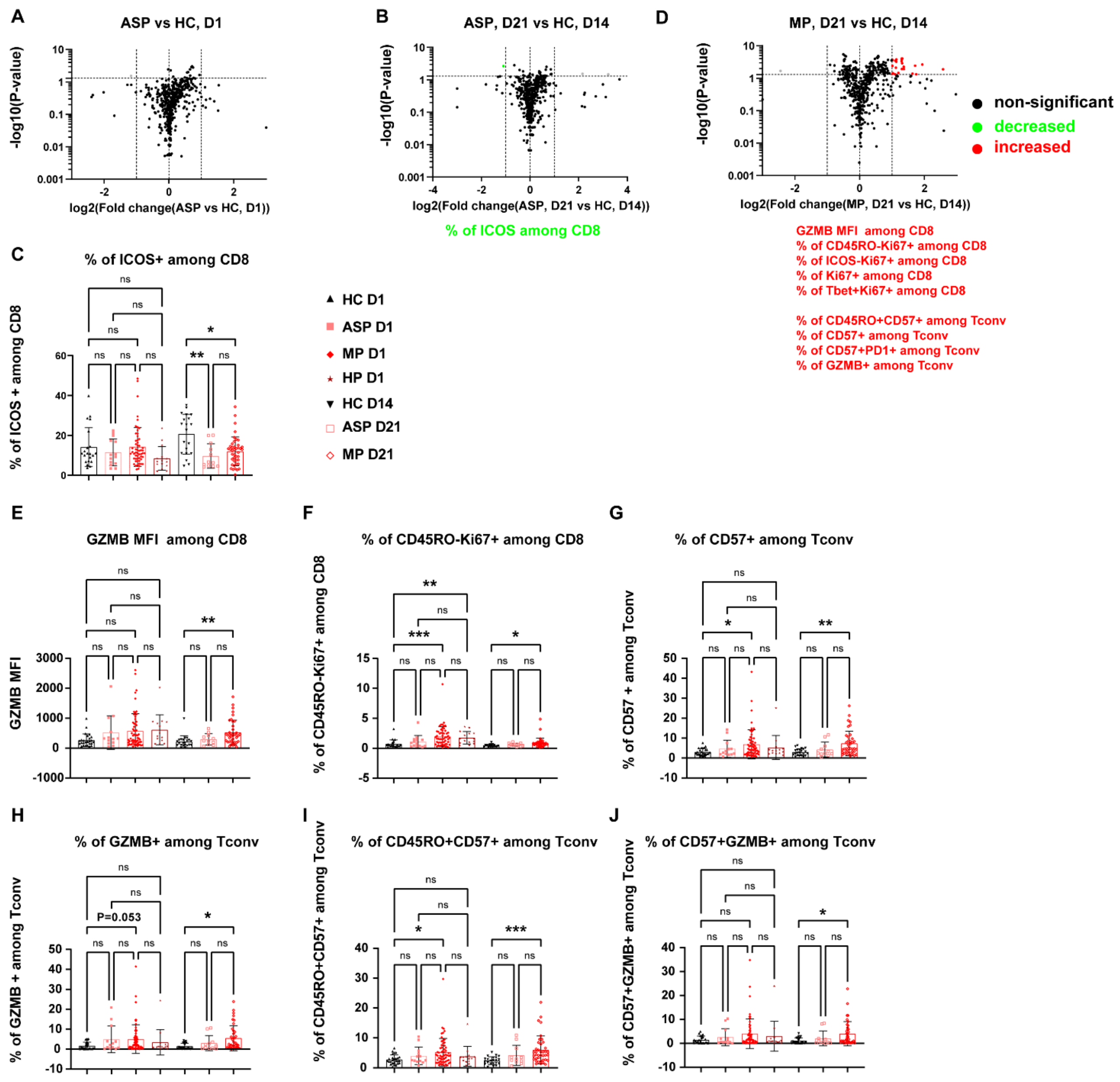


**Figure S3. Extended comparisons of immune subset responses between various groups.**

**A**, **B**, Volcano plots showing the immune responses in ASP vs HC at D1 (baseline) (**A**) or ASP at D21 vs HC at D14 (**B**) following inclusion. Significant increased or decreased subsets (p<=0.05 and change fold >=2) were marked in red or green, respectively. The gray dot represents the subset not showing a significant change after applying the Dunn's multiple comparison test.

**C**, Frequency of ICOS$^{+}$ cells among CD8 T cells of different participant groups.

**D**, Volcano plot showing responses of different immune subsets in MP at D21 following inclusion relative to HC at D14 following inclusion. Significantly increased or decreased subsets (p<=0.05 and change fold >=2) were marked in red or green, respectively. The gray dot represents the subset not showing a significant change after applying the Dunn's multiple comparison test.

**E**, GZMB MFI (Geometric mean) among CD8 T cells from different groups.

**F**, Frequency of CD45RO$^{-}$Ki67$^{+}$ cells among CD8 T cells from different groups.

**G, H, I, J**, Frequency of CD57$^{+}$ cells (**G**), GZMB$^{+}$ cells (**H**), CD45RO$^{+}$CD57$^{+}$ cells (**I**) or CD57$^{+}$GZMB$^{+}$ cells (**J**) among CD4 Tconv cells (FOXP3$^{-}$CD4 T conventional cells) from different groups.

Data represent individual values from all biological replicates; Mean± standard deviation (S.D.); P-value was determined by the Kruskal-Wallis (nonparametric) test and corrected using the Dunn's multiple comparisons test. ASP,

asymptomatic patients, n=14; HC, household controls, n=26; HP, hospitalized patients, n=15; MP, mild patients, n=63; D1/D14/D21, day 1/day 14/day 21. ns, not significant, *p<=0.05, **p<=0.01 and ***p<=0.001. Related to Figure 2.

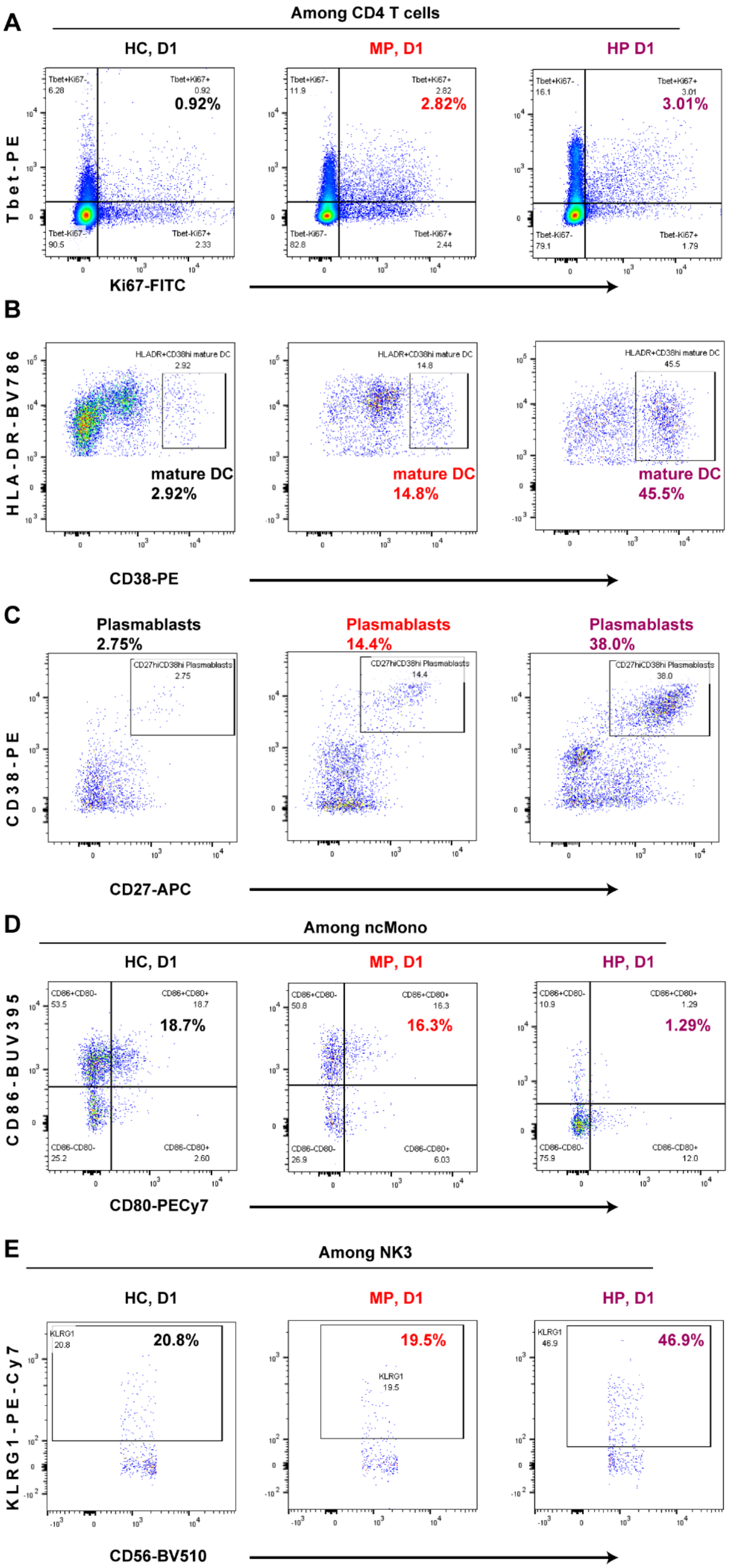
A
Among CD4 T cells
HC, D1
MP, D1
HP D1
Tbet-PE
Ki67-FITC
Tbet+Ki67-
6.28
Tbet+Ki67+
0.92
0.92%
Tbet-Ki67-
90.5
Tbet-Ki67+
2.33
Tbet+Ki67-
11.9
Tbet+Ki67+
2.82
2.82%
Tbet-Ki67-
82.8
Tbet-Ki67+
2.44
Tbet+Ki67-
16.1
Tbet+Ki67+
3.01
3.01%
Tbet-Ki67-
79.1
Tbet-Ki67+
1.79
B
HLA-DR-BV786
CD38-PE
HLADR+CD38hi mature DC
2.92
mature DC
2.92%
HLADR+CD38hi mature DC
14.8
mature DC
14.8%
HLADR+CD38hi mature DC
45.5
mature DC
45.5%
C
Plasmablasts
2.75%
Plasmablasts
14.4%
Plasmablasts
38.0%
CD38-PE
CD27-APC
CD27hiCD38hi Plasmablasts
2.75
CD27hiCD38hi Plasmablasts
14.4
CD27hiCD38hi Plasmablasts
38.0
D
Among ncMono
HC, D1
MP, D1
HP, D1
CD86-BUV395
CD80-PECy7
CD86+CD80-
53.5
CD86+CD80+
18.7
18.7%
CD86-CD80-
25.2
CD86-CD80+
2.60
CD86+CD80-
50.8
CD86+CD80+
16.3
16.3%
CD86-CD80-
26.9
CD86-CD80+
6.03
CD86+CD80-
10.9
CD86+CD80+
1.29
1.29%
CD86-CD80-
75.9
CD86-CD80+
12.0
E
Among NK3
HC, D1
MP, D1
HP, D1
KLRG1-PE-Cy7
CD56-BV510
KLRG1
20.8
20.8%
19.5%
KLRG1
19.5
KLRG1
46.9
46.9%

**Figure S4. Extended representative flow cytometry plots in comparison of different groups at day 1 of inclusion.**

**A**, Representative flow-cytometry plots of the expression of Ki67 and Tbet among CD4 T cells from different participant groups. HC, household controls; HP, hospitalized patients; MP, mild patients; D1, day 1.
**B, C**, Representative flow-cytometry plots of the expression of CD38 and HLA-DR showing mature DC (**B**) or the expression of CD38 and CD27 showing plasmablasts (**C**) from different participant groups.
**D**, Representative flow-cytometry plots of the expression CD86 and CD80 among non-classical monocytes (ncMono) from different groups.
**E**, Representative flow-cytometry plots of the expression KLRG1 and CD56 among NK3 from different groups.
Related to Figures 2 and 3.

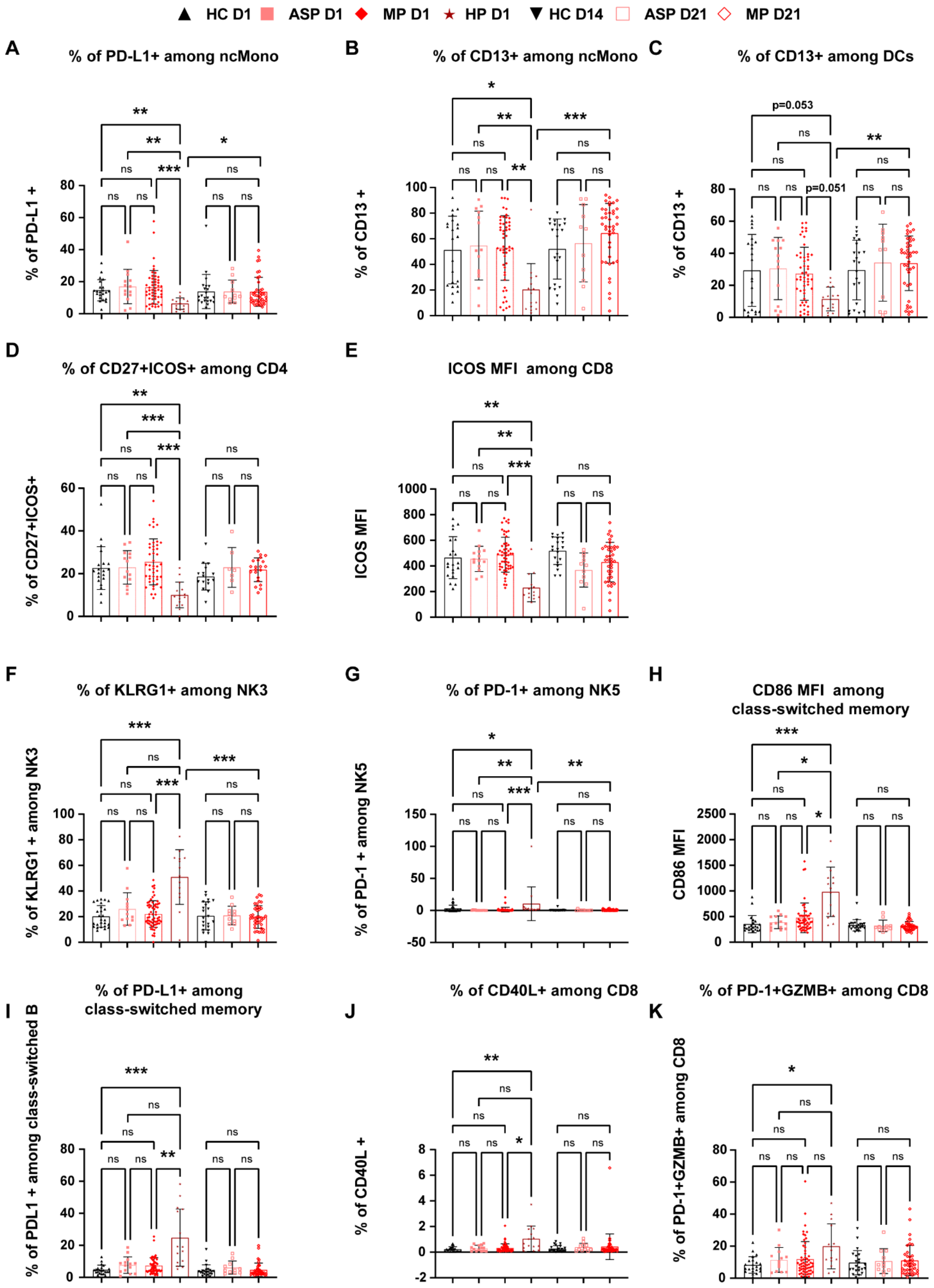

HC D1
ASP D1
MP D1
HP D1
HC D14
ASP D21
MP D21
A
% of PD-L1+ among ncMono
% of PD-L1 +
B
% of CD13+ among ncMono
% of CD13 +
C
% of CD13+ among DCs
% of CD13 +
p=0.053
p=0.051
D
% of CD27+ICOS+ among CD4
% of CD27+ICOS+
E
ICOS MFI among CD8
ICOS MFI
F
% of KLRG1+ among NK3
% of KLRG1 + among NK3
G
% of PD-1+ among NK5
% of PD-1 + among NK5
H
CD86 MFI among class-switched memory
CD86 MFI
I
% of PD-L1+ among class-switched memory
% of PDL1 + among class-switched B
J
% of CD40L+ among CD8
% of CD40L +
K
% of PD-1+GZMB+ among CD8
% of PD-1+GZMB+ among CD8

**Figure S5. Extended analysis of early-stage immune features characterizing mild patents from hospitalized ones.**

**A**, **B**, Frequency of cells expressing PD-L1 (**A**) or CD13 (**B**) among ncMono (non-classical monocytes) from different participant groups.
**C**, Frequency of $CD13^{+}$ cells among HLA-$DR^{+}CD38^{+}$ DCs (including $CD38^{high}$ cells, refer to Fig. S2).
**D, E,** Frequency of $CD27^{+}ICOS^{+}$ cells among CD4 cells (**D**) or ICOS MFI (Geometric mean) (**E**) among CD8 T cells.
**F, G**, Frequency of $KLRG1^{+}$ cells among NK3 (**F**) or frequency of PD-$1^{+}$ cells among NK5 (**G**).
**H, I**, CD86 MFI (**H**) or the frequency of PD-$L1^{+}$ cells (**I**) among class-switched memory B cells.
**J, K**, Frequency of $CD40L^{+}$ cells (**J**) or frequency of PD-$1^{+}GZMB^{+}$ cells (**K**) among CD8 T cells.
Data represent individual values from all biological replicates; Mean± standard deviation (S.D.); P-value was determined by the Kruskal-Wallis (nonparametric) test and corrected using the Dunn's multiple comparisons test. ASP, asymptomatic patients, n=14; HC, household controls, n=26; HP, hospitalized patients, n=15; MP, mild patients, n=63; D1/D14/D21, day 1/day 14/day 21. ns, not significant, *p<=0.05, **p<=0.01 and ***p<=0.001. Related to Figure 3.

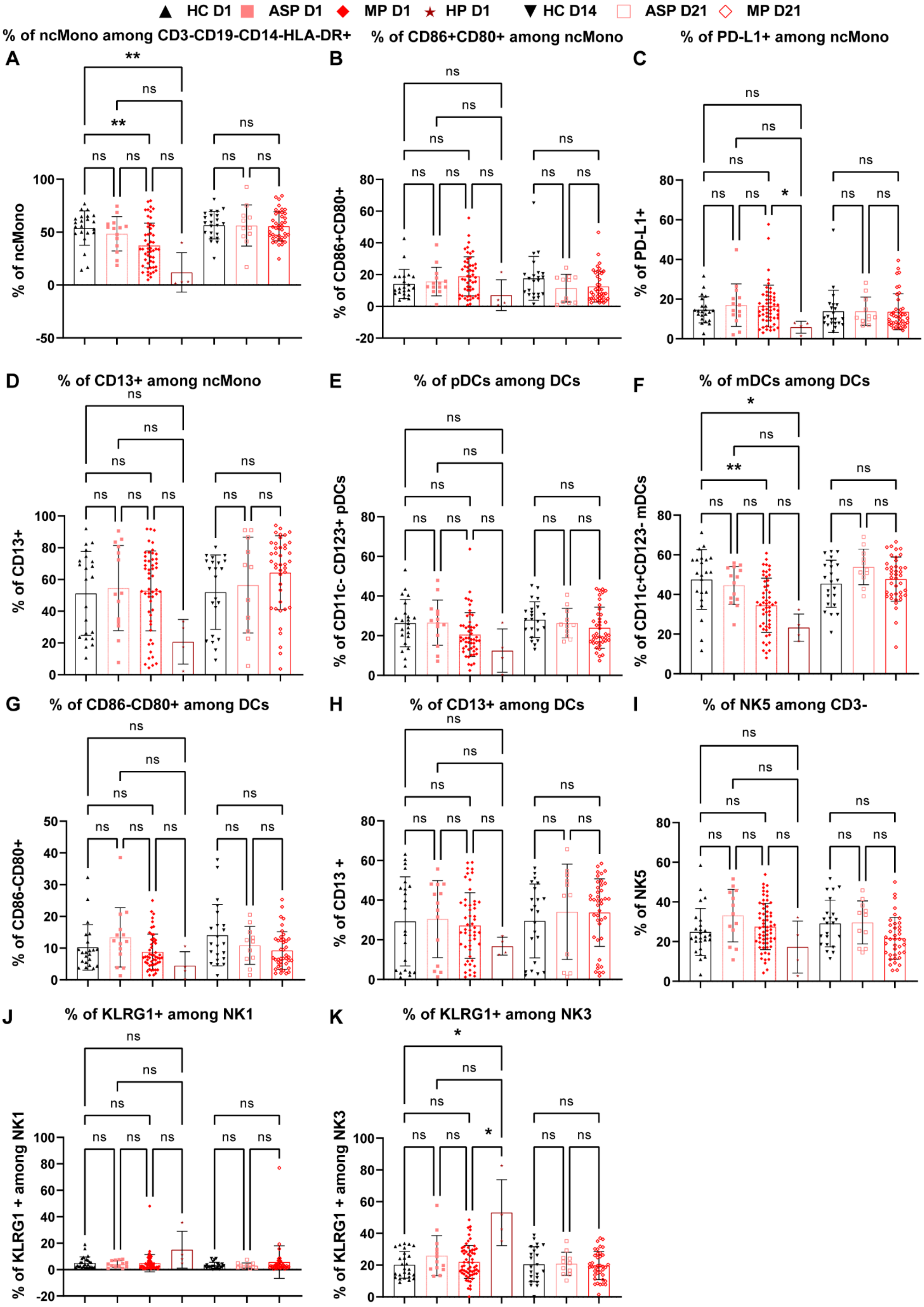

HC D1
ASP D1
MP D1
HP D1
HC D14
ASP D21
MP D21
A
% of ncMono among CD3-CD19-CD14-HLA-DR+
% of ncMono
B
% of CD86+CD80+ among ncMono
% of CD86+CD80+
C
% of PD-L1+ among ncMono
% of PD-L1+
D
% of CD13+ among ncMono
% of CD13+
E
% of pDCs among DCs
% of CD11c- CD123+ pDCs
F
% of mDCs among DCs
% of CD11c+CD123- mDCs
G
% of CD86-CD80+ among DCs
% of CD86-CD80+
H
% of CD13+ among DCs
% of CD13 +
I
% of NK5 among CD3-
% of NK5
J
% of KLRG1+ among NK1
% of KLRG1 + among NK1
K
% of KLRG1+ among NK3
% of KLRG1 + among NK3

**Figure S6. Sub-cohort analysis on impaired early-stage responses of non-classical monocytes, DC and NK cells in four hospitalized COVID-19 patients with a shorter prodromal phase.**

**A, B, C, D**, Percentage of ncMono (non-classical monocytes) among CD3-CD19-CD14-HLA-DR+ cells (**A**), percentages of CD86+CD80+ population among ncMono (**B**), percentage of PD-L1+ subset (**C**) or CD13+ population (**D**) among ncMono in the selected four HP at D1 in comparison with other groups. To ensure the comparability in terms of infection stage between different severity groups, we only selected four HP with at most 7-day delay from the onset of first symptoms to the inclusion day in this Supplementary Figure.
**E, F**, Percentage of pDCs (**E**) or mDCs (**F**) among total HLA-DR+CD38+ DCs (for gating strategy, refer to Fig. S2)
**G**, **H**, Percentage of CD86-CD80+ subset (**G**) or CD13+ cells (**H**) among total HLA-DR+CD38+ DCs.
**I**, Percentage of NK5 among CD3- cells
**J, K**, Percentage of KLRG1+ subset among NK1 cells (**J**) or among NK3 cells (**K**).
Of note, the values of this figure might be identical to that of the related figures within all the groups except for HP at D1. As argued above and in the text, this figure is not resulted by the duplicated usage of the same data, but to show a different analysis strategy. Data represent individual values from all biological replicates; Mean± standard deviation (S.D.); P-value was determined by the Kruskal-Wallis (nonparametric) test and corrected using the Dunn's multiple comparisons test. ASP, asymptomatic patients, n=14; HC, household controls, n=26; HP, hospitalized patients, n=4; MP, mild patients, n=63; D1/D14/D21, day 1/day 14/day 21. ns, not significant, *$p<=0.05$, **$p<=0.01$ and ***$p<=0.001$. Related to Figure 3.

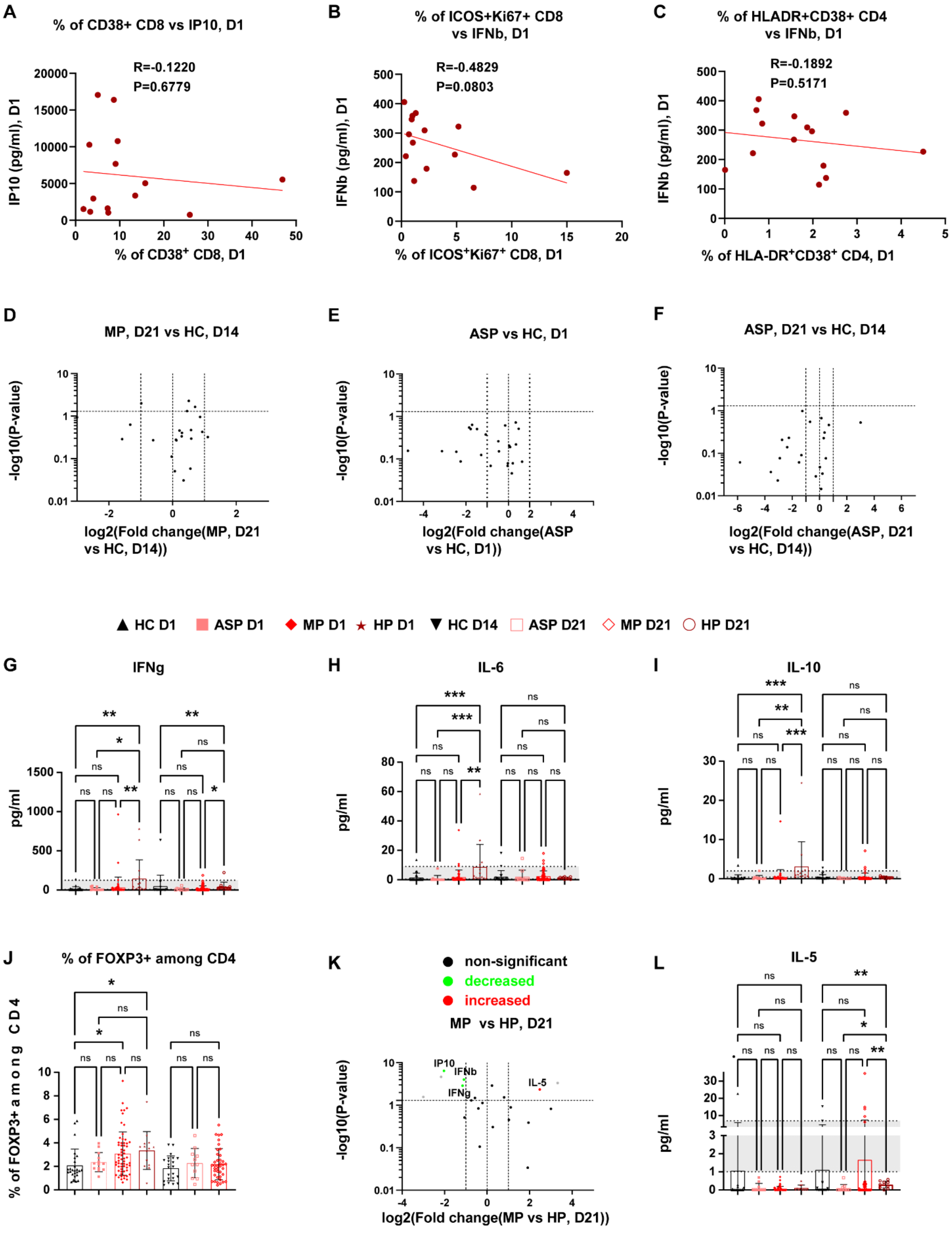

A
% of CD38+ CD8 vs IP10, D1
R=-0.1220
P=0.6779
IP10 (pg/ml), D1
% of CD38+ CD8, D1
B
% of ICOS+Ki67+ CD8 vs IFNb, D1
R=-0.4829
P=0.0803
IFNb (pg/ml), D1
% of ICOS+Ki67+ CD8, D1
C
% of HLADR+CD38+ CD4 vs IFNb, D1
R=-0.1892
P=0.5171
IFNb (pg/ml), D1
% of HLA-DR+CD38+ CD4, D1
D
MP, D21 vs HC, D14
-log10(P-value)
log2(Fold change(MP, D21 vs HC, D14))
E
ASP vs HC, D1
-log10(P-value)
log2(Fold change(ASP vs HC, D1))
F
ASP, D21 vs HC, D14
-log10(P-value)
log2(Fold change(ASP, D21 vs HC, D14))
HC D1
ASP D1
MP D1
HP D1
HC D14
ASP D21
MP D21
HP D21
G
IFNg
pg/ml
H
IL-6
pg/ml
I
IL-10
pg/ml
J
% of FOXP3+ among CD4
% of FOXP3+ among CD4
K
non-significant
decreased
increased
MP vs HP, D21
IP10
IFNb
IFNg
IL-5
-log10(P-value)
log2(Fold change(MP vs HP, D21))
L
IL-5
pg/ml

## Figure S7. Extended comparison of serological cytokine/chemokine responses between different participant groups at day 1 or day 21 following inclusion.

**A, B**, Correlation between the frequency of CD38+ cells among total CD8 T cells and circulating IP10 levels (**A**), or between the frequency of ICOS+Ki67+ among total CD8 T cells and circulating IFN-β levels (**B**) in HP at D1 of inclusion. R, Pearson correlation coefficient. Different groups were marked by different indicated colours.
**C**, Correlation between the frequency of HLA-DR+CD38+ cells among total CD4 T cells and circulating IFN-β levels in HP at D1. Pearson correlation coefficient.
**D**, Volcano plot showing 24 cytokine/chemokine responses of MP at D21 versus HC at D14 of inclusion.
**E**, Volcano plot showing 24 cytokine/chemokine responses of ASP versus HC at D1 following inclusion.
**F**, Volcano plot showing 24 cytokine/chemokine responses of ASP at D21 versus HC at D14 of inclusion. The gray dot represents the analytes showing a significant change but displaying values lower than the reported normal physiological levels in different groups, even in HC.
**G, H, I, L,** Scatter dot plots of IFN-γ (**G**), IL-6 (**H**), IL-10 (**I**) and IL-5 (**L**) of different participant groups.
**J**, Frequency of $FOXP3^+$ Treg cells among total CD4 T cells.
**K**, Volcano plot showing 24 cytokine/chemokine responses of MP versus HP at D21 following inclusion.
Data represent individual values from all biological replicates; Mean± standard deviation (S.D.); P-value was determined by the Kruskal-Wallis (nonparametric) test and corrected using the Dunn's multiple comparisons test. ASP, asymptomatic patients, n=14; HC, household controls, n=26; HP, hospitalized patients, n=15; MP, mild patients, n=63; D1/D14/D21, day 1/day 14/day 21. ns , not significant, *p<=0.05, **p<=0.01 and ***p<=0.001. Gray shading indicates the reported normal range for different cytokines. Related to Figure 4.

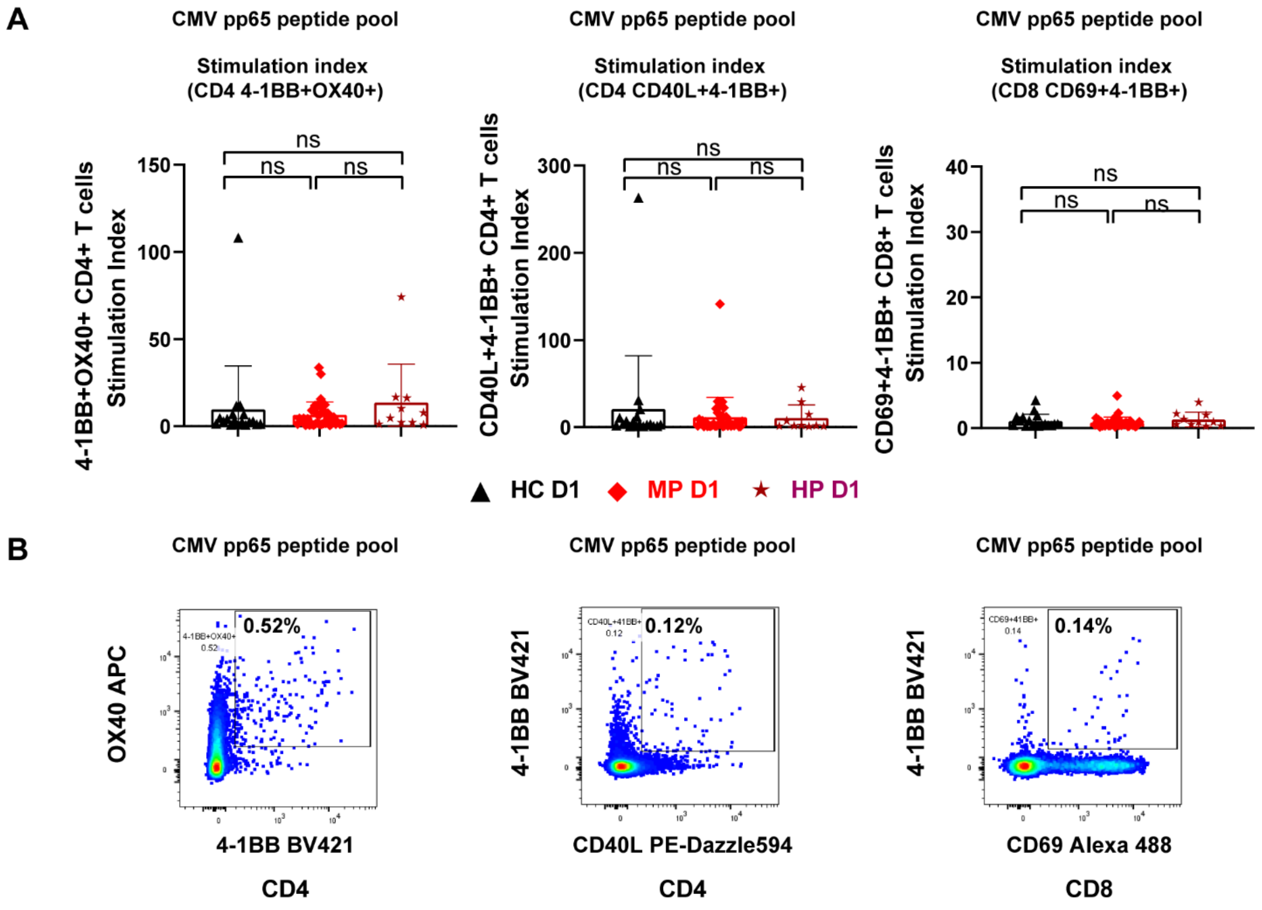


**Figure S8. No difference in the AIM response to the restimulation of the CMV peptide pool between household controls, mild and hospitalized patients at day 1.**

**A**, Normalized CMV-specific response (stimulation index, SI) among total CD4 T cells quantified by 4-1BB/CD137 and OX40/CD134 double positive cells (left panel), among total CD4 T cells quantified by CD40L and 4-1BB double positive cells (middle panel), or among total CD8 T cells quantified by CD69 and 4-1BB double positive cells (right panel) following stimulation with the CMV pp65 peptide pool or negative control in HC, MP or HP at D1. The normalized value (SI) was calculated using the original percentage in the presence of the CMV peptide pool divided by the percentage in negative control (H20) of the same sample (also refer to **STAR Methods**).
**B**, Representative flow-cytometry plots of 4-1BB/CD137 and OX40/CD134 among CD4 T cells (left panel), of CD40L and 4-1BB among CD4 (middle panel), or of CD69 and 4-1BB among CD8 T cells (right panel).
Data represent individual values from all biological replicates. Mean± standard deviation (S.D.). P-value from **A** was determined by non-paired two-tailed Student *t* test. HC, household controls, n=18; HP, hospitalized patients, n=10; MP, mild patients, n=40; D1, day 1. ns, not significant, *p<=0.05, **p<=0.01 and ***p<=0.001. Related to **STAR Methods** and Figure 6.

# Supplementary Tables

**Table S1. Demographics of our analysed longitudinal COVID-19 cohort.**

| Clinical characteristics of the cohort | All patients, D1 | Asymptomatic, D1 | Mild, D1 | Hospitalized, D1 | Household controls, D1 | All patients, D21 | Asymptomatic, D21 | Mild, D21 | Household Controls, D14 |
|---|---|---|---|---|---|---|---|---|---|
| **median (IQR)** | | | | | | | | | |
| **Age (years)** | 44 (30-53) | 44 (24.5-55.5) | 38 (28.5-48) | 57 (49-61) | 33.5 (26-40.75) | 40.5 (30.8-48.5) | 44 (29-53) | 40 (31-48) | 33.5 (27-42.25) |
| **BMI** | 25.7 (23.5-28.6) | 24.8 (22.9-26.6) | 25.8 (23.5-29.4) | 26.64 (24.9-29.1) | | 25.9 (23.9-27.9) | 24.8 (22.3-26.1) | 26.3 (24.4-28.7) | |
| **concerned n (percentage among the given category)** | | | | | | | | | |
| **Male (%)** | 54 (63.5%) | 9 (69.2%) | 39 (63.9%) | 8 (57.1%) | 7 (30.4%) | 33 (66%) | 8 (72.7%) | 25 (64.1%) | 7 (35%) |
| **Smoking (%)** | 14 (18.7%) | 3 (23.1%) | 11 (18.1%) | | | 9 (18%) | 2 (18.2%) | 7 (18%) | |
| **Former smoker (%)** | 16 (21.4%) | 2 (15.4%) | 14 (23%) | | | 12 (24%) | 2 (18.2%) | 10 (25.7%) | |
| **Whole Blood Count parameters: median (IQR)** | | | | | | | | | |
| **WBC, white blood cell count (10E3/uL)** | 5.25 (3.9-7.625) | 5.3 (4.4-6.1) | 4.6 (3.5-5.7) | 9.6 (8.85-11.05) | | | | | |
| **#Lymphocyte, LYM (10E3/uL)** | 1.65 (1.2-2.1) | 1.8 (1.4-2.1) | 1.6 (1.2-2.1) | 1.6 (1-2.1) | | | | | |
| **%Lymphocyte, LYM (%)** | 34.05 (25.575-40.75) | 33 (29.8-37.7) | 36.9 (31-43.9) | 16.8 (10.95-21.3) | | | | | |
| **#Monocyte, MON (10E3/uL)** | 0.3 (0.2-0.6) | 0.3 (0.28-0.43) | 0.2 (0.2-0.4) | 1.2 (0.7-2) | | | | | |
| **%Monocyte, MON (%)** | 7.3 (6.4-9.65) | 7.15 (6.5-7.9) | 6.9 (5.8-8.5) | 12.8 (9.7-17.1) | | | | | |
| **#Granulocyte, GRA (10E3/uL)** | 3.05 (2.175-4.6) | 3.35 (2.5-3.8) | 2.6 (1.8-3.8) | 7.2 (6.25-7.55) | | | | | |
| **%Granulocyte, GRA (%)** | 58.1 (50.9-65.2) | 59.7 (52.1-63) | 54.7 (48.7-62.7) | 67.8 (64.15-77.05) | | | | | |
| **Red Blood Cell, RBC (10E6/uL)** | 5.265 (4.865-5.7125) | 5.41 (5.0-5.7) | 5.31 (5.1-5.9) | 4.73 (4.335-4.945) | | | | | |

| | | | | | | | | | |
|---|---|---|---|---|---|---|---|---|---|
| **Hemoglobin, HGB (g/dl)** | 15.5 (14.1-17.025) | 15.6 (15.1-17.4) | 15.8 (14.7-17) | 13.9 (13.05-14.85) | | | | | |
| **Hematocrit, HCT (%)** | 48.25 (44.05-52.65) | 49.1 (46.9-54.4) | 48.8 (45.6-52.8) | 42.9 (39.5-45.7) | | | | | |
| **Platelet, PLT (10E3/uL)** | 175 (144-212) | 179.5 (154.8-209.3) | 167 (130-199) | 235 (163-286.5) | | | | | |
| **Mean Cell Volume, MCV (um3)** | 92 (89-94) | 92.5 (89.8-95) | 92 (89-94) | 91 (90-93) | | | | | |
| **Mean Cell Hemoglobin, MCH (pg)** | 29.35 (28.5-30.85) | 29.35 (28.5-30.3) | 28.9 (28.5-30.7) | 30.4 (29.9-31) | | | | | |
| **Mean Cell Hemoglobin Concentration, MCHC (g/dL)** | 32.1 (31.6-32.6) | 31.9 (31.8-32.4) | 32 (31.4-32.4) | 33.3 (33-33.5) | | | | | |
| **Red Cell Distribution Width, RDW (%)** | 14.35 (14-14.7) | 14.45 (14.1-14.6) | 14.4 (14.1-14.8) | 14.1 (13.95-14.55) | | | | | |
| **Mean Platelet Volume, MPV (um3)** | 8.4 (8-8.8) | 8.35 (8.1-9.0) | 8.4 (8-8.7) | 8.3 (8-8.6) | | | | | |
| **C-reactive protein, CRP (mg/L)** | 0.65 (0-10.075) | 0 (0-0.03) | 0.4 (0-1.6) | 38 (18.4-58) | | | | | |
| **Comorbidity: concerned n (the percentage among the given category)§** | | | | | | | | | |
| **Hypertension** | 8(10.7%) | 2(15.4%) | 6(9.9%) | | | 6(12%) | 1(9.1%) | 5 (12.9%) | NA* |
| **Chronic heart disease, including congenital heart disease (except hypertension)** | 5(6.7%) | 1(7.7%) | 4(6.6%) | | | 2 (4%) | 1(9.1%) | 1(2.6%) | NA |
| **Chronic lung disease (except asthma)** | 0 (0%) | 0 (0%) | 0 (0%) | | | 0 (0%) | 0 (0%) | 0 (0%) | NA |
| **Asthma (clinical diagnosis made)** | 3 (4%) | 0 (0%) | 3 (5%) | | | 0 (0%) | 0 (0%) | 0 (0%) | NA |
| **Chronic kidney disease** | 0 (0%) | 0 (0%) | 0 (0%) | | | 0 (0%) | 0 (0%) | 0 (0%) | NA |
| **Renal failure requiring dialysis** | 0 (0%) | 0 (0%) | 0 (0%) | | | 0 (0%) | 0 (0%) | 0 (0%) | NA |
| **Moderate or severe liver disease** | 1(1.4%) | 0 (0%) | 1 (1.7%) | | | 1 (2%) | 0 (0%) | 1(2.6%) | NA |
| **Mild liver disease** | 1(1.4%) | 1 (7.7%) | 0 (0%) | | | 1 (2%) | 1 (9.1%) | 0 (0%) | NA |
| **Chronic neurological disorder** | 1(1.4%) | 0 (0%) | 1 (1.7%) | | | 0 (0%) | 0 (0%) | 0 (0%) | NA |

| | | | | | | | | | |
|---|---|---|---|---|---|---|---|---|---|
| **Cancer** | 0 (0%) | 0 (0%) | 0 (0%) | | | 0 (0%) | 0 (0%) | 0 (0%) | NA |
| **Chronic hematologic disease** | 3 (4%) | 0 (0%) | 3 (5%) | | | 1 (2%) | 0 (0%) | 1(2.6%) | NA |
| **HIV / AIDS** | 0 (0%) | 0 (0%) | 0 (0%) | | | 0 (0%) | 0 (0%) | 0 (0%) | NA |
| **Obesity (clinical diagnosis made)** | 4(5.4% ) | 0 (0%) | 4 (6.6%) | | | 4 (8%) | 0 (0%) | 4(10.3 %) | NA |
| **Diabetes with associated complications** | 0 (0%) | 0 (0%) | 0 (0%) | | | 0 (0%) | 0 (0%) | 0 (0%) | NA |
| **Uncomplicated diabetes** | 5 (6.7%) | 0 (0%) | 5 (8.2%) | | | 2 (4%) | 0 (0%) | 2(5.2%) | NA |
| **Rheumatologic disease** | 1 (1.4%) | 0 (0%) | 1 (1.7%) | | | 1 (2%) | 0 (0%) | 1(2.6%) | NA |
| **Malnutrition** | 1(1.4% ) | 0 (0%) | 1 (1.7%) | | | 1 (2%) | 0 (0%) | 1(2.6%) | NA |
| **Chronic obstructive pulmonary disease (COPD)** | 1(1.4% ) | 0 (0%) | 1 (1.7%) | | | 0 (0%) | 0 (0%) | 0 (0%) | NA |
| **Other notable diseases or risk factors** | 12(16% ) | 1 (7.7%) | 11(18.1 %) | | | 8 (16%) | 0 (0%) | 8(20.6 %) | NA |

§, the total number of patients here only refer to asymptomatic and mild patients, but not hospitalized patients.
*, NA or empty, no information available.
IQR: 25% percentile-75% percentile.
n, the number of participants analyzed in the given category.
Related to Figure 1 and **STAR Methods**.
