# Supplementary material for: Combinatorial analysis reveals highly coordinated early-stage immune reactions that predict later antiviral immunity in mild COVID-19 patients
Source: Cell Rep Med. 2022 Mar 29;3(4):100600. doi: 10.1016/j.xcrm.2022.100600 (PMC8960124; doi:10.1016/j.xcrm.2022.100600)
Supplement: Document S2. Article plus supplemental information [file mmc2.pdf]

Cell Reports Medicine

Article

# Combinatorial analysis reveals highly coordinated early-stage immune reactions that predict later antiviral immunity in mild COVID-19 patients

## Graphical abstract

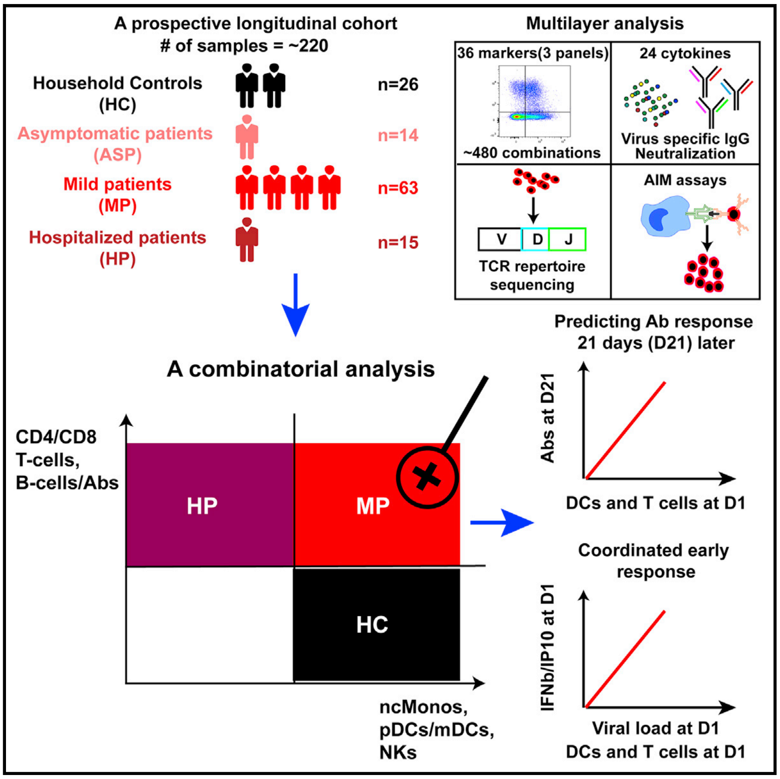


## Highlights

- Highly coordinated early-stage immune responses are confined to mild patients
- Early immune reactions can predict Ab responses 3 weeks later in mild patients
- Transient early surge of IFN-β and IP10 correlates with viral load in mild patients
- Dominant virus-specific CD4 T cells appear in both mild and hospitalized patients


## Authors

Christophe M. Capelle, Séverine Ciré, Olivia Domingues, ..., Guy Fagherazzi, Markus Ollert, Feng Q. Hefeng

## Correspondence

markus.ollert@lih.lu (M.O.), feng.he@lih.lu (F.Q.H.)



## In brief

In a parallel, longitudinal, prospective cohort based on a general population, using a systems-immunology analysis approach, Capelle et al. provide a comprehensive resource of multi-layered early immunological responses in mild COVID-19 patients. They reveal that a combination of highly coordinated early-stage immune responses is unique to mild patients.


Capelle et al., 2022, Cell Reports Medicine *3*, 100600
April 19, 2022 © 2022 The Author(s).
https://doi.org/10.1016/j.xcrm.2022.100600

CellPress

Cell Reports Medicine

CellPress
OPEN ACCESS

Article

# Combinatorial analysis reveals highly coordinated early-stage immune reactions that predict later antiviral immunity in mild COVID-19 patients

Christophe M. Capelle,[1,2,15] Séverine Ciré,[1,15] Olivia Domingues,[1] Isabelle Ernens,[3] Fanny Hedin,[4] Aurélie Fischer,[3] Chantal J. Snoeck,[1] Wim Ammerlaan,[5] Maria Konstantinou,[4] Kamil Grzyb,[6] Alexander Skupin,[6,7] Cara L. Carty,[8] Christiane Hilger,[1] Georges Gilson,[9] Aljosa Celebic,[10] Paul Wilmes,[2,6] Antonio Del Sol,[6,11,12] Ian M. Kaplan,[8] Fay Betsou,[5,13] Tamir Abdelrahman,[13] Antonio Cosma,[4] Michel Vaillant,[10] Guy Fagherazzi,[3] Markus Ollert,[1,14,16,*] and Feng Q. Hefeng[1,16,17,*]

[1]Department of Infection and Immunity, Luxembourg Institute of Health (LIH), Esch-sur-Alzette, Luxembourg
[2]Faculty of Science, Technology and Medicine, University of Luxembourg, Esch-sur-Alzette, Luxembourg
[3]Department of Precision Health, Luxembourg Institute of Health, Strassen, Luxembourg
[4]National Cytometry Platform, Luxembourg Institute of Health, Esch-sur-Alzette, Luxembourg
[5]Integrated BioBank of Luxembourg (IBBL), Dudelange, Luxembourg
[6]Luxembourg Centre for Systems Biomedicine (LCSB), University of Luxembourg, Belvaux, Luxembourg
[7]Department of Neuroscience, University California San Diego, La Jolla, CA, USA
[8]Adaptive Biotechnologies, Seattle, WA, USA
[9]Centre Hospitalier de Luxembourg (CHL), Luxembourg, Luxembourg
[10]Translational Medicine Operations Hub, Competence Centre for Methodology and Statistics, Luxembourg Institute of Health, Strassen, Luxembourg
[11]CIC bioGUNE, Bizkaia Technology Park, Derio, Spain
[12]IKERBASQUE, Basque Foundation for Science, Bilbao, Spain
[13]Laboratoire National de Santé (LNS), Dudelange, Luxembourg
[14]Department of Dermatology and Allergy Center, Odense Research Center for Anaphylaxis (ORCA), University of Southern Denmark, Odense, Denmark
[15]These authors contributed equally
[16]Senior author
[17]Lead contact
*Correspondence: markus.ollert@lih.lu (M.O.), feng.he@lih.lu (F.Q.H.)
https://doi.org/10.1016/j.xcrm.2022.100600

## SUMMARY

While immunopathology has been widely studied in patients with severe COVID-19, immune responses in non-hospitalized patients have remained largely elusive. We systematically analyze 484 peripheral cellular or soluble immune features in a longitudinal cohort of 63 mild and 15 hospitalized patients versus 14 asymptomatic and 26 household controls. We observe a transient increase of IP10/CXCL10 and interferon-β levels, coordinated responses of dominant SARS-CoV-2-specific CD4 and fewer CD8 T cells, and various antigen-presenting and antibody-secreting cells in mild patients within 3 days of PCR diagnosis. The frequency of key innate immune cells and their functional marker expression are impaired in hospitalized patients at day 1 of inclusion. T cell and dendritic cell responses at day 1 are highly predictive for SARS-CoV-2-specific antibody responses after 3 weeks in mild but not hospitalized patients. Our systematic analysis reveals a combinatorial picture and trajectory of various arms of the highly coordinated early-stage immune responses in mild COVID-19 patients.

## INTRODUCTION

The immunopathology underlying severe COVID-19 has been thoroughly studied over the last 2 years, including antibody (Ab) responses, cellular immune subsets, cytokines, and chemokines that were linked to characteristics and outcome of the disease.[1–6] However, with few exceptions,[7] relatively little is known about the details of the immune response in mild patients (MP) and asymptomatic COVID-19 patients (ASP). Using profiling analyses of immune cell subsets, several studies have identified crucial alterations in hospitalized patients (HP) with severe symptoms versus HP with moderate disease, convalescent patients, and healthy controls. These studies, which mainly utilized flow cytometry or single-cell mRNA sequencing, have demonstrated a wide spectrum of abnormal immune responses to SARS-CoV-2.[3,8,9,10] However, ASP and/or MP have only rarely been focused upon in these studies to draw conclusions. Therefore, it is unclear whether early protective immune signatures are identifiable in ASP or MP, and how such immune signatures might compare with HP and


This is an open access article under the CC BY license (http://creativecommons.org/licenses/by/4.0/).

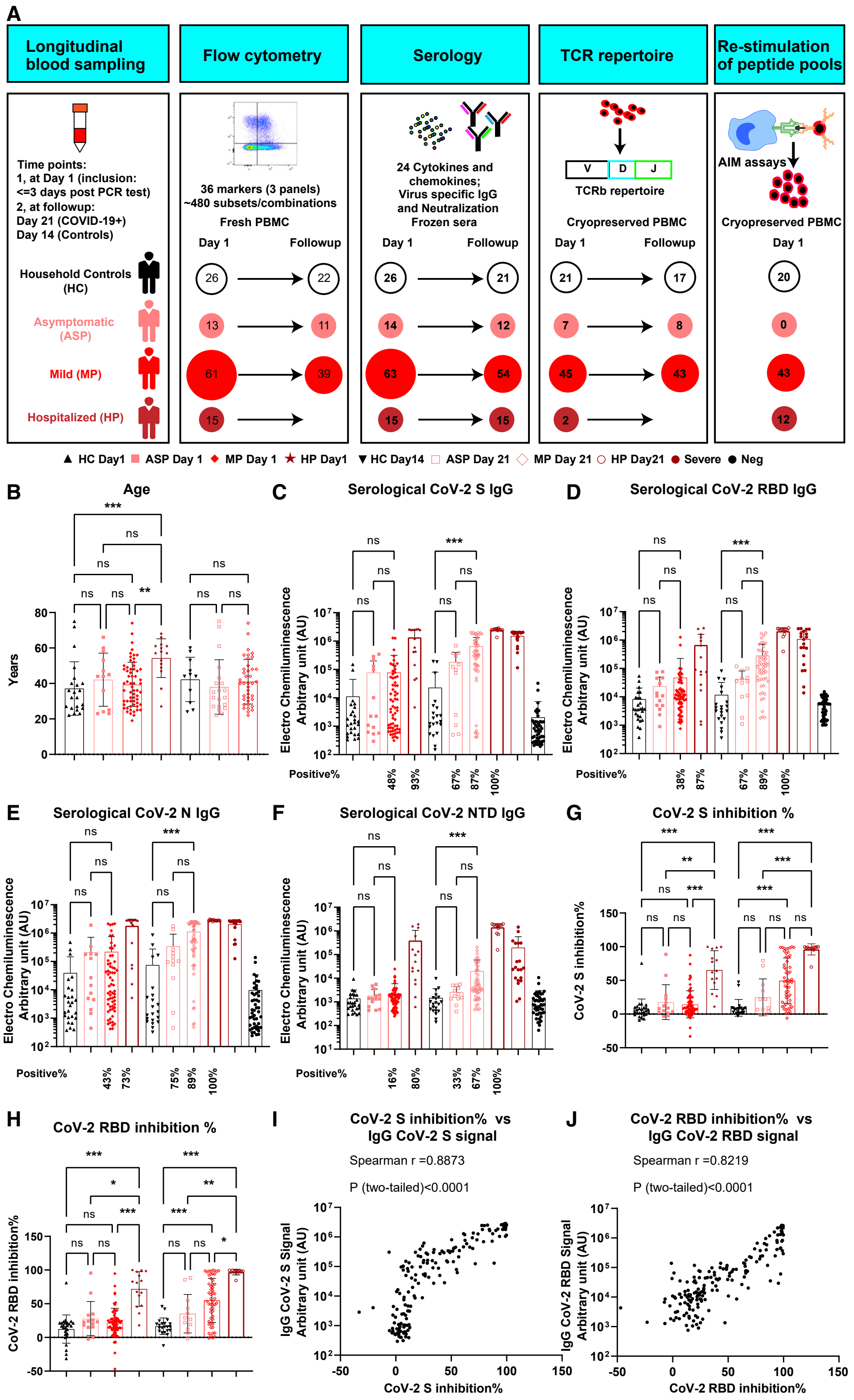


(legend on next page)

healthy people. Importantly, as reported cohort studies often lack a simultaneous analysis of the different aspects of multifaceted immune responses, it still remains unknown whether early-stage immune responses have any consequence for the evolution of other later immune reactions in MP.

Several studies have included ASP and MP in cross-sectional analyses. For example, Ab responses and several cytokines have been analyzed in ASP versus symptomatic patients.[11] Also, SARS-CoV-2-specific and functional memory T cells have been detected in recovered ASP and MP[12] or in recovered patients with undefined disease severity.[13] Such cross-sectional studies were critical to identifying dysregulated immune factors associated with severe COVID-19. However, the isolated analysis of specific cellular immune subsets or cytokines and Ab responses alone will only allow for a partial understanding of the complexity of the early immune trajectories following infection. Furthermore, owing to different kinetics of immune responses among various patient groups, only a head-to-head comparison in a longitudinal, prospective study design can guarantee the comparability of observations. Because of partial cohort aggregation and non-harmonized sampling time points, this was insufficiently addressed in another longitudinal project.[7]

In our longitudinal cohort with ~220 samples characterized by a parallel and prospective study design, we sought to address the aforementioned open questions. To this end, we simultaneously analyzed 484 immune combinations resulting from 36 lineage and functional markers in three multicolor flow-cytometry panels, 24 serological cytokine markers, Ab titers to SARS-CoV-2 spike (S), receptor-binding domain (RBD), N-terminal domain (NTD), and nucleocapsid (N), ACE2-binding inhibition to S and RBD as surrogate for Ab neutralization capacity, and SARS-CoV-2-specific T cells using both *ex vivo* T cell receptor β (TCR-β) repertoire sequencing and *in vitro* activation-induced marker (AIM) assays. Such a comprehensive, simultaneous, and integrated analysis in a longitudinal cohort using a systems-immunology strategy, as we and others have suggested in other contexts,[14,15] helps to draw a full picture of immune responses among MP.

## RESULTS

### Serological and whole blood count analysis distinguishing hospitalized from mild and asymptomatic COVID-19 patients

We established the longitudinal Predi-COVID cohort[16] in Luxembourg during the first wave of the pandemic with the aim of gaining a systematic understanding of the early antiviral immune response across the full spectrum of COVID-19 disease phenotypes (Figure 1A). All patients were included in Predi-COVID with a maximum delay of 3 days after a positive PCR test. According to available clinical data, patients were stratified into ASP (n = 14), mild to moderate (n = 63; referred to as MP in Figure 1A; for details, refer to STAR Methods), and HP (n = 15) subgroups for further analyses. All patients (n = 92) were sampled on the day of inclusion (D1) and 3 weeks after inclusion (D21). We also included control individuals (n = 26) from patients' households (HC), who were sampled on D1 and day 14 (D14). While the age was not different for ASP and MP as compared with HC (Table S1 and Figure 1B), HP were older than MP (median, ~57 versus ~38 years) and HC (Figure 1B). Body mass index was not different among any of the analyzed groups (Table S1). In general, more males were included in the patient subgroups (between 57% and 69%), while only around 30% of HC were male (Table S1). No comorbidity information was available for HP and HC. For the other patient groups, the prevalence of comorbidities (asthma, chronic hematologic disease, obesity, and uncomplicated diabetes) was higher among MP than ASP (5%–8% in MP versus none in ASP) (Table S1).

A whole blood count analysis was used to further characterize COVID-19 patients on D1. We found no significant difference between ASP and MP in any of the tested 17 general blood count parameters (Table S1 and Figure S1). However, HP showed a remarkable difference compared with MP or ASP as demonstrated in the principal component analysis (PCA) plot (Figure S1A). In line with other reports,[17] the frequency of lymphocytes was substantially decreased in HP versus the two other groups (Figure S1B), while C-reactive protein (CRP) was elevated in HP only (Figure S1C). Furthermore, compared with other groups, HP showed a significant but modest increase in the number or frequency of white blood cells, monocytes, granulocytes, and platelets, whereas a significant but modest decrease in red blood count and hematocrit was observed (Figures S1D–S1K). Considering the fact that the time from the onset of first symptoms to diagnosis might take longer in severe cases, i.e., in HP, we further analyzed only those HP with a shorter prodromal phase that was comparable with MP. To this end, we performed a sub-cohort analysis by selecting HP (n = 4) with at most a 7-day delay from the appearance of the first symptoms to inclusion to match the average time interval of all the MP (for details, refer to STAR Methods). Encouragingly, both the CRP levels and the percentages of lymphocytes were still significantly higher or lower, respectively, among HP versus MP on D1 (Figures S1L and S1M).

**Figure 1. Cohort description and SARS-CoV-2 serological analysis of different groups**

(A) Cohort and experimental overview. ASP, asymptomatic patients, n = 14; HC, household controls, n = 26; HP, hospitalized patients, n = 15; MP, mild patients, n = 63; AIM, activation-induced marker.

(B) Scatterdot plots of age.

(C–F) SARS-CoV-2 spike (S)-specific (C), RBD-specific (D), N-specific (E), or NTD-specific (F) IgG titers at D1 for each group, D21 post inclusion for different patient groups, or D14 for HC. For more information, refer to STAR Methods.

(G and H) Inhibition percentages by anti-SARS-CoV-2 S (G) or RBD (H) Abs using a pseudo-neutralization assay.

(I and J) Correlation between Ab titers and inhibition percentages of S (I) or RBD (J) antigens in all COVID-19 patient samples.

Spearman's correlation was used. Data represent individual values from all biological replicates; mean ± SD. p values in (B) to (H) were determined by the Kruskal-Wallis (non-parametric) test and corrected using Dunn's multiple comparisons test. ns, not significant; *$p \leq 0.05$, **$p \leq 0.01$, ***$p \leq 0.001$. See also Figure S1.

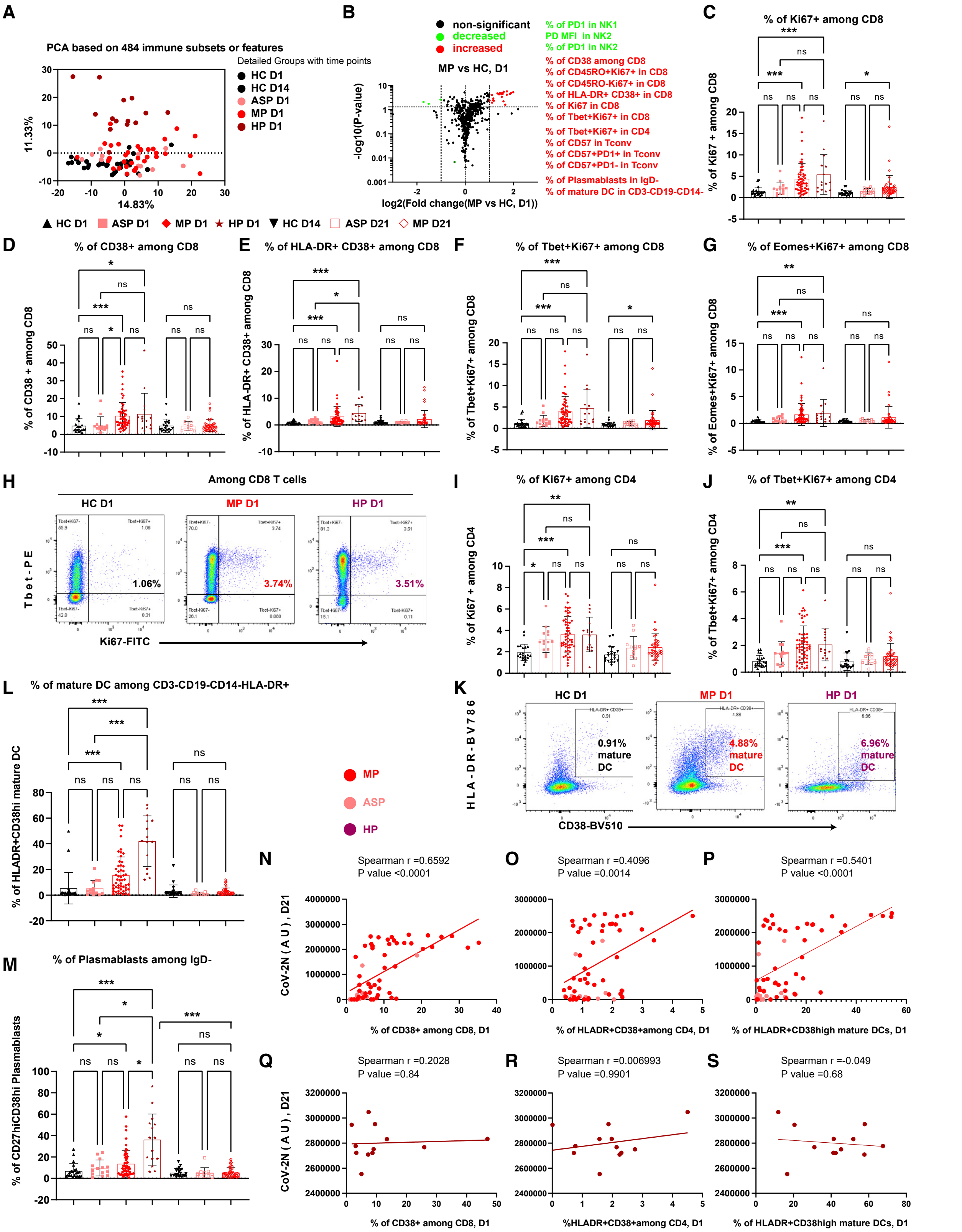


(legend on next page)

CellPress
OPEN ACCESS

As expected, on D1 we did not observe a significant increase in immunoglobulin G (IgG) Ab titers to SARS-CoV-2 S, RBD, N, and NTD in the non-hospitalized groups versus HC (Figures 1C–1F). In contrast, while only 48% and 43% of MP showed slightly increased IgG levels, 93% and 73% of HP already displayed significantly enhanced IgG titers to CoV-2 S and N antigens, respectively. Three weeks later, we observed a significant increase in IgG levels to all four antigens also in MP compared with HC together with a further enhancement of IgG levels in HP. The positivity rate for IgG Abs to CoV-2 S, RBD, NTD, or N reached up to 89% among MP and 100% among HP at D21. ASP had a lower positivity rate for IgG against S/RBD (67%) and N (75%) than MP at D21 (Figures 1C–1E). IgG Abs against NTD were in general much lower in ASP and MP, except for HP at D21 (Figure 1F). Next, we tested the functional capacity of the induced Abs in a surrogate virus neutralization assay. In line with the serology findings on D1 (Figures 1C and 1D), HP already showed blocking Abs that interfered with ACE2 binding to CoV-2 S or RBD at this early stage (Figures 1G and 1H). On D21, MP also had developed a significant inhibitory capacity to block ACE2 binding to CoV-2 S or RBD versus HC. Similar to the correlation reported by others,[18] IgG Ab titers against both CoV-2 S and RBD were highly correlated (Spearman's r = 0.89 and 0.82 for S and RBD, respectively) with the inhibitory capacity of sera from all the patients (Figures 1I and 1J).

## Early-stage highly coordinated innate and adaptive immune responses in mild COVID-19

As shown above, routine laboratory data as well as deep serologic profiling of SARS-CoV-2-specific Ab responses already distinguished HP from MP or ASP and HC. However, these analyses were not sufficient to further differentiate non-hospitalized clinical phenotypes. A meta-analysis has found that only 3% of the mean convalescent neutralizing Ab levels are necessary to predict protection from severe COVID-19,[19] but the same work has also indicated that those Abs are not sufficient to protect from severe cases.

Thus, we aimed to explore the full complexity of innate and adaptive cellular immune signatures that orchestrate the response to SARS-CoV-2 infection across the full spectrum of COVID-19 disease phenotypes. We systematically investigated 484 cellular immune subsets or combinations of various lineage and functional markers (Figure 1A) by three staining panels using 18-color flow cytometry (for general gating strategy, see Figure S2; for cellular markers analyzed, refer to key resources table) in our longitudinal cohort. When compared with HC, ASP displayed no obvious change in all of the analyzed 484 immune profiles at D1 (Figure S3A). Interestingly, at D21, ICOS$^+$ cells were the only significantly changed immune subset with a decrease in the frequency among CD8 T cells from ASP (Figures S3B and S3C).

We next used PCA to show that 484 immune features were only able to partition HP at D1 from all other groups, but not MP at D1 from any HC (both D1 and D14) (Figure 2A). We then asked whether specific immune subsets were differentially present in MP versus HC at D1 (Figure 2B). We observed differences in the frequency of several CD8 T cell subsets, such as Ki67$^+$, CD38$^+$, and HLA-DR$^+$CD38$^+$, representing proliferating, activated, and antigen-specific responsive CD8 T cells, respectively that were significantly enhanced in MP (Figures 2C–2E). The profile included an increase of both T-bet-dependent (T-bet$^+$Ki67$^+$) and -independent (EOMES$^+$Ki67$^+$) responsive CD8 T cells (Figures 2F–2H). Also, the fraction of proliferating CD4 T cells, especially Th1-responsive (T-bet$^+$Ki67$^+$) cells, was already enhanced early on in MP on D1 (Figures 2I, 2J, and S4A). In parallel, the frequency of antigen-presenting cells (APCs) and Ab-secreting cells, such as mature dendritic cells (HLA-DR$^+$CD38$^{high}$ DCs) and short-lived plasmablasts (CD27$^+$CD38$^{high}$), was increased in MP (Figures 2K–2M, S4B, and S4C). Notably, the frequency of activated CD38$^+$ CD8 T cells, HLA-DR$^+$CD38$^+$ CD4 T cells, and mature DCs measured at D1 was highly predictive for the degree of the serological titers of anti-SARS-CoV-2 N IgG and other CoV-2 antigens at D21 among ASP and MP (Figures 2N–2P). Since the magnitude of the responses of CD4 T cells, CD8 T cells, and mature DCs at D1 was all highly correlated with the same parameter (i.e., Ab levels) at D21, the response levels of these three subsets at D1 should correlate with each other. Such early responses of those immune subsets were highly coordinated only in ASP and MP. On the contrary, it is noteworthy that neither the frequency of activated subsets among CD4 and CD8 T cells nor of mature

**Figure 2. Early-stage coordinated responses of T cells, mature DCs, and plasmablasts in mild patients**

(A) PCA plots of the samples from different groups.

(B) Volcano plots of different immune features in MP versus HC. The selected list of significantly increased or decreased subsets ($p \leq 0.05$ and fold change $\geq 2$) are marked in red and green, respectively.

(C–G) Frequency of Ki67$^+$ cells (C), CD38$^+$ cells (D), HLA-DR$^+$CD38$^+$ cells (E), T-bet$^+$Ki67$^+$ cells (F), and EOMES$^+$Ki67$^+$ cells (G) among CD8 T cells.

(H and K) Representative flow-cytometry plots of the expression of T-bet and Ki67 on CD8 T cells (H) or the expression of CD38 and HLA-DR (K) at D1.

(I and J) Frequency of Ki67$^+$ cells (I) and T-bet$^+$Ki67$^+$ cells (J) among CD4 T cells.

(L and M) Frequency of HLA-DR$^+$CD38$^{high}$ (mature DC) among CD3$^-$CD19$^-$CD14$^-$HLA-DR$^+$ cells (L) and of CD27$^{high}$CD38$^{high}$ plasmablasts among CD3$^-$CD19$^+$IgD$^-$ B cells (M).

(N, O, Q, and R) Correlation between the frequency of CD38$^+$ among CD8 T cells or the frequency of HLA-DR$^+$CD38$^+$ cells among CD4 T cells at D1 and anti-SARS-CoV-2N-specific IgG titers at D21 from ASP and MP (N and O) or from HP (Q and R).

(P and S) Correlation between the frequency of mature DC among CD3$^-$CD19$^-$CD14$^-$HLA-DR$^+$ cells at D1 and anti-SARS-CoV-2N-specific IgG titers at D21 from ASP and MP (P) or from HP (S).

ASP, asymptomatic patients, n = 14; AU, arbitrary unit; HC, household controls, n = 26; HP, hospitalized patients, n = 15; MP, mild patients, n = 63; Tconv, FOXP3$^-$CD4 conventional T cells; D1/D14/D21, day 1/day 14/day 21. Data represent individual values from all biological replicates; mean ± SD. p values in (C–G), (I), (J), and (K–M) were determined by the Kruskal-Wallis (non-parametric) test and corrected using Dunn's multiple comparisons test. Spearman's correlation was used in (N) to (S). ns, not significant; *$p \leq 0.05$, **$p \leq 0.01$, ***$p \leq 0.001$. See also Figures S2–S4.

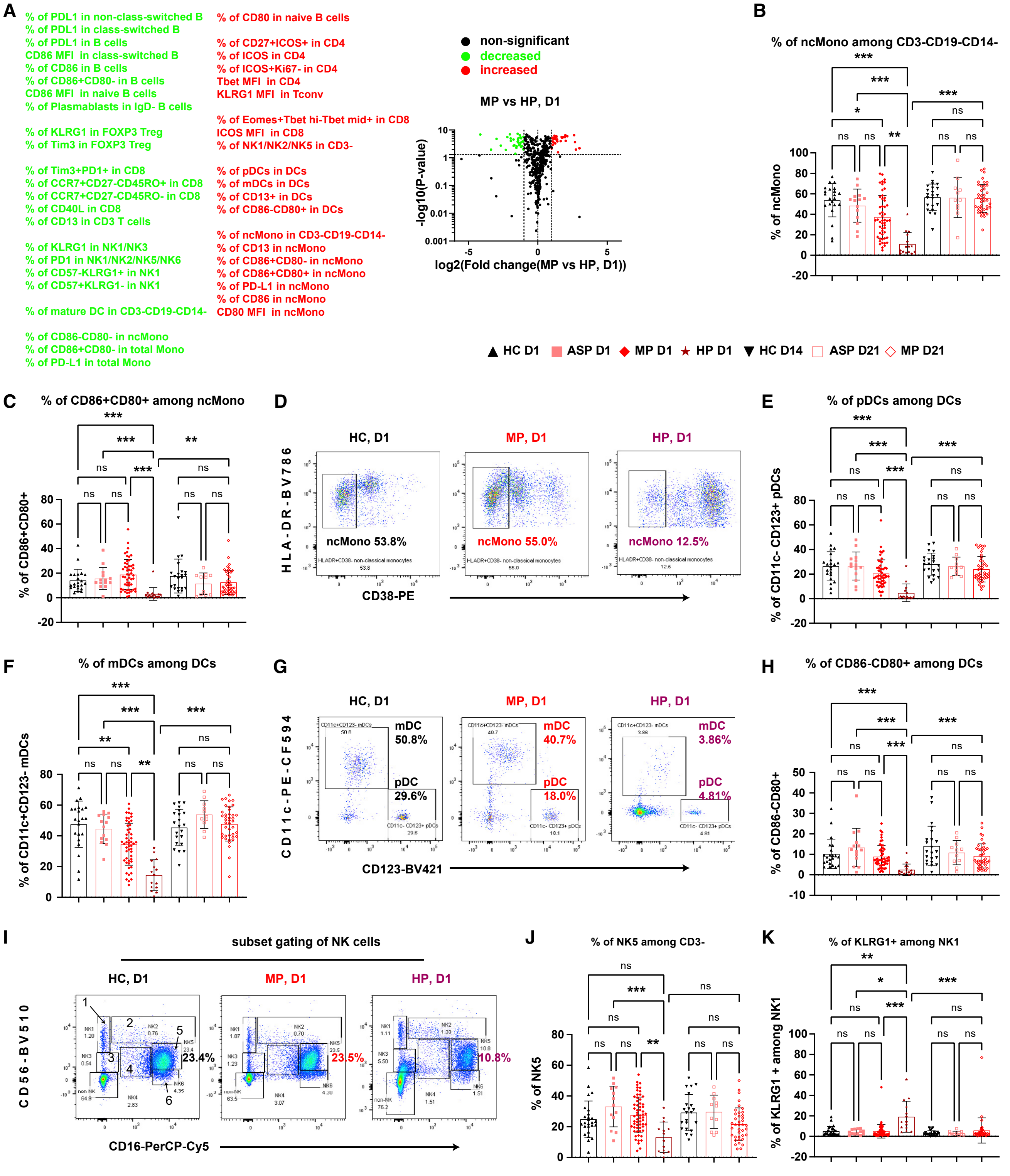


**Figure 3. Impaired early-stage responses of ncMono, DCs, and NKs distinguishing hospitalized from mild patients**

(A) Volcano plot showing comparison of the frequency of immune subsets in MP versus HP. The selected list of significant increased or decreased subsets ($p \leq 0.05$ and fold change $\geq 2$) are marked in red and green, respectively.

(B) Proportions of HLA-DR$^+$CD38$^-$ non-classical monocytes (ncMono) among CD3$^-$CD19$^-$CD14$^-$HLA-DR$^+$ cells (see also Figure S2).

(C, E, and F) Frequency of cells expressing CD86$^+$CD80$^+$ among ncMono (C), pDC (E), or mDC (F) among total DCs.

(legend continued on next page)

DCs at D1 was significantly correlated with the even higher titers of CoV-2 N IgG (Figures 2Q–2S) and other CoV-2 antigens in HP at D21. This finding indicates that the progression and deterioration of COVID-19 is averted only in the presence of a highly coordinated interplay of early innate and adaptive immune responses, which are strongly correlated with the subsequent production of Ab titers.

On D21, MP were characterized by an enhanced cytotoxic CD8 T cell ($GZMB^+$) response, especially of terminally differentiated responsive CD8 T cells ($CD45RO^-Ki67^+$) (Figures S3D–S3F). CD4 T cells also showed similar changes. The frequencies of CD4 T cells expressing CD57 or GZMB as well as of CD45RO and CD57 double-positive cells were also significantly enhanced in MP versus HC (Figures S3G–S3I). Notably, the frequency of $GZMB^+$ CD4 cytotoxic T cells showed a trend to be elevated ($p = 0.053$, Kruskal-Wallis test including multiple-group correction) already on D1 (Figure S3H). The CD57 expressing CD4 T cells detected on D21 appeared to be mainly cytotoxic effector cells, since the percentage of $GZMB^+CD57^+$ cells was also significantly enhanced among CD4 T cells (Figure S3J). Although CD57 is known as a T cell senescence marker, $CD57^+$ T cells, similar to the scenario of $PD\text{-}1^+$ T cells,[20] were apparently still functional during the acute phase of COVID-19, thus likely contributing to sufficient control of the infection in MP.

## Early-stage impaired innate immunity in hospitalized but not mild patients

Until now, we parsed primarily early immune signatures in MP in relation to HC on D1 and D21. Yet the analysis of early cellular responses and later Ab responses showed a positive correlation only in MP and ASP, but not HP (Figures 2N–2S). This finding prompted us to further compare between HP and other groups. Thus, we asked whether any additional early immune signatures observed in MP were significantly different from HP and determined the immune signatures that were significantly upregulated or downregulated in MP versus HP on D1 (Figure 3A). As shown in the volcano plot, major differences were present primarily among innate immune cells, such as monocytes, DCs, and natural killer (NK) cells, and to a lesser extent also among B and T cells. Compared with HP, MP showed a much higher frequency (~40% in MP versus ~10% in HP) of non-classical monocytes (ncMono, $HLA\text{-}DR^+CD38^-$).[21] The ncMono were not only higher in frequency among MP but also expressed higher levels of critical functional markers, such as CD86/CD80 double positivity (Figures 3B–3D), PD-L1, and CD13 (Figures S4D, S5A, and S5B). Similar to monocytes, the frequency of APCs such as plasmacytoid DCs (pDCs) and myeloid DCs (mDCs, also known as cDCs) was significantly higher in MP versus HP (Figures 3A and 3D–3G). It is noteworthy that the frequency of ncMono and mDCs (Figures 3B and 3F) was also slightly lower in MP than in HC, indicating a disease severity-related effect and further supporting the involvement of both cell types in early protective immune responses of COVID-19. Although mature DCs were higher in both MP and HP (Figure 2L), the frequency of $CD86^-CD80^+$ cells (Figures 3G and 3H) and of $CD13^+$ cells (Figure S5C) among total DCs was decreased in HP only, thus indicating a reduction in phagocytic and antigen-presenting capacity of individual DCs.[22] In line with the notion of reduced APC functions, the downstream events of APC activation, the frequency of activated CD4 T cells ($CD27^+ICOS^+$), and the ICOS mean fluorescence intensity (MFI) among CD8 T cells were decreased only in HP but not MP (Figures S5D and S5E). Furthermore, the frequency of NK cells was also significantly decreased only in HP, but not in MP versus HC (Figures 3I and 3J). In line with the overall compromised innate immune cell profile, critical senescence and exhaustion markers such as KLRG1 and PD-1 were enhanced among several NK subsets in HP only (Figures 3K, S4E, S5F, and S5G).

In contrast to the compromised innate immune compartment, the expression levels of CD86 and the percentages of $PD\text{-}L1^+$ cells among class-switched memory B cells were even substantially enhanced in HP versus both HC and MP at D1 (Figures S5H and S5I). Considering these results together with the high SARS-CoV-2-specific IgG titers, the ACE2-blocking capacity of patient serum, and the high frequency of plasmablasts (even higher than MP at both D1 and D21, Figure 2M), we concluded that Ab-secreting cells were not impaired in both MP and HP at D1, thus leading to a robust Ab response after 3 weeks.

We next sought to understand whether the impaired innate immune response in HP was paralleled by early deviated CD8 T cell profiles. Although the MFI of ICOS on total CD8 T cells was decreased (Figure S5E), the frequency of $ICOS^+$ cells was unchanged (Figure S3C) and the frequency of $CD40L^+$ and $PD\text{-}1^+GZMB^+$ cells among CD8 T cells was even significantly enhanced in HP on D1 (Figures S5J and S5K). Furthermore, since the frequency of CD8 T cells expressing other key functional markers was not decreased in HP (Figures 2C–2G), the functional antiviral capacity of CD8 T cells was most likely not compromised in HP versus MP. Overall, these data indicate that the major deficiencies observed in HP on D1 were impaired innate immune cells and their APC functions rather than adaptive immunity.

Due to the difference between the onset of first symptoms and the inclusion day among different groups (refer to the rationale described in the first part of this section regarding whole blood count analysis), we further performed a sub-cohort analysis by comparing HP with a shorter prodromal phase with all MP. We

(D and G) Representative flow-cytometry plots of the expression of HLA-DR and CD38 (D) or the expression of CD11c and CD123 (G).
(H) Frequency of $CD86^-CD80^+$ cells among DCs.
(I) Representative flow-cytometry plots of the expression of CD56 and CD16. Gating strategy to define six subsets of NK cells.
(J and K) Frequency of NK5 among $CD3^-$ cells (J) or of $KLRG1^+$ cells among NK1 (K).
ASP, asymptomatic patients, n = 14; HC, household controls, n = 26; HP, hospitalized patients, n = 15; MP, mild patients, n = 63; D1/D14/D21, day 1/day 14/day 21. Due to limited space, some subsets might be merged to shorten the list in (A). Data represent individual values from all biological replicates; mean ± SD. p values were determined by the Kruskal-Wallis (non-parametric) test and corrected using Dunn's multiple comparisons test. ns, not significant; $^*p \leq 0.05$, $^{**}p \leq 0.01$, $^{***}p \leq 0.001$. See also Figures S2 and S4–S6.

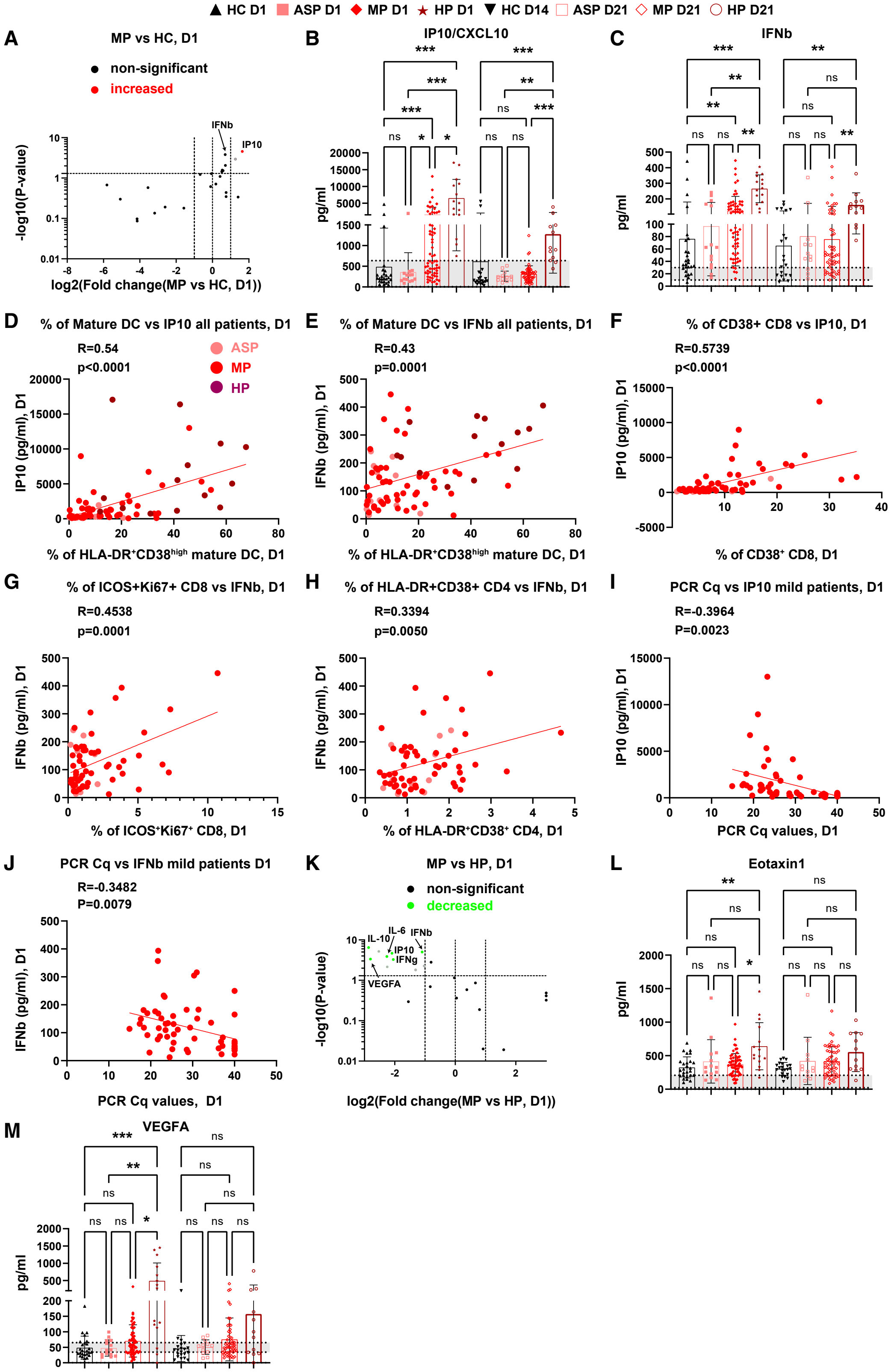


(legend on next page)

compared those HP (n = 4) with all MP (Figure S6). Although one could not expect a statistical significance in all the comparisons with a lower case number, the change trend between the four HP and all MP (Figures S6A–S6K) clearly matched the results displayed in the entire cohort. Notably, even with only four selected HP, the frequency of the ncMono expressing the key functional marker PD-L1 was still significantly lower than in MP at D1 (Figure S6C). This also held true for an increase of the inhibitory marker KLRG1 in one of the NK subsets (Figure S6K). To further substantiate our conclusions, we also compared innate immune responses of MP on D21 with those of HP on D1. All MP at D21 were in a later stage of their infection course than all HP at D1. Highly encouragingly, almost all the impaired critical immune subsets or functional markers (Figures 3 and S5) in HP at D1, except for the frequency of one NK subset (Figure 3J), remained significantly higher (or lower for inhibitory markers) in MP even at D21 versus HP at D1. In summary, our data firmly support that it is the early-stage response of innate immunity (including NK cells) that differentiates MP and HP during natural SARS-CoV-2 infection.

## Early-stage temporary elevation of IP10 and IFN-β in mild COVID-19 patients

To gain further insight into the coordinated early immune response of COVID-19, we analyzed 24 different cytokines (refer to STAR Methods) at both D1 and D21 in sera of all groups. Interestingly, at D1, we observed increased levels of interferon-γ (IFN-γ)-inducible protein 10 (IP10/CXCL10) in HP and MP (Figures 4A and 4B). Unexpectedly, a similar regulation was found for the type I interferon IFN-β, which was previously reported to be undetectable in severe COVID-19 patients at around 10 days after symptom onset.[23] Both MP and HP showed a significant increase of IFN-β compared with HC at D1 (Figure 4C). While the levels of IP10 and IFN-β showed only a temporary increase among MP, declining to normal levels at D21, both IP10 and IFN-β levels remained elevated in HP after 3 weeks (Figure 4C). These results point to a crucial and dynamic role of IP10 and IFN-β, which is tightly regulated during the early stage of protective immune responses in COVID-19 patients. This notion is also supported by the fact that levels of IP10 and IFN-β were significantly correlated with the frequency of mature DCs among all the analyzed patients at D1 (Figures 4D and 4E). Interestingly, the serological IP10 and IFN-β levels were significantly correlated with the magnitude of the CD8 T cell response among ASP and MP at D1 (Figures 4F and 4G). IFN-β levels were also correlated with the frequency of HLA-DR$^{+}$CD38$^{+}$ cells among CD4 T cells (Figure 4H), indicating that the temporary IFN-β surge was indeed correlated with T cell responses. Such a positive correlation did not exist in HP at D1 (Figures S7A–S7C), indicating again the importance of early-stage orchestrated immune responses preventing severe illness. The CD8 responses and IFN-β levels in HP even showed a trend to be negatively correlated ($r = -0.48$, $p = 0.08$, Figure S7B). Furthermore, both IP10 and IFN-β levels among MP significantly correlated with the SARS-CoV-2 viral load at D1 (Figures 4I and 4J), as demonstrated by a negative correlation with the PCR Cq values.

On D21, none of the 24 circulating immune analytes showed a significant change in MP versus HC at D14 (Figure S7D). We also could not observe any significant change among ASP versus HC at either D1 or D21 (Figures S7E and S7F). The increase of IP10 in MP and HP appears to be independent of IFN-γ, which was only elevated in HP at D1 (Figures 4B, 4K, and S7G) and remained high at D21, but still within the normal range for most of the patients (Figure S7G).

Regarding other circulating soluble factors, we found a significant and substantial increase in plasma levels of eosinophil chemotactic protein (eotaxin-1/CCL11) and vascular endothelial growth factor A (VEGFA) only in HP at D1 (Figures 4L and 4M). The enhanced levels of interleukin-6 (IL-6) and the regulatory cytokine IL-10 in HP versus MP on D1 were still mostly seen within the normal range (Figures S7H and S7I). The elevation of IL-10 levels was in line with an increased percentage of FOXP3$^{+}$ regulatory T cells in both MP and HP at D1 (Figure S7J). On D21, only IFN-β and IP10/CXCL10 remained significantly and substantially higher in HP versus MP (Figure S7K). The T helper 2 cytokine IL-5, which was not different on D1, was modestly decreased in HP versus MP on D21 (Figure S7L).

## Early-stage dominant expansion of CD4$^{+}$ SARS-CoV-2-specific T cells in mild patients

To identify the T cell response on a broader scale, we performed TCR-β sequencing analysis among 45 MP versus 8 ASP and 21

**Figure 4. Early-stage transient cytokine responses in mild patients**
(A) Volcano plot showing the serological cytokine responses in MP versus HC.
Significantly increased or decreased cytokines ($p \leq 0.05$ and fold change $\geq 2$) are marked in red and green, respectively. Due to its highly significant p value, IFN-β is also marked although its fold change was slightly less than 2. The gray dot represents the analytes showing a significant change but with values less than the reported normal levels even in HC.
(B and C) Scatterdot plots of serological levels of IP10 (B) and IFN-β (C).
(D and E) Correlation between the frequency of mature DC and IP10 (D) or IFN-β (E) levels.
(F and G) Correlation between the frequency of CD38$^{+}$ cells among total CD8 T cells and IP10 levels (F), or of ICOS$^{+}$Ki67$^{+}$ cells among total CD8 T cells and IFN-β levels (G) in ASP and MP.
(H) Correlation between the frequency of HLA-DR$^{+}$CD38$^{+}$ cells among total CD4 T cells and IFN-β levels in ASP and MP.
(I and J) Correlation between the viral PCR Cq values and IP10 (I) or IFN-β (J) levels among MP (not much viral load information for other groups).
(K) Volcano plot showing the responses of serological cytokines of MP versus HP.
(L and M) Scatterdot plots of Eotaxin1/CCL11 (L) and VEGFA (M).
ASP, asymptomatic patients, n = 14; HC, household controls, n = 26; HP, hospitalized patients, n = 15; MP, mild patients, n = 63; D1/D14/D21, day 1/day 14/day 21. Data represent individual values from all biological replicates; mean ± SD. p values were determined by the Kruskal-Wallis (non-parametric) test and corrected using Dunn's multiple comparisons test. Pearson's correlation coefficient was used in (D) to (J). ns, not significant; *$p \leq 0.05$, **$p \leq 0.01$, ***$p \leq 0.001$. Gray shading indicates the reported normal range. See also Figure S7.

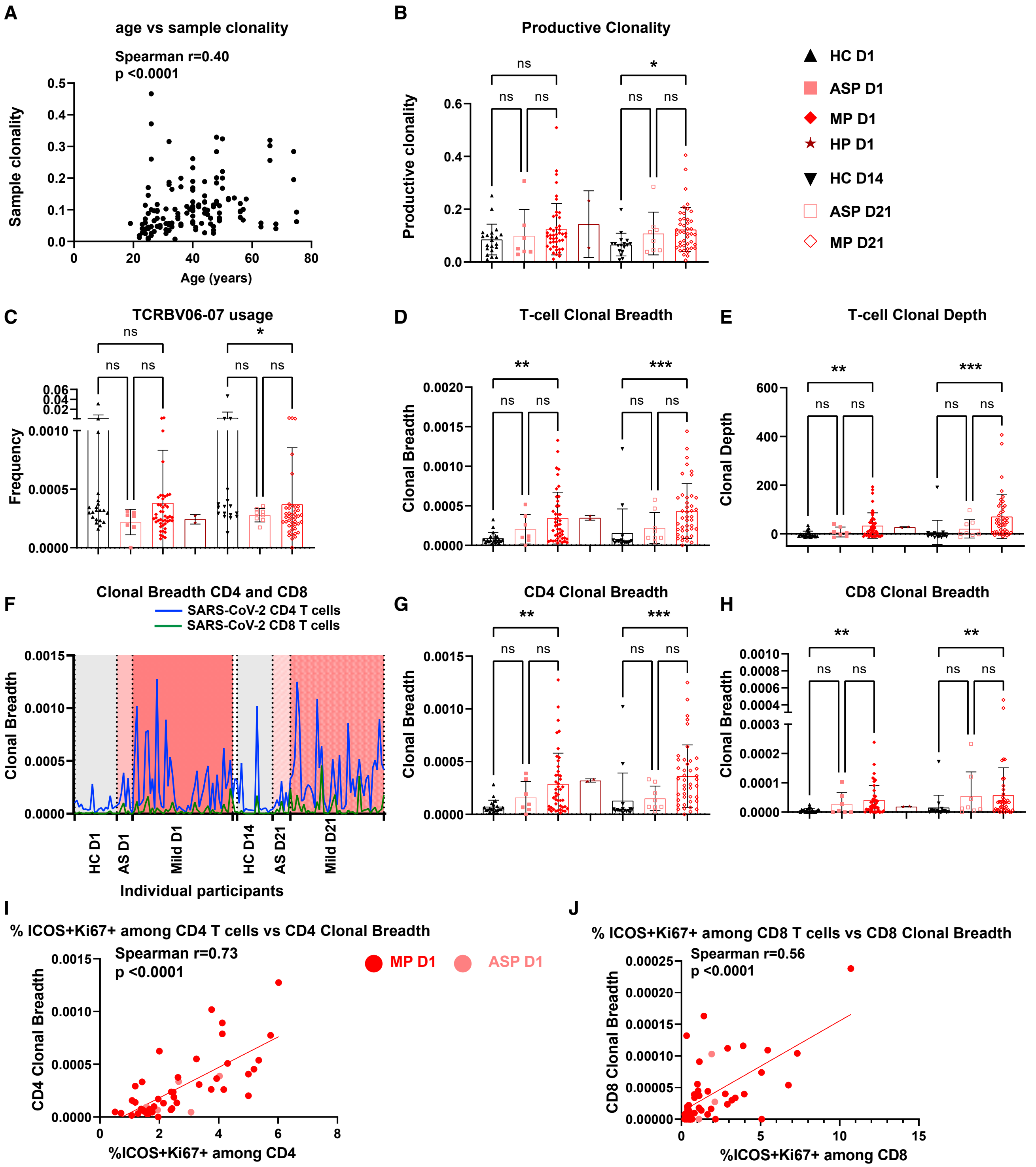


**Figure 5. Early-stage expansion of SARS-CoV-2-specific TCR clonotypes in mild patients**

(A) Correlation between sample clonality and age of corresponding participants (n = 144) from all groups.

(B) Productive clonality (inverted normalized diversity).

(C) Usage frequency of the TCRβ V06-07 gene.

(D and E) Clonal breadth (D) and clonal depth (E) of SARS-CoV-2-specific T cell clonotypes.

(F) Clonal breadth of inferred CD4 or CD8 SARS-CoV-2-specific TCR clonotypes of each individual participant. The individual values are linked through lines. The two D1 HP samples were positioned between mild D1 and HC D14 groups, but not labeled.

(legend continued on next page)

HC on D1 and D21. Aging has a strong impact on the TCR repertoire.[24] As expected, sample clonality, the inverted normalized diversity index, was significantly correlated with age (Figure 5A). Since a decrease in TCR diversity was previously associated with aging and impaired immunity against influenza virus infection and other diseases,[25,26] we sought to compare the TCR diversity between different groups. The productive clonality of the sequenced TCR-β repertoire was increased in MP at D21 versus HC (Figure 5B). At D21, only the use of one specific V gene (TCRBV06-07) was significantly under-represented in MP compared with HC (Figure 5C). Notably, the SARS-CoV-2-specific T cell clonotypes were substantially expanded (about six times higher than in HC) among MP already at D1, as reflected by both clonal breadth and depth,[27] and maintained at D21 (Figures 5D and 5E). These results indicate a key functional role of early-responsive SARS-CoV-2-specific T cells in MP. Unexpectedly, inferred expanded CD4 SARS-CoV-2-specific T cells showed an average frequency about six times higher than that of inferred CD8 SARS-CoV-2-specific TCR clonotypes among MP at D1 (Figures 5F–5H). This finding was in line with a trend for increased frequency of $GZMB^+$ cells among CD4 Tconv cells, but not among CD8 T cells, in MP versus HC on D1 (Figures S3D and S3H). At D21, inferred CD4 SARS-CoV-2-specific TCR clonotypes continued to dominate over CD8 clonotypes in MP (Figures 5G and 5H). The expansion of inferred CD4 or CD8 SARS-CoV-2-specific T cells was highly correlated with the frequency of responsive $ICOS^+Ki67^+$ cells among total CD4 or CD8 T cells in both ASP and MP at D1 (Figures 5I and 5J). These data highlight a crucial role of early-expanding SARS-CoV-2-specific T cells, especially of the $CD4^+$ cells, for subsequent coordinated antiviral immune responses. An earlier study has already reported SARS-CoV-2-specific T cells early after diagnosis.[28] However, the authors have not correlated their findings with a severity stratification of COVID-19 patients nor have they performed further sub-analysis of CD4 and CD8 T cells. Therefore, our findings advance the understanding of the early antiviral responses of T cell subsets, particularly in MP.

To further consolidate our observations on inferred SARS-CoV-2-specific T cells based on sequencing approaches,[27] we used another more direct experimental approach to analyze virus-specific T cells in a cytokine-independent but viral-peptide-specific way. To this end, the AIM assay was selected to independently quantify SARS-CoV-2-specific T cell responses. The AIM assay has been successfully used by others to quantify SARS-CoV-2-specific CD4 T cells by detecting 4-1BB and OX40 double-positive[29,30] or CD40L and 4-1BB double-positive cells.[31] Encouragingly, following stimulation with a peptide pool selected from the wild-type SARS-CoV-2 whole-virus proteome, the detected SARS-CoV-2-specific responses among CD4 T cells, as reflected by the stimulation index (SI) (i.e., after excluding background effects; for details refer to STAR Methods) of both AIM combinations, were indeed higher in D1 samples of MP versus HC (Figures 6A–6D). In line with our other T cell datasets (Figures 2C–2J) and humoral immune results (Figure 2M), the frequency of SARS-CoV-2-specific CD4 T cells, as quantified by both AIM combinations, was even higher in HP than in MP already at D1 (Figures 6B–6D).

SARS-CoV-2-specific CD8 responses were detected by calculating the portion of AIM ($CD69^+$4-1BB/$CD137^+$)[29,32] among total CD8 T cells. In line with our TCR sequencing data (Figures 5F–5H), the overall restimulation response (SI) for CD8 T cells was much lower than for CD4 T cells in both groups (Figures 6C–6E). Nevertheless, the SI for CD8 responses was still significantly higher in HP versus MP at D1 (Figures 6E and 6F). Contrary to CD4 T cells, we could not identify a significant difference between MP and HC for CD8 T cell responses (Figures 6E and 6F). As shown above using the sequencing method, a significant difference was observed between MP and HC (Figure 5H), and the CD8 T cell activation/proliferation response was highly correlated with the frequency of sequencing-derived SARS-CoV-2-specific T cells (Figure 5J). Notably, the frequency ($\sim 5 \times 10^{-5}$) of SARS-CoV-2-specific cells among total CD8 T cells detected using the robust tetramer approach[12] was in a range similar to that inferred by the TCR sequencing approach here. This indicates that the *ex vivo* TCR sequencing-based approach might be even more sensitive than the *in vitro* AIM assay, at least in detecting antigen-specific CD8 T cells.

Excitingly, the SARS-CoV-2-specific CD4 T cells quantified by the AIM assays were highly correlated with the clonal breadth determined by the TCR-β repertoire sequencing method (Figures 6G and 6H). This correlation did not exist for the quantified SARS-CoV-2-specific CD8 T cells (Figure 6I). It is worth noting that our positive controls (those stimulated by the peptide pool of cytomegalovirus [CMV]) did not show any difference for any of the CD4 or CD8 AIM combinations between HC, MP, and HP (Figures S8A and S8B). No difference in CMV peptide-stimulated samples between different participant groups demonstrated that the observed SARS-CoV-2-specific T cell responses were not due to the unspecific immune responses of the participants within a particular group to a type of common pre-exposed antigen, e.g., CMV.[33] In short, the direct antigen-dependent AIM assays have consolidated our TCR-sequence-inferred results, firmly revealing a dominant early-stage SARS-CoV-2-specific CD4 T cell response among MP. The AIM assays also showed that HP had no deficiency in generating both CD4 and CD8 SARS-CoV-2-specific T cell responses.

(G and H) Clonal breadth of inferred CD4 (G) or CD8 (H) SARS-CoV-2-specific TCR clonotypes.

(I and J) Correlation between inferred CD4 or CD8 SARS-CoV-2-specific TCR clonal breadth and the percentages of $ICOS^+Ki67^+$ cells among CD4 (I) or CD8 T cells (J) in ASP and MP at D1.

ASP, asymptomatic patients, n = 7 or eight; HC, household controls, n = 20; HP, hospitalized patients, n = 2; MP, mild patients, n = 45; D1/D14/D21, day 1/day 14/day 21. Data represent individual values from all biological replicates; mean ± SD. p values from (B–E), (G), and (H) were determined by the Kruskal-Wallis (non-parametric) test and corrected using Dunn's multiple comparisons test. Spearman's correlation coefficient was used in (A), (I), and (J). ns, not significant; $^*p \leq 0.05$, $^{**}p \leq 0.01$, $^{***}p \leq 0.001$.

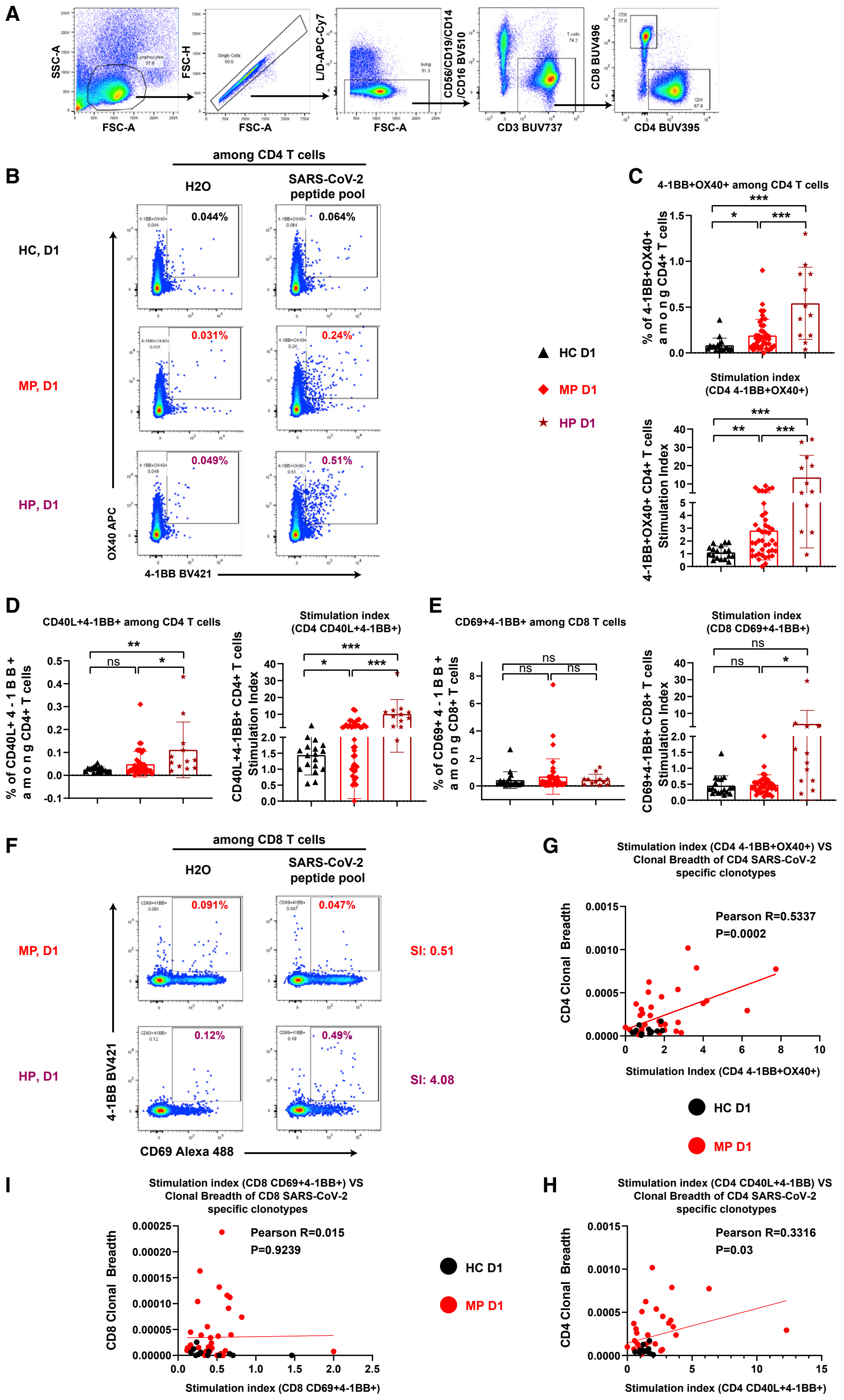


(legend on next page)

## DISCUSSION

In May 2020, a mass PCR screening program was implemented on a population-wide level in Luxembourg,[34] which allowed us to obtain unique access to PCR-positive ASP and MP and to prospectively recruit them into our longitudinal study that was initiated simultaneously.[16] This endorsed us to access a rich resource to fully explore and understand all essential facets of the early-stage and dynamic immunological changes following recent SARS-CoV-2 infection in MP, using an unbiased, combinatorial, and prospective approach.

So far, the immune response in COVID-19 patients has only been investigated in a few longitudinal cohort studies.[17,35–38] These studies concentrated on different time windows and usually put their major focus on one or two selected immunological aspects, which makes it challenging to directly compare them with our multi-faceted analysis. In line with another recent longitudinal study from the United Kingdom (UK),[7] we observed enhanced early-stage CD8 T cells and plasmablast responses in MP. In contrast to that report, our current study provides not only information on the number and frequency of a wide spectrum of peripheral immune subsets but also on the functional status of individual immune cell types. We found both early-stage T-bet-dependent and -independent CD8 T cell responses among MP. Also distinct from the UK cohort,[7] we observed robust early-stage responses of CD4 T cells with a profoundly enhanced frequency of type I IFN-dependent T-bet$^+$Ki67$^+$ CD4 T cells. As another unique result of our study, many other innate immune cells expressing all key functional markers were observed to be intact early on in MP versus HC, but substantially compromised in HP. The notion that early coordinated DC and CD4/CD8 T cell responses have indeed a functional consequence in SARS-CoV-2-specific immunity was further supported by the correlation between mature DCs, CD4, and CD8 T cell responses at early stage (D1) with serum Ab responses 3 weeks later (D21), only among ASP/MP but not HP. Consistent with our own observation of early-stage overall CD4 and CD8 T cell responses, we found a substantial expansion of SARS-CoV-2-specific T cells, predominantly of CD4 T cells, as demonstrated by both TCR repertoire sequencing and AIM assays, in MP already at D1. A more dominant SARS-CoV-2-specific CD4 T cell response in MP might be attributable to pre-existing CD4 memory T cells that cross-reacted with SARS-CoV-2.[39,40] Since we observed enhanced DC and coordinated CD4 and CD8 T cell responses very early on among MP, at most 3 days after PCR diagnosis, the concept of bystander CD8 T cell responses[7] might need to be adapted. Our findings suggest that appropriate and highly coordinated early-stage DC and antigen-specific CD4 and CD8 T cell responses are predictive of the evolution of appropriate immune responses in MP individuals at a later stage. In addition, our longitudinal analysis firmly demonstrates that adaptive immunity was not compromised in HP. Since a highly coordinated early-stage adaptive and innate immune response was only observed in MP but not HP, such a connection between the two arms of immunity appears to be one of the key conditions for a long-term favorable outcome in MP. In this context, it is worth highlighting the strength of our simultaneous and combinatorial analysis strategy of the different immunological facets. By comparing various individual immune features in an isolated and one-dimensional way, various groups in our cohort could not be fully distinguished. For example, both MP and HP displayed enhanced T cell and Ab responses, indicating that one cannot separate them based only on those responses. When analyzing innate immunity, although HP showed impaired responses compared with MP, the innate immune parameters were comparable between MP and HC. As a consequence, none of the individual immune factors alone can fully distinguish the three groups in our cohort. Only a two- or even multi-dimensional view that integrates all layers of innate and adaptive immunity allows for such a differentiation of the three groups in one snapshot. There are other established examples in medicine using multi-dimensional analysis, such as the identification of the different naive and memory T cell subsets that can only be fully distinguished by simultaneously analyzing CD45RA/CD45RO and CCR7 in two dimensions.[41]

Another important observation that is very much in line with the coordinated early-stage DC and antigen-specific CD4 and CD8 T cell responses was the strong early induction of the type I interferon IFN-β in MP. Notably, the early rise of IFN-β levels in MP was followed by a decline to normal levels 3 weeks later. However, such a contraction of enhanced IFN-β levels was not seen in HP. Our current results on early temporary induction of IFN-β levels in MP were further confirmed by a strong correlation between the frequency of mature DC, one of the main producers of type I IFN,[42] and the circulating IFN-β levels on D1. More excitingly, the frequency of activated and proliferating CD4 and CD8 subsets among total CD4 or CD8 T cells was highly correlated with IFN-β/IP10 levels in ASP and MP, but not in HP, on D1. The highly synchronized early-stage immune

**Figure 6. Enhanced early-stage SARS-CoV-2-specific CD4 responses in both mild and hospitalized patients**

(A) Gating strategy to identify total CD4 or CD8 T cells.

(B and F) Representative flow-cytometry plots of 4-1BB/CD137 and OX40/CD134 among CD4 T cells (B) or of CD69 and 4-1BB among CD8 T cells (F) following stimulation by SARS-CoV-2 peptide pool or negative control. No plots from HC were displayed, as they were similar to that of MP in (F).

(C and D) Original or normalized percentages of SARS-CoV-2-specific cells among total CD4 T cells quantified by 4-1BB and OX40 double-positive cells (C), or by CD40L (extracellular) and 4-1BB double-positive cells (D).

(E) Original or normalized percentages of SARS-CoV-2-specific cells among total CD8 T cells quantified by CD69 and 4-1BB double-positive cells.

(G and H) Correlation between SI CD4 AIM (4-1BB$^+$OX40$^+$) (G) or CD4 AIM (CD40L$^+$4-1BB$^+$) (H) and clonal breadth of inferred CD4 SARS-CoV-2 T cells using the sequencing approach.

(I) Correlation between SI based on CD8 AIM (CD69$^+$4-1BB$^+$) and clonal breadth of inferred CD8 SARS-CoV-2 T cells.

HC, household controls, n = 18; HP, hospitalized patients, n = 12; MP, mild patients, n = 42; SI, stimulation index. Data represent individual values from all biological replicates; mean ± SD. p values from (C) to (E) were determined by non-paired two-tailed Student's t test. p values from (G) to (I) were determined by Pearson's correlation coefficient. ns, not significant; $^*p \leq 0.05$, $^{**}p \leq 0.01$, $^{***}p \leq 0.001$. See also Figure S8.

responses further correlated with viral load in MP. Such a multi-layered early-stage coordination of responses including multiple factors such as SARS-CoV-2 viral load, DC activation, CD4 and CD8 T cell stimulation, and relevant circulating cytokine (e.g., IFN-β/IP10) levels might decisively build up the basis for a later beneficial outcome in MP. In contrast to our results, a different study[43] has shown that another antiviral IFN, IFN-λ1 (type III), was negatively correlated with viral load in severe patients, which, however, was apparently not sufficient to guarantee a beneficial clinical outcome. The inverted correlation observed by us and others suggests that the regulatory roles of antiviral IFNs might be very different, if not opposite, in MP versus HP. Our observations of an early time-dependent induction of IFN-β in MP are at least partially in line with studies on genetic and autoimmune defects leading to impaired type I IFN responses that were correlated with severe COVID-19.[23,44,45] Those findings have prompted the proposition of early and transient intervention with recombinant type I IFN as a treatment option in severe patients.[46]

Parallel to IFN-β, we observed substantially enhanced early IP10/CXCL10 levels without other signs of systemic inflammation in MP. IP10 showed a dynamic change very similar to that of IFN-β in MP. The key difference in the cytokine and chemokine responses (IFN-β/IP10) observed between MP and HP is their elevation duration rather than the magnitude. IP10, previously known to exclusively bind to CXCR3, has recently been identified as a high-affinity agonist for the anti-inflammatory atypical chemokine scavenger receptor ACKR2/D6.[47,48] Thus, during the coordinated early anti-SARS-CoV-2 immune response in MP, IP10 might play a role in resolving inflammatory responses. The longer-lasting high levels of IP10 are not unique to severe COVID-19 but occur also in other infectious diseases,[49] including SARS.[50] Our observation in HP is in line with a cross-sectional comparison study,[11] where the IP10 level was enhanced in HP versus ASP. Our results of HP were also similar to a longitudinal cytokine-focused observation in HP,[51] although no MP were included in these analyses. Furthermore, a sustained elevation of IP10 was already reported in severe cases,[52] but only a few MP were included. Similar observations have also been reported[38,53] or reviewed elsewhere.[54,55] However, such a temporal early upregulation of IP10 in MP has never been convincingly reported so far.

Another crucial observation of our study was a very-early-stage (i.e., on D1) signature of reduced frequency and functional impairment of innate immune cells, such as ncMono, pDCs, mDCs, and NK cells, in HP versus MP. Both the frequency of ncMono and the expression of their key functional markers were significantly reduced in HP. Notably, although the frequency of mature DCs was induced early on in both MP and HP, their functional subsets among total DCs were substantially reduced only in HP. In addition, similar findings were also made in HP regarding the reduced frequency of several NK subsets, paralleled by a substantial enhancement in the expression of the inhibitory and terminal differentiation marker KLRG1 on NK cells. Thus, our data of impaired innate immune signatures in HP confirm previous findings of impaired innate immunity in severe or critically ill patients.[38,56,57] However, without including MP into the analyses, none of those previous studies has highlighted that critical differences in early-stage innate immune responses actually exist between non-HP (i.e., ASP and MP) and HP. Thus, our results provide strong support for an early-stage impairment of innate immunity being a unique clinical immunological feature in HP.

Since our analysis was only performed in peripheral blood, our observations might be affected by a potential redistribution of immune cell types between blood and inflammatory tissues. According to the work deposited by Kedzierska and colleagues,[58] the respiratory tract, relative to blood, was dominated by infiltrating neutrophils and a higher frequency of intermediate monocytes/macrophages and effector T cells in severe patients. In our study, we observed a lower frequency of ncMono, pDC, mDC, and NK subsets and substantial changes in their critical functional markers in blood of HP versus MP on D1. Therefore, most of our results on the frequency of these immune cell subsets should not be caused by a potential redistribution between blood and infected tissues. Our observation on the expression levels of functional markers is obviously independent of a potential redistribution of immune cells. Our findings about the frequency of ncMono in blood might have to be evaluated with more caution, as monocyte infiltration into the airways has been identified as an important driver of severe COVID-19.[36] In summary, most of the differences we observed between HP and MP are likely not due to a potential redistribution between blood and infected tissues.

Our data demonstrate that only the combination of different immunological facets can simultaneously distinguish MP, HP, and HC. Notably, based on a sufficiently powered sample size (63 MP) and on the prospective longitudinal nature of our study, we discovered the frequency of specific activated/proliferating CD4 and CD8 T cell subsets and mature DCs at an early disease stage as even better predictors of ensuing humoral responses 3 weeks later than early plasmablast responses in MP. This points to a critical role of DC activation in coordinating very early antigen-specific T cell and later Ab responses. The immune signatures identified in our study bear the potential to be extrapolated to predict protective immune responses in vaccinated people early on. In fact, our discoveries are in line with a recent report showing that mRNA vaccination induces rapid abundant antigen-specific CD4 T cell responses in SARS-CoV-2-naive participants following the first dose,[59] which phenocopies our findings regarding natural infection in MP, thus indicating that the discoveries of our study will have a more general impact on understanding and further dissecting SARS-CoV-2-related immunity.

## Limitations of the study

We would like to point out that the unique combination of various early immune response features were only observed in MP rather than in HP and HC. However, so far we only have data supporting that these immunological features were correlated with mild symptoms in those patients. More mechanistic investigations are still required to figure out exactly which of the early immunological factors play a causal protective role in MP. Although this work critically focused on MP with a plausible number of participants (n = 63), the sample size of ASP and HP was moderate (n = 14 and 15). Recruitment of more ASP and

CellPress
OPEN ACCESS

HP might enhance the analysis power of this study in the conclusions related to those groups of patients. Furthermore, we have only analyzed two time points. Although with all the restrictions for obtaining ethical approval for a cohort of mostly non-critically ill patients in the early pandemic (April 2020), we should have sampled at more time points following the infection, thus allowing us to provide a more detailed dynamic analysis of evolving immune responses. Furthermore, for practical and logistical reasons, we could not perform repetitive sampling in the same individuals immediately after infection for a size of ~120 participants recruited through a population-based program. In other human cohorts, this might be possible if the focus would be on a group of selected people who are PCR tested regularly for professional reasons (e.g., sports athletes), which was not within the scope of our current study. Another limitation is the partial comparability of time from symptom onset to inclusion between MP and HP.

In our cohort, we could not observe any distinguishable immune signature in the peripheral blood of ASP throughout all adaptive and innate immunity analyses. Although the sample size was relatively small in our ASP, the most plausible explanation is a more prominent role of a tissue-resident rather than a systemic response, as recently shown in a pediatric cohort.[60]

## STAR★METHODS

Detailed methods are provided in the online version of this paper and include the following:

- KEY RESOURCES TABLE
- RESOURCE AVAILABILITY
  - Lead contact
  - Materials availability
  - Data and code availability
- EXPERIMENTAL MODEL AND SUBJECT DETAILS
  - Human subjects and cohort design
- METHOD DETAILS
  - Blood sampling and PBMC isolation
  - *Ex vivo* multicolour flow-cytometry-based deep immunophenotyping analysis
  - Activation induced marker (AIM) assay
  - Determination of cytokine and chemokine levels by MSD assay
  - Serological detection of IgG against SARS-CoV-2 by MSD assay
  - Determination of neutralization antibody capacity by MSD assay
  - Determination of SARS-CoV-2 viral load by realtime-reverse transcription PCR (rRT-PCR)
  - TCR repertoire analysis and SARS-CoV-2-specific T-cell inference
  - Statistical analysis

## SUPPLEMENTAL INFORMATION

Supplemental information can be found online at https://doi.org/10.1016/j.xcrm.2022.100600.

## ACKNOWLEDGMENTS

We acknowledge the active involvement of all the participants in the Predi-COVID cohort. We are also thankful for the support of the cohort recruitment team from CIEC of LIH and of the IBBL processing and biorepository teams. We also acknowledge Prof. Laetitia Huiart for her efforts in initiating the Predi-COVID cohort and Aurélie Sausy, Sophie Mériaux, and Jean-Yves Servais for their technical help. The Predi-COVID study is supported by the Luxembourg National Research Fund (FNR) (14716273), the Fondation André Losch, and the European Regional Development Fund (FEDER, no. 2018-04-026-21). F.Q.H. was partially supported by FNR AFR-RIKEN bilateral program (TregBAR, 11228353, F.Q.H. and M.O.) and PRIDE programme (11012546/NEXTIMMUNE and 10907093/CRITICS). C.H. was partially supported by the FNR COVID-19/2020-1/14703957/COV-Immun. P.W. acknowledges funding from the European Research Council under the European Union's Horizon 2020 research and innovation program (no. 863664).

## AUTHOR CONTRIBUTIONS

C.M.C. designed and performed the experiments, performed data analysis, and drafted the manuscript. S.C., O.D., F.H., M.K., and W.A. performed parts of experiments. I.E. and A.F. coordinated the recruitment. C.J.S., C.H., G.G., T.A., and K.G. collected samples and performed parts of experiments. A.C. and M.V. collected clinical data, quality control, and data management. C.L.C. and I.M.K. analyzed the TCR repertoire data. A.S., A.D.S., P.W., A.C., F.B., and G.F. provided substantial supervision and insights into the project. F.Q.H. and M.O. conceived and oversaw the whole project and finalized the manuscript.

## DECLARATION OF INTERESTS

The authors declare no competing interests.

## INCLUSION AND DIVERSITY

We worked to ensure gender balance in the recruitment of human subjects. We worked to ensure ethnic or other types of diversity in the recruitment of human subjects. We worked to ensure that the study questionnaires were prepared in an inclusive way. The author list of this article includes contributors from the location where the research was conducted who participated in the data collection, design, analysis, and/or interpretation of the work.

Received: September 25, 2021
Revised: February 18, 2022
Accepted: March 18, 2022
Published: March 29, 2022

## REFERENCES

1. Kuri-Cervantes, L., Pampena, M.B., Meng, W., Rosenfeld, A.M., Ittner, C.A.G., Weisman, A.R., Agyekum, R.S., Mathew, D., Baxter, A.E., Vella, L.A., et al. (2020). Comprehensive mapping of immune perturbations associated with severe COVID-19. Sci. Immunol. *5*, eabd7114. https://doi.org/10.1126/sciimmunol.abd7114.
2. Mathew, D., Giles, J.R., Baxter, A.E., Oldridge, D.A., Greenplate, A.R., Wu, J.E., Alanio, C., Kuri-Cervantes, L., Pampena, M.B., D'Andrea, K., et al. (2020). Deep immune profiling of COVID-19 patients reveals distinct immunotypes with therapeutic implications. Science *369*, eabc8511. https://doi.org/10.1126/science.abc8511.
3. Arunachalam, P.S., Wimmers, F., Mok, C.K.P., Perera, R.A.P.M., Scott, M., Hagan, T., Sigal, N., Feng, Y., Bristow, L., Tak-Yin Tsang, O., et al. (2020). Systems biological assessment of immunity to mild versus severe COVID-19 infection in humans. Science *369*, 1210–1220. https://doi.org/10.1126/science.abc6261.
4. De Biasi, S., Meschiari, M., Gibellini, L., Bellinazzi, C., Borella, R., Fidanza, L., Gozzi, L., Iannone, A., Lo Tartaro, D., Mattioli, M., et al. (2020). Marked

T cell activation, senescence, exhaustion and skewing towards TH17 in patients with COVID-19 pneumonia. Nat. Commun. *11*, 3434. https://doi.org/10.1038/s41467-020-17292-4.

5. Zheng, M., Gao, Y., Wang, G., Song, G., Liu, S., Sun, D., Xu, Y., and Tian, Z. (2020). Functional exhaustion of antiviral lymphocytes in COVID-19 patients. Cell Mol. Immunol. *17*, 533–535. https://doi.org/10.1038/s41423-020-0402-2.
6. Bacher, P., Rosati, E., Esser, D., Martini, G.R., Saggau, C., Schiminsky, E., Dargvainiene, J., Schroder, I., Wieters, I., Khodamoradi, Y., et al. (2020). Low-avidity CD4(+) T cell responses to SARS-CoV-2 in unexposed individuals and humans with severe COVID-19. Immunity *53*, 1258–1271.e5. https://doi.org/10.1016/j.immuni.2020.11.016.
7. Bergamaschi, L., Mescia, F., Turner, L., Hanson, A.L., Kotagiri, P., Dunmore, B.J., Ruffieux, H., De Sa, A., Huhn, O., Morgan, M.D., et al. (2021). Longitudinal analysis reveals that delayed bystander CD8+ T cell activation and early immune pathology distinguish severe COVID-19 from mild disease. Immunity *54*, 1257–1275.e8. https://doi.org/10.1016/j.immuni.2021.05.010.
8. Wilk, A.J., Rustagi, A., Zhao, N.Q., Roque, J., Martinez-Colon, G.J., McKechnie, J.L., Ivison, G.T., Ranganath, T., Vergara, R., Hollis, T., et al. (2020). A single-cell atlas of the peripheral immune response in patients with severe COVID-19. Nat. Med. *26*, 1070–1076. https://doi.org/10.1038/s41591-020-0944-y.
9. Saichi, M., Ladjemi, M.Z., Korniotis, S., Rousseau, C., Ait Hamou, Z., Massenet-Regad, L., Amblard, E., Noel, F., Marie, Y., Bouteiller, D., et al. (2021). Single-cell RNA sequencing of blood antigen-presenting cells in severe COVID-19 reveals multi-process defects in antiviral immunity. Nat. Cell Biol. *23*, 538–551. https://doi.org/10.1038/s41556-021-00681-2.
10. Ren, X., Wen, W., Fan, X., Hou, W., Su, B., Cai, P., Li, J., Liu, Y., Tang, F., Zhang, F., et al. (2021). COVID-19 immune features revealed by a large-scale single-cell transcriptome atlas. Cell *184*, 1895–1913.e19. https://doi.org/10.1016/j.cell.2021.01.053.
11. Long, Q.-X., Tang, X.-J., Shi, Q.-L., Li, Q., Deng, H.-J., Yuan, J., Hu, J.-L., Xu, W., Zhang, Y., Lv, F.-J., et al. (2020). Clinical and immunological assessment of asymptomatic SARS-CoV-2 infections. Nat. Med. *26*, 1200–1204. https://doi.org/10.1038/s41591-020-0965-6.
12. Sekine, T., Perez-Potti, A., Rivera-Ballesteros, O., Strålin, K., Gorin, J.-B., Olsson, A., Llewellyn-Lacey, S., Kamal, H., Bogdanovic, G., Muschiol, S., et al. (2020). Robust T cell immunity in convalescent individuals with asymptomatic or mild COVID-19. Cell *183*, 158–168.e14. https://doi.org/10.1016/j.cell.2020.08.017.
13. Le Bert, N., Tan, A.T., Kunasegaran, K., Tham, C.Y.L., Hafezi, M., Chia, A., Chng, M.H.Y., Lin, M., Tan, N., Linster, M., et al. (2020). SARS-CoV-2-specific T cell immunity in cases of COVID-19 and SARS, and uninfected controls. Nature *584*, 457–462. https://doi.org/10.1038/s41586-020-2550-z.
14. Delhalle, S., Bode, S.F.N., Balling, R., Ollert, M., and He, F.Q. (2018). A roadmap towards personalized immunology. npj Syst. Biol. Appl. *4*, 9. https://doi.org/10.1038/s41540-017-0045-9.
15. Davis, M.M., Tato, C.M., and Furman, D. (2017). Systems immunology: just getting started. Nat. Immunol. *18*, 725–732. https://doi.org/10.1038/ni.3768.
16. Fagherazzi, G., Fischer, A., Betsou, F., Vaillant, M., Ernens, I., Masi, S., Mossong, J., Staub, T., Brault, D., Bahlawane, C., et al. (2020). Protocol for a prospective, longitudinal cohort of people with COVID-19 and their household members to study factors associated with disease severity: the Predi-COVID study. BMJ Open *10*, e041834. https://doi.org/10.1136/bmjopen-2020-041834.
17. Tan, L., Wang, Q., Zhang, D., Ding, J., Huang, Q., Tang, Y.-Q., Wang, Q., and Miao, H. (2020). Lymphopenia predicts disease severity of COVID-19: a descriptive and predictive study. Signal Transduction Targeted Ther. *5*, 33. https://doi.org/10.1038/s41392-020-0148-4.
18. Wajnberg, A., Amanat, F., Firpo, A., Altman, D.R., Bailey, M.J., Mansour, M., McMahon, M., Meade, P., Mendu, D.R., Muellers, K., et al. (2020). Robust neutralizing antibodies to SARS-CoV-2 infection persist for months. Science *370*, 1227–1230. https://doi.org/10.1126/science.abd7728.
19. Khoury, D.S., Cromer, D., Reynaldi, A., Schlub, T.E., Wheatley, A.K., Juno, J.A., Subbarao, K., Kent, S.J., Triccas, J.A., and Davenport, M.P. (2021). Neutralizing antibody levels are highly predictive of immune protection from symptomatic SARS-CoV-2 infection. Nat. Med. *27*, 1205–1211. https://doi.org/10.1038/s41591-021-01377-8.
20. Rha, M.S., Jeong, H.W., Ko, J.H., Choi, S.J., Seo, I.H., Lee, J.S., Sa, M., Kim, A.R., Joo, E.J., Ahn, J.Y., et al. (2021). PD-1-Expressing SARS-CoV-2-specific CD8(+) T cells are not exhausted, but functional in patients with COVID-19. Immunity *54*, 44–52.e3. https://doi.org/10.1016/j.immuni.2020.12.002.
21. Picozza, M., Battistini, L., and Borsellino, G. (2013). Mononuclear phagocytes and marker modulation: when CD16 disappears, CD38 takes the stage. Blood *122*, 456–457. https://doi.org/10.1182/blood-2013-05-500058.
22. Villasenor-Cardoso, M.I., Frausto-Del-Rio, D.A., and Ortega, E. (2013). Aminopeptidase N (CD13) is involved in phagocytic processes in human dendritic cells and macrophages. Biomed. Res. Int. *2013*, 562984. https://doi.org/10.1155/2013/562984.
23. Hadjadj, J., Yatim, N., Barnabei, L., Corneau, A., Boussier, J., Smith, N., Pere, H., Charbit, B., Bondet, V., Chenevier-Gobeaux, C., et al. (2020). Impaired type I interferon activity and inflammatory responses in severe COVID-19 patients. Science *369*, 718–724. https://doi.org/10.1126/science.abc6027.
24. Naylor, K., Li, G., Vallejo, A.N., Lee, W.W., Koetz, K., Bryl, E., Witkowski, J., Fulbright, J., Weyand, C.M., and Goronzy, J.J. (2005). The influence of age on T cell generation and TCR diversity. J. Immunol. *174*, 7446–7452. https://doi.org/10.4049/jimmunol.174.11.7446.
25. Yager, E.J., Ahmed, M., Lanzer, K., Randall, T.D., Woodland, D.L., and Blackman, M.A. (2008). Age-associated decline in T cell repertoire diversity leads to holes in the repertoire and impaired immunity to influenza virus. J. Exp. Med. *205*, 711–723. https://doi.org/10.1084/jem.20071140.
26. Chen, Z., and John Wherry, E. (2020). T cell responses in patients with COVID-19. Nat. Rev. Immunol. *20*, 529–536. https://doi.org/10.1038/s41577-020-0402-6.
27. Gittelman, R.M., Lavezzo, E., Snyder, T.M., Zahid, H.J., Elyanow, R., Dalai, S., Kirsch, I., Baldo, L., Manuto, L., Franchin, E., et al. (2021). Diagnosis and tracking of SARS-CoV-2 infection by T-cell receptor sequencing. Preprint at medRxiv. https://doi.org/10.1101/2020.11.09.20228023.
28. Snyder, T.M., Gittelman, R.M., Klinger, M., May, D.H., Osborne, E.J., Taniguchi, R., Zahid, H.J., Kaplan, I.M., Dines, J.N., Noakes, M.T., et al. (2020). Magnitude and dynamics of the T-cell response to SARS-CoV-2 infection at both individual and population levels. Preprint at medRxiv. https://doi.org/10.1101/2020.07.31.20165647.
29. Grifoni, A., Weiskopf, D., Ramirez, S.I., Mateus, J., Dan, J.M., Moderbacher, C.R., Rawlings, S.A., Sutherland, A., Premkumar, L., Jadi, R.S., et al. (2020). Targets of T Cell responses to SARS-CoV-2 Coronavirus in humans with COVID-19 disease and unexposed individuals. Cell *181*, 1489–1501.e15. https://doi.org/10.1016/j.cell.2020.05.015.
30. Rydyznski Moderbacher, C., Ramirez, S.I., Dan, J.M., Grifoni, A., Hastie, K.M., Weiskopf, D., Belanger, S., Abbott, R.K., Kim, C., Choi, J., et al. (2020). Antigen-specific adaptive immunity to SARS-CoV-2 in acute COVID-19 and associations with age and disease severity. Cell *183*, 996–1012.e19. https://doi.org/10.1016/j.cell.2020.09.038.
31. Braun, J., Loyal, L., Frentsch, M., Wendisch, D., Georg, P., Kurth, F., Hippenstiel, S., Dingeldey, M., Kruse, B., Fauchere, F., et al. (2020). SARS-CoV-2-reactive T cells in healthy donors and patients with COVID-19. Nature *587*, 270–274. https://doi.org/10.1038/s41586-020-2598-9.
32. Jung, J.H., Rha, M.-S., Sa, M., Choi, H.K., Jeon, J.H., Seok, H., Park, D.W., Park, S.-H., Jeong, H.W., Choi, W.S., and Shin, E.-C. (2021). SARS-CoV-2-specific T cell memory is sustained in COVID-19 convalescent patients for 10 months with successful development of stem cell-like memory

CellPress
OPEN ACCESS

T cells. Nat. Commun. *12*, 4043. https://doi.org/10.1038/s41467-021-24377-1.

33. Lachmann, R., Loenenbach, A., Waterboer, T., Brenner, N., Pawlita, M., Michel, A., Thamm, M., Poethko-Muller, C., Wichmann, O., and Wiese-Posselt, M. (2018). Cytomegalovirus (CMV) seroprevalence in the adult population of Germany. PLoS One *13*, e0200267. https://doi.org/10.1371/journal.pone.0200267.
34. Wilmes, P., Zimmer, J., Schulz, J., Glod, F., Veiber, L., Mombaerts, L., Rodrigues, B., Aalto, A., Pastore, J., Snoeck, C.J., et al. (2021). SARS-CoV-2 transmission risk from asymptomatic carriers: results from a mass screening programme in Luxembourg. The Lancet Reg. Health - Europe *4*, 100056. https://doi.org/10.1016/j.lanepe.2021.100056.
35. Minervina, A.A., Komech, E.A., Titov, A., Bensouda Koraichi, M., Rosati, E., Mamedov, I.Z., Franke, A., Efimov, G.A., Chudakov, D.M., Mora, T., et al. (2021). Longitudinal high-throughput TCR repertoire profiling reveals the dynamics of T-cell memory formation after mild COVID-19 infection. eLife *10*, e63502. https://doi.org/10.7554/eLife.63502.
36. Szabo, P.A., Dogra, P., Gray, J.I., Wells, S.B., Connors, T.J., Weisberg, S.P., Krupska, I., Matsumoto, R., Poon, M.M.L., Idzikowski, E., et al. (2021). Longitudinal profiling of respiratory and systemic immune responses reveals myeloid cell-driven lung inflammation in severe COVID-19. Immunity *54*, 797–814.e6. https://doi.org/10.1016/j.immuni.2021.03.005.
37. Filbin, M.R., Mehta, A., Schneider, A.M., Kays, K.R., Guess, J.R., Gentili, M., Fenyves, B.G., Charland, N.C., Gonye, A.L.K., Gushterova, I., et al. (2021). Longitudinal proteomic analysis of severe COVID-19 reveals survival-associated signatures, tissue-specific cell death, and cell-cell interactions. Cell Rep. Med. *2*, 100287. https://doi.org/10.1016/j.xcrm.2021.100287.
38. Liu, C., Martins, A.J., Lau, W.W., Rachmaninoff, N., Chen, J., Imberti, L., Mostaghimi, D., Fink, D.L., Burbelo, P.D., Dobbs, K., et al. (2021). Time-resolved systems immunology reveals a late juncture linked to fatal COVID-19. Cell *184*, 1836–1857.e22. https://doi.org/10.1016/j.cell.2021.02.018.
39. Mateus, J., Grifoni, A., Tarke, A., Sidney, J., Ramirez, S.I., Dan, J.M., Burger, Z.C., Rawlings, S.A., Smith, D.M., Phillips, E., et al. (2020). Selective and cross-reactive SARS-CoV-2 T cell epitopes in unexposed humans. Science *370*, 89–94. https://doi.org/10.1126/science.abd3871.
40. Habel, J.R., Nguyen, T.H.O., van de Sandt, C.E., Juno, J.A., Chaurasia, P., Wragg, K., Koutsakos, M., Hensen, L., Jia, X., Chua, B., et al. (2020). Suboptimal SARS-CoV-2-specific CD8(+) T cell response associated with the prominent HLA-A*02:01 phenotype. Proc. Natl. Acad. Sci. U S A. *117*, 24384–24391. https://doi.org/10.1073/pnas.2015486117.
41. Sallusto, F., Geginat, J., and Lanzavecchia, A. (2004). Central memory and effector memory T cell subsets: function, generation, and maintenance. Annu. Rev. Immunol. *22*, 745–763. https://doi.org/10.1146/annurev.immunol.22.012703.104702.
42. Asselin-Paturel, C., and Trinchieri, G. (2005). Production of type I interferons: plasmacytoid dendritic cells and beyond. J. Exp. Med. *202*, 461–465. https://doi.org/10.1084/jem.20051395.
43. Galani, I.-E., Rovina, N., Lampropoulou, V., Triantafyllia, V., Manioudaki, M., Pavlos, E., Koukaki, E., Fragkou, P.C., Panou, V., Rapti, V., et al. (2021). Untuned antiviral immunity in COVID-19 revealed by temporal type I/III interferon patterns and flu comparison. Nat. Immunol. *22*, 32–40. https://doi.org/10.1038/s41590-020-00840-x.
44. Bastard, P., Rosen, L.B., Zhang, Q., Michailidis, E., Hoffmann, H.-H., Zhang, Y., Dorgham, K., Philippot, Q., Rosain, J., Béziat, V., et al. (2020). Autoantibodies against type I IFNs in patients with life-threatening COVID-19. Science *370*, eabd4585. https://doi.org/10.1126/science.abd4585.
45. Acharya, D., Liu, G., and Gack, M.U. (2020). Dysregulation of type I interferon responses in COVID-19. Nat. Rev. Immunol. *20*, 397–398. https://doi.org/10.1038/s41577-020-0346-x.
46. Vinh, D.C., Abel, L., Bastard, P., Cheng, M.P., Condino-Neto, A., Gregersen, P.K., Haerynck, F., Cicalese, M.P., Hagin, D., Soler-Palacin, P., et al. (2021). Harnessing type I IFN immunity against SARS-CoV-2 with early administration of IFN-beta. J. Clin. Immunol. *41*, 1425–1442. https://doi.org/10.1007/s10875-021-01068-6.
47. Jamieson, T., Cook, D.N., Nibbs, R.J.B., Rot, A., Nixon, C., McLean, P., Alcami, A., Lira, S.A., Wiekowski, M., and Graham, G.J. (2005). The chemokine receptor D6 limits the inflammatory response in vivo. Nat. Immunol. *6*, 403–411. https://doi.org/10.1038/ni1182.
48. Chevigné, A., Janji, B., Meyrath, M., Reynders, N., D'Uonnolo, G., Uchański, T., Xiao, M., Berchem, G., Ollert, M., Kwon, Y.-J., et al. (2021). CXCL10 is an agonist of the CC family chemokine scavenger receptor ACKR2/D6. Cancers *13*, 1054.
49. Liu, M., Guo, S., Hibbert, J.M., Jain, V., Singh, N., Wilson, N.O., and Stiles, J.K. (2011). CXCL10/IP-10 in infectious diseases pathogenesis and potential therapeutic implications. Cytokine Growth Factor Rev. *22*, 121–130. https://doi.org/10.1016/j.cytogfr.2011.06.001.
50. Wong, C.K., Lam, C.W., Wu, A.K., Ip, W.K., Lee, N.L., Chan, I.H., Lit, L.C., Hui, D.S., Chan, M.H., Chung, S.S., and Sung, J.J. (2004). Plasma inflammatory cytokines and chemokines in severe acute respiratory syndrome. Clin. Exp. Immunol. *136*, 95–103. https://doi.org/10.1111/j.1365-2249.2004.02415.x.
51. Yang, Y., Shen, C., Li, J., Yuan, J., Wei, J., Huang, F., Wang, F., Li, G., Li, Y., Xing, L., et al. (2020). Plasma IP-10 and MCP-3 levels are highly associated with disease severity and predict the progression of COVID-19. J. Allergy Clin. Immunol. *146*, 119–127.e4. https://doi.org/10.1016/j.jaci.2020.04.027.
52. Laing, A.G., Lorenc, A., del Molino del Barrio, I., Das, A., Fish, M., Monin, L., Muñoz-Ruiz, M., McKenzie, D.R., Hayday, T.S., Francos-Quijorna, I., et al. (2020). A dynamic COVID-19 immune signature includes associations with poor prognosis. Nat. Med. *26*, 1623–1635. https://doi.org/10.1038/s41591-020-1038-6.
53. Liu, Y., Zhang, C., Huang, F., Yang, Y., Wang, F., Yuan, J., Zhang, Z., Qin, Y., Li, X., Zhao, D., et al. (2020). Elevated plasma levels of selective cytokines in COVID-19 patients reflect viral load and lung injury. Natl. Sci. Rev. *7*, 1003–1011. https://doi.org/10.1093/nsr/nwaa037.
54. Vabret, N., Britton, G.J., Gruber, C., Hegde, S., Kim, J., Kuksin, M., Levantovsky, R., Malle, L., Moreira, A., Park, M.D., et al. (2020). Immunology of COVID-19: current state of the science. Immunity *52*, 910–941. https://doi.org/10.1016/j.immuni.2020.05.002.
55. Mather, M.W., Jardine, L., Talks, B., Gardner, L., and Haniffa, M. (2021). Complexity of immune responses in COVID-19. Semin. Immunol. *55*, 101545. https://doi.org/10.1016/j.smim.2021.101545.
56. Blanco-Melo, D., Nilsson-Payant, B.E., Liu, W.C., Uhl, S., Hoagland, D., Moller, R., Jordan, T.X., Oishi, K., Panis, M., Sachs, D., et al. (2020). Imbalanced host response to SARS-CoV-2 drives development of COVID-19. Cell *181*, 1036–1045.e9. https://doi.org/10.1016/j.cell.2020.04.026.
57. Peyneau, M., Granger, V., Wicky, P.-H., Khelifi-Touhami, D., Timsit, J.-F., Lescure, F.-X., Yazdanpanah, Y., Tran-Dihn, A., Montravers, P., Monteiro, R.C., et al. (2021). Innate immune deficiencies in patients with COVID-19. Preprint at medRxiv. https://doi.org/10.1101/2021.03.29.21254560.
58. Zhang, W., Chua, B.Y., Selva, K.J., Kedzierski, L., Ashhurst, T.M., Haycroft, E.R., Shoffner, S.K., Hensen, L., Boyd, D.F., James, F., et al. (2021). Immune responses in COVID-19 respiratory tract and blood reveal mechanisms of disease severity. Preprint at medRxiv. https://doi.org/10.1101/2021.09.01.21262715.
59. Painter, M.M., Mathew, D., Goel, R.R., Apostolidis, S.A., Pattekar, A., Kuthuru, O., Baxter, A.E., Herati, R.S., Oldridge, D.A., Gouma, S., et al. (2021). Rapid induction of antigen-specific CD4+ T cells is associated with coordinated humoral and cellular immune responses to SARS-CoV-2 mRNA vaccination. Immunity *54*, 2133–2142.e3. https://doi.org/10.1016/j.immuni.2021.08.001.

60. Loske, J., Röhmel, J., Lukassen, S., Stricker, S., Magalhães, V.G., Liebig, J., Chua, R.L., Thürmann, L., Messingschlager, M., Seegebarth, A., et al. (2021). Pre-activated antiviral innate immunity in the upper airways controls early SARS-CoV-2 infection in children. Nat. Biotechnol. *40*, 319–324. https://doi.org/10.1038/s41587-021-01037-9.

61. Capelle, C.M., Ciré, S., Ammerlaan, W., Konstantinou, M., Balling, R., Betsou, F., Cosma, A., Ollert, M., and Hefeng, F.Q. (2021). Standard peripheral blood mononuclear cell cryopreservation selectively decreases detection of nine clinically relevant T cell markers. ImmunoHorizons *5*, 711–720. https://doi.org/10.4049/immunohorizons.2100049.

62. Robins, H.S., Campregher, P.V., Srivastava, S.K., Wacher, A., Turtle, C.J., Kahsai, O., Riddell, S.R., Warren, E.H., and Carlson, C.S. (2009). Comprehensive assessment of T-cell receptor beta-chain diversity in alphabeta T cells. Blood *114*, 4099–4107. https://doi.org/10.1182/blood-2009-04-217604.

63. Klinger, M., Pepin, F., Wilkins, J., Asbury, T., Wittkop, T., Zheng, J., Moorhead, M., and Faham, M. (2015). Multiplex identification of antigen-specific T cell receptors using a combination of immune assays and immune receptor sequencing. PLoS One *10*, e0141561. https://doi.org/10.1371/journal.pone.0141561.

## STAR★METHODS

### KEY RESOURCES TABLE

| REAGENTS or RESOURCE | SOURCE | IDENTIFIER |
|---|---|---|
| Antibodies | | |
| Fc Block | BD | Cat# 564765; RRID:AB_2869612 |
| CD4 BUV395 | BD | Cat# 563550; RRID:AB_2738273 |
| CD8 BUV496 | BD | Cat# 612942; RRID:AB_2870223 |
| CD3 BUV737 | BD | Cat# 741822; RRID:AB_2871157 |
| CD56 BV510 | BD | Cat# 740171; RRID:AB_2739924 |
| TIM3 BV786 | BD | Cat# 742857; RRID:AB_2741100 |
| CD57 FITC | BD | Cat# 561906; RRID:AB_10897013 |
| CD16 BB700 | BD | Cat# 746199; RRID:AB_2743545 |
| GZMB PE (intracellular)[§] | BD | Cat# 561142; RRID:AB_10561690 |
| CD45RO PE-CF594 | BD | Cat# 562299; RRID:AB_11154398 |
| CD38 BV510* | BD | Cat# 563251; RRID:AB_2738097 |
| CXCR5 BV711 | BD | Cat# 740737; RRID:AB_2740408 |
| HLA-DR BV786 | BD | Cat# 564041; RRID:AB_2738559 |
| Ki67 AF488 (intracellular) | BD | Cat# 561165; RRID:AB_10611866 |
| CD27 BB700 | BD | Cat# 566449; RRID:AB_2739731 |
| CD40L PE-Cy5 | BD | Cat# 555701; RRID:AB_396051 |
| CD86 BUV395 | BD | Cat# 740305; RRID:AB_2870635 |
| IgD BUV 496 | BD | Cat# 741170; RRID:AB_2870742 |
| CD14 BUV737 | BD | Cat# 612763; RRID:AB_2870094 |
| CD123 BV421 | BD | Cat# 563362; RRID:AB_2738158 |
| CD3 BV510 | BD | Cat# 564713; RRID:AB_2738909 |
| CD40 BV605 | BD | Cat# 740410; RRID:AB_2740140 |
| CD13 BV711 | BD | Cat# 740772; RRID:AB_2740435 |
| PD-L1 BB515 | BD | Cat# 564554; RRID:AB_2738842 |
| CD19 BB700 | BD | Cat# 566411; RRID:AB_2744315 |
| CD38 PE | BD | Cat# 560981; RRID:AB_10563932 |
| CD11c PE-CF594 | BD | Cat# 562393; RRID:AB_11153662 |
| CD80 PE-CY7 | BD | Cat# 561135; RRID:AB_10561688 |
| CD27 APC | BD | Cat# 561786; RRID:AB_10896653 |
| ICOS (CD278) BV605 | BioLegend | Cat# 313538; RRID:AB_2687079 |
| KLRG1 PE-Cy7 | BioLegend | Cat# 368614; RRID:AB_2728371 |
| FOXP3 APC (intracellular) | BioLegend | Cat# 320114; RRID:AB_439754 |
| CCR7 (CD197) BV421 | BioLegend | Cat# 353208; RRID:AB_11203894 |
| CD45RA BV421 | BioLegend | Cat# 304130; RRID:AB_10965547 |
| PD-1 (CD279) BV605 | BioLegend | Cat# 329924; RRID:AB_2563212 |
| CD127 BV711 | BioLegend | Cat# 351328; RRID:AB_2562908 |
| T-bet PE (intracellular) | BioLegend | Cat# 644810; RRID:AB_2200542 |
| CD194 APC | BioLegend | Cat# 359408; RRID:AB_2562429 |
| EOMES PE-Cy7 (intracellular) | Thermo Fischer Scientific | Cat# 25-4877-42; RRID:AB_2573456 |
| Abs above used for deep immunophenotypic analysis | | N/A |
| CD4 BUV395 (from this ab below used for AIM assay) | BD | Cat# 563550; RRID:AB_2738273 |

(*Continued on next page*)

| *Continued* | | |
|---|---|---|
| REAGENTS or RESOURCE | SOURCE | IDENTIFIER |
| CD8 BUV496 | BD | Cat# 612942; RRID:AB_2870223 |
| CD3 BUV737 | BD | Cat# 741822; RRID:AB_2871157 |
| 4-1BB (CD137) BV421 | Biolegend | Cat# 309820; RRID:AB_2563830 |
| CD14 BV510 | Biolegend | Cat# 301842; RRID:AB_2561946 |
| CD16 BV510 | Biolegend | Cat# 302048; RRID:AB_2562085 |
| CD19 BV510 | Biolegend | Cat# 302242; RRID:AB_2561668 |
| CD56 BV510 | BD | Cat# 740171; RRID:AB_2739924 |
| CD69 AF488 | Biolegend | Cat# 310916; RRID:AB_528869 |
| CD40L PE-Dazzle594 | Biolegend | Cat# 310840; RRID:AB_2566245 |
| OX40 (CD134) AF647 | Biolegend | Cat# 350018; RRID:AB_2571938 |
| Anti-CD40 antibody | Miltenyi Biotec | Cat# 130-094-133; RRID:AB_10839704 |
| Chemicals, peptides and recombinant proteins | | |
| RPMI | Thermo Fisher Scientific | Cat# 21870084 |
| Human Serum | Merck | Cat#H5667 |
| GlutaMAX | Thermo Fisher Scientific | Cat# 35050061 |
| Penicillin-streptomycin | Thermo Fisher Scientific, | Cat# 15070063 |
| PepTivator® SARS-CoV-2 select | Miltenyi Biotec | Cat# 130-127-309 |
| PepTivator® CMV pp65 (premium grade) | Miltenyi Biotec | Cat# 130-093-438 |
| Brilliant stain buffer | BD | Cat# 563794 |
| Critical commercial assays | | |
| U-Plex Biomarker Group 1 (hu) assay | Meso Scale Diagnostics (MSD) | Cat# K15067L-1 |
| V-Plex COVID-19 Coronavirus Panel1 IgG kit | MSD | Cat# K15362U |
| COVID-19 ACE2 Neutralization Kit | MSD | Cat# K15375U |
| QIAamp Viral RNA Mini Kit | Qiagen | Cat# 52906 |
| TaqPath$^{TM}$ one-Step RT-qPCR Master Mix, CG | Life Technologies | Cat# A15299 |
| EDX SARS-CoV-2 standard | BioRad | Cat# COV019 |
| CryoStor CS10 | Biolife Solutions | Cat# 210102 |
| LIVE/DEAD$^{TM}$ (L/D) Fixable Near-IR (APC-Cy7) Dead Cell Stain Kit | Life Technologies | Cat# L34975 |
| True-Nuclear Transcription Factor Buffer Set | BioLegend | Cat# 424401 |
| Ultracomp Compensation Beads | Thermo Fischer Scientific | Cat# 01-2222-42 |
| Software and algorithms | | |
| FlowJo v10.5.6 | BD | https://www.flowjo.com/solutions/flowjo; RRID:SCR_008520 |
| Graphpad Prism 9.0 | Graphpad Software | https://www.graphpad.com/; RRID:SCR_002798 |
| Others | | |
| $K_2$EDTA | BD | Cat# 367525 |

## RESOURCE AVAILABILITY

### Lead contact

Further information and requests for different resources should be directed to and will be fulfilled by the lead contact, Feng Hefeng, Department of Infection and Immunity, Luxembourg Institute of Health, Esch-sur-Alzette, Luxembourg (feng.he@lih.lu).

### Materials availability

The study did not generate any new unique materials.

### Data and code availability

- All data reported in this work will be shared by the lead contact upon request. The TCR repertoire sequence data and related metadata generated in this work are available via https://doi.org/10.21417/CMC2022CRM.

- This work does not report original code.
- Any additional information required to re-analyze the data reported in this work is available from the lead contact upon request.

## EXPERIMENTAL MODEL AND SUBJECT DETAILS

### Human subjects and cohort design

The Luxembourg National Research Ethics Committee (CNER) has given approval to this study (Predi-COVID) (202003/07). The trial has been registered at ClinicalTrials.gov (NCT04380987). All collections were performed with approval from relevant ethic organizations. Informed consent was obtained from each participant prior to collection. The blood/swab sampling was performed by nurses from Clinical and Epidemiological Investigation Centre (CIEC) of LIH.

Predi-COVID is a prospective longitudinal cohort study composed of individuals older than 18 years of age with a positive PCR test for SARS-CoV-2 in Luxembourg. Blood or swab samples were collected by a nurse at the latest 3 days post clinical PCR diagnosis (baseline, as day 1) at home for asymptomatic and mild participants. The mean time lag from the onset of first symptoms to the inclusion for mild patients was 6 days. For hospitalized patients, except for two of them (sampled 5 or 6 days post hospital arrival), the remaining 13 patients were all sampled at the latest 3 days after hospitalization. The mean time lag from the onset of first symptoms to the inclusion for mild patients was 12 days. A follow-up visit was organized 3 weeks (day 21, D21) later. Please note that the number of participants in the same category might be slightly different between baseline and the follow-up due to either practical or technical issues (refer to Figure 1A). The indicated number in each figure referred to the larger one among the two analyzed time points for each category. The group of mild COVID-19 patients also contained 11 patients with self-reported shortness-of-breath symptoms that could not be confirmed by a physician and therefore could not be classified as moderate patients following the NIH guideline.

The Predi-COVID-H sub-study is a prospective longitudinal cohort study composed of household members of a Predi-COVID participant as controls. Biological samples were collected at the same time as for the Predi-COVID participant sharing the house (baseline, as day 1, D1) and 2 weeks later, at day 14 (D14). The swab samples were collected at day 14 for the household controls. More details on the study design have been described elsewhere.[16]

The time point at day 21 was chosen as the ideal follow-up time point in all COVID-19 patients recruited. Although it would have been ideal to do the follow-up of the household controls at day 21 as well, the ethical committee requested us to perform the follow-up earlier, i.e. after 14 days. The reason for that decision was to capture a potential COVID-19 infection in the household control members early enough and, in such a case, to refer them early to the medical healthcare system.

## METHOD DETAILS

### Blood sampling and PBMC isolation

Samples were collected from confirmed SARS-COV-2 positive patients and household controls by trained nurses from the LIH-CIEC. Blood samples were collected in CAT, $K_2$EDTA and CPT (all from BD, Erembodegem, Belgium) by the standard phlebotomy procedure. Blood samples were transported daily to centralized processing laboratory (IBBL) at ambient temperature.

CAT tubes were centrifuged for 10 min at 2000 x g, room temperature (RT). Serum upper layer was sterile aliquoted and stored at $-80^{\circ}$C. Prior to centrifugation, 200 $\mu$L from the $K_2$EDTA was transferred into 0.5 mL microcentrifuge tube for complete blood count (CBC) on ABX Micros CRP200 (Horiba, Japan). The $K_2$EDTA tube was centrifuged for 20 min at 2000xg, 4°C. Plasma upper layer and buffy-coat were aliquoted and stored at $-80^{\circ}$C. The CPT tubes were centrifuged for 20 min at 1800 x g, RT. The collected PBMCs were washed twice in $Ca^{2+}$ free PBS and counted using a Cellometer (Nexcelom, UK). Fresh PBMCs were partly used for direct flow cytometry and partly cryopreserved in CryoStor CS10 (Biolife solutions, USA) by controlled-rate freezing using Mr. Forsty (Nalgene, USA), followed by a long-term storage in liquid nitrogen.

### *Ex vivo* multicolour flow-cytometry-based deep immunophenotyping analysis

For each panel staining, $1 \times 10^6$ isolated fresh PBMCs, rather than frozen PBMCs, were used since cryopreservation affects several relevant markers as we demonstrated elsewhere.[61] The cells were resuspended in 50 ul of Brilliant stain buffer (BD, 563794) containing 2.5 ul of Fc blocking antibodies (BD, 564765) and incubated for 15 min. The suspension was then mixed with 50 ul of the respective 2x concentrated mastermix for the surface staining. The fluorochromes associated to the different markers are specified in key resources table. After 30 min of incubation in the dark at 4°C, the cells were washed three times with FCM buffer (flow cytometry [FCM] staining buffer, $Ca^{2+}$- and $Mg^{2+}$-free PBS + 2% heat-inactivated FBS) (5 min, 300xg). Following the final washing step, the stained PBMCs were fixed in 200 ul of 4% PFA (ThermoFisher Scientific, 28906) and incubated at RT for 30 min in the dark. After the PFA fixation, the PBMCs were washed once in FCM buffer (5 min, 400 x g) and resuspended in 200 ul fixation buffer from the True-Nuclear Transcription Factor Buffer Set (Biolegend, 424401). After 1 h of incubation in the dark at RT, the cells were centrifuged down (5 min, 400xg) and resuspened in 200 ul FCM buffer and left at 4°C overnight.

In the next morning the cells were resuspended in permeabilisation buffer (Biolegend, 424401) containing 2.5 ul of Fc blocking antibodies (BD, 564765) and incubated for 15 min at RT. After the intracellular blocking step, the cells were resuspended in 100 ul

permeabilisation buffer containing the antibodies for the intracellular staining (key resources table). Of note, different fluorochromes were used for some markers among different panels. After 30 min of incubation at RT in the dark, the cells were washed three times with permeabilisation buffer (5 min, 400 x g) and resuspended in 100 ul of FCM buffer to proceed to the acquisition on a BD LSRFortessa$^{TM}$ analyzer. To ensure a consistent acquisition of all the markers over the whole duration of the study, the application settings of the instrument were saved during the first acquisition and applied to all the following samples of the cohort as a part of our clinical research standard. The data was analyzed using FlowJo v10.5.6.

### Activation induced marker (AIM) assay

The cryopreserved PBMCs were recovered in 10 mL of pre-warmed RPMI medium (Thermo Fisher Scientific, 21870084) [supplemented with 5% heat-inactivated human serum (Merck, H5667), 2 mM GlutaMAX$^{TM}$ (Thermo Fisher Scientific, 35050061), 50 U/mL penicillin and 50 μg streptomycin (Thermo Fisher Scientific, 15070063)] and centrifuged for 10 min, 200xg, RT. They were then resuspended in 2 mL of pre-warmed medium and rested overnight in a 24-well plate at 37°C, 5% CO2. The next day $1 \times 10^6$ of PBMCs per well were seeded in a round-bottom 96-well plate, first blocked for 15 min at 37°C, 5% CO2 with 0.5 ug/mL anti-CD40 antibodies (Miltenyi Biotec, 130-094-133) and then stimulated with 1 ug/mL of the specific peptide pool or H20 for 24 h at 37°C, 5% CO2 following manufacture's recommendation. The peptide pool we used was either PepTivator® SARS-CoV-2 (derived from the whole-virus proteome of wildtype SARS-CoV-2, premium grade, Miltenyi Biotec, 130-127-309) or PepTivator® CMV pp65 (premium grade, Miltenyi Biotec, 130-093-438). Of note, we used H2O rather than DMSO as a negative control because the peptide pools were reconstituted in H2O according to the manufacturer's recommendation. Following stimulation, the cells were stained using the master mixture of surface Abs provided in key resources table. The staining procedure was identical to that described in immunophenotyping section above. Considering the extremely-low frequency of antigen-specific T cells, we acquired up to 1.5E5 of gated lymphocytes for the AIM assay samples on a BD LSRFortessa$^{TM}$ analyzer followed by the analysis using FlowJo v10.5.6. The statistical analysis and visualization was then performed using Graphpad prism 9.0. The stimulation index was calculated as the ratio between the original percentage of the corresponding AIM combination stimulated by the peptide pool and background percentage in the negative control of the same sample. If the background percentage was zero, we used the minimal background values in the given group (household controls, mild or hospitalized patients at the given day) as the corresponding denominator.

### Determination of cytokine and chemokine levels by MSD assay

24 cytokines, chemokines or growth factors (eotaxin-1/CCL11, G-CSF, GM-CSF, IFN-α2a, IFN-β, IFN-γ, IL-1α, IL-1β, IL-4, IL-5, IL-6, IL-8, IL-10, IL12p70, IL-13, IL-33, IP10, MCP-1, MIP-1α, TARC, TNF-α, TNF-β, TSLP, VEGFA) were measured in participants' sera using a multiplex assay [U-Plex Biomarker Group 1 (hu) assay from MSD Kit catalog Number K15067L-1]. The samples were undiluted. The assay was performed according to the manufacturer's instructions. Data were recorded and analyzed on a MESO QuickPlex SQ 120 instrument.

### Serological detection of IgG against SARS-CoV-2 by MSD assay

V-Plex COVID-19 Coronavirus Panel 1 serology kits from MSD (reference K15362U) were used to detect the presence of IgG antibodies to SARS-CoV-2-Spike (S), SARS-CoV-2 Nucleocapsid (N), SARS-CoV-2-S N-terminal domain (NTD) and SARS-CoV-2-S receptor binding domain (RBD) in diluted sera (1/500) according to the manufacturer's instructions. The plate was read on an MSD instrument, which measures the light emitted from the MSD SULFO-TAG. To determine the cutoff values for positivity for SARS-CoV-2, we measured 35 patients in another cohort from Central Hospital of Luxembourg (PCR positive and >15 days symptom onset) and negative sera before the pandemic from 2019 stored in Luxembourg National Laboratory (LNS). Of note, the hospitalized samples (both at day 1 and day 21 of inclusion) in this cohort were measured using the plates with the lot number different from the other groups and the corresponding positive thresholds of those plates were calculated accordingly. To guarantee the comparability of positive percentages between the two batches, the choice of the cutoffs aimed for a similar sensitivity and specificity between the two batches of plates (the AUC analysis was done by GraphPad Prism 9.0). In Figure 1, the statistical test between non-hospitalized groups was based on the signals. Only the positive percentages, rather than the signals of hospitalized samples were directly compared with that of other groups because abs from different lots were used.

### Determination of neutralization antibody capacity by MSD assay

The assay analyzes the capacity of antibodies to inhibit the binding of labelled recombinant ACE2, the human receptor for SARS-CoV-2, to CoV-2 S or CoV-2 RBD in a multiplex high-throughput format. Multiplex assays for the detection of neutralizing antibodies against SARS-CoV-2 (SARS-CoV-2-Spike and SARS-CoV-2 S RBD) were done on patient sera using the MSD COVID-19 ACE2 Neutralization Kits from MSD (Panel 1 reference K15375U) according to the manufacturer's instructions. The samples were diluted 50 times for the neutralization assay. Data were recorded on a MESO QuickPlex SQ 120 instrument, which measures the light emitted from the MSD SULFO-TAG. Results were reported as inhibition percentage calculated using the equation below. % Inhibition was calculated using the following equation: (1- Average Sample ECL Signal/Average ECL signal of calibrator) *100.

### Determination of SARS-CoV-2 viral load by realtime-reverse transcription PCR (rRT-PCR)

Oropharyngeal and nasopharyngeal swabs were collected from SARS-CoV-2 positive patients on day 1 and from household controls on day 14. Both swabs were discharged in a single tube containing Universal Transport Medium (Copan, Italy). Swabs were transported at 4°C to the processing laboratory, where they were homogenized by vortexing for 30 sec. After centrifugation for 1 min at 1000xg, 4°C, supernatant was aliquoted and stored at −80°C until further analysis.

Viral RNA was extracted using the QIAamp Viral RNA Mini Kit (52906, Qiagen, Venlo, the Netherlands) from 140 μL of swab medium according to the manufacturer protocol and eluted in 60 μL of elution buffer. Presence of SARS-CoV-2 viral RNA was assessed independently of initial clinical PCR diagnosis by analyzing the N gene using rRT-PCR (CDC, N1 target). The reaction was carried using TaqPath™ one-Step RT-qPCR Master Mix, CG (A15299, Life Technologies, Merelbeke, Belgium) with a 20 μL of final volume, 0.5 μM final concentration of both primers (Fwd: 5′-GAC CCC AAA ATC AGC GAA AT-3′; Rev: 5′-TCT GGT TAC TGC CAG TTG AAT CTG-3′) and 0.125 μM probe (5′-FAM-ACC CCG CAT TAC GTT TGG TGG ACC-BHQ1-3′) and 5 μL of RNA. Real-time PCR cycling conditions were used as follows: reverse transcription for 15 min at 50°C and 2 min at 95°C followed by 45 PCR cycles at 95°C for 3 sec; 55°C for 30 sec. Human RNase P RNA levels were assessed using similar conditions (annealing/elongation step at 58°C; Fwd primer 5′-AGA TTT GGA CCT GCG AGC G-3′; Rev primer: 5′-GAG CGG CTG TCT CCA CAA GT-3′; Probe: 5′-FAM- TTC TGA CCT GAA GGC TCT GCG CG-BHQ1-3′, CDC) to monitor sample quality and the RNA extraction process. All PCRs were carried on CFX96 Touch real time instruments and results analyzed with the CFX Maestro software (BioRad, Temse, Belgium).

To quantify the viral load in swabs, RNA extracts were tested in duplicates with the rRT-PCR protocol described above, together with a 3-fold dilution curve of the EDX SARS-CoV-2 standard (COV019, BioRad) tested in triplicate on each PCR plate. Samples for which viral RNA concentration exceeded the upper range of the dilution curve were further retested in duplicates after dilutions of RNA extracts.

### TCR repertoire analysis and SARS-CoV-2-specific T-cell inference

Immunosequencing of the CDR3 regions of human TCRβ chains was performed using the ImmunoSEQ® Assay (Adaptive Biotechnologies, Seattle, WA). Extracted genomic DNA from ~5 × e6 cryopreserved PBMCs was amplified in a bias-controlled multiplex PCR, followed by high-throughput sequencing. Sequences were collapsed and filtered in order to identify and quantitate the absolute abundance of each unique TCRβ CDR3 region for further analysis as previously described.[62]

Clonality was defined as 1- normalized Shannon's Entropy and was calculated on productive rearrangements. Clonality values approaching 0 indicate a very even distribution of frequencies, whereas values approaching 1 indicate an increasingly asymmetric distribution in which a few clones are present at high frequencies. Clonal breadth and depth of SARS-CoV2-associated TCRβ sequences were calculated as previously described,[27] using a set of sequences described elsewhere.[28] Briefly, breadth is calculated as the proportion of unique annotated SARS-CoV-2 specific rearrangements out of the total number of unique productive rearrangements, while depth accounts for the extent of the expansion of those clonal lineages in the repertoire.

We inferred whether TCR sequences are CD4 or CD8 T-cells by associating each sequence to a Class II or Class I HLA using a logistic regression classifier with L1 regularization in an independent dataset. The HLA class associations were validated using data from independent multiplexed antigen-specific T-cell receptor stimulation assay (MIRA) experiments[63] in which the sequence was directly observed in a Class I or Class II experiment. We find ~90% consistency between associations based on MIRA and our inferred sequence-HLA associations. Results from these models were used to infer CD4 or CD8 labels for the sequences from the current study with high confidence. Clonal breadth and depth for these labeled sequences were estimated as described above.

### Statistical analysis

Both PCA and volcano plots were visualized using GraphPad Prism 9.0. Correlation analysis was based on either Spearman or Pearson correlation as indicated in the corresponding figures. We only displayed the top-ranked highly correlated results if the correlation coefficient was ranked in the top positions among 484 correlations, calculated between antibody levels, cytokines or TCR breadth and any of the 484 subsets. The corresponding P-values from correlation analysis were based on a two-tailed analysis using GraphPad Prism 9.0. P-value from each scatter dot plots was determined by the Kruskal-Wallis (nonparametric) test and corrected using the Dunn's multiple comparisons test from GraphPad Prism 9.0. P-value in the AIM assay was calculated by non-paired Student t test. In addition to each individual value measured from each participant, data from each group were presented as mean ± standard deviation (S.D.). ns, not significant, *p<=0.05, **p<=0.01 and ***p<=0.001.

Cell Reports Medicine, Volume *3*

## Supplemental information

# Combinatorial analysis reveals highly coordinated early-stage immune reactions that predict later antiviral immunity in mild COVID-19 patients

**Christophe M. Capelle, Séverine Ciré, Olivia Domingues, Isabelle Ernens, Fanny Hedin, Aurélie Fischer, Chantal J. Snoeck, Wim Ammerlaan, Maria Konstantinou, Kamil Grzyb, Alexander Skupin, Cara L. Carty, Christiane Hilger, Georges Gilson, Aljosa Celebic, Paul Wilmes, Antonio Del Sol, Ian M. Kaplan, Fay Betsou, Tamir Abdelrahman, Antonio Cosma, Michel Vaillant, Guy Fagherazzi, Markus Ollert, and Feng Q. Hefeng**

## Supplemental Information

## This file includes:

**Figure S1-S8**

**Table S1**

## Supplementary Figures:

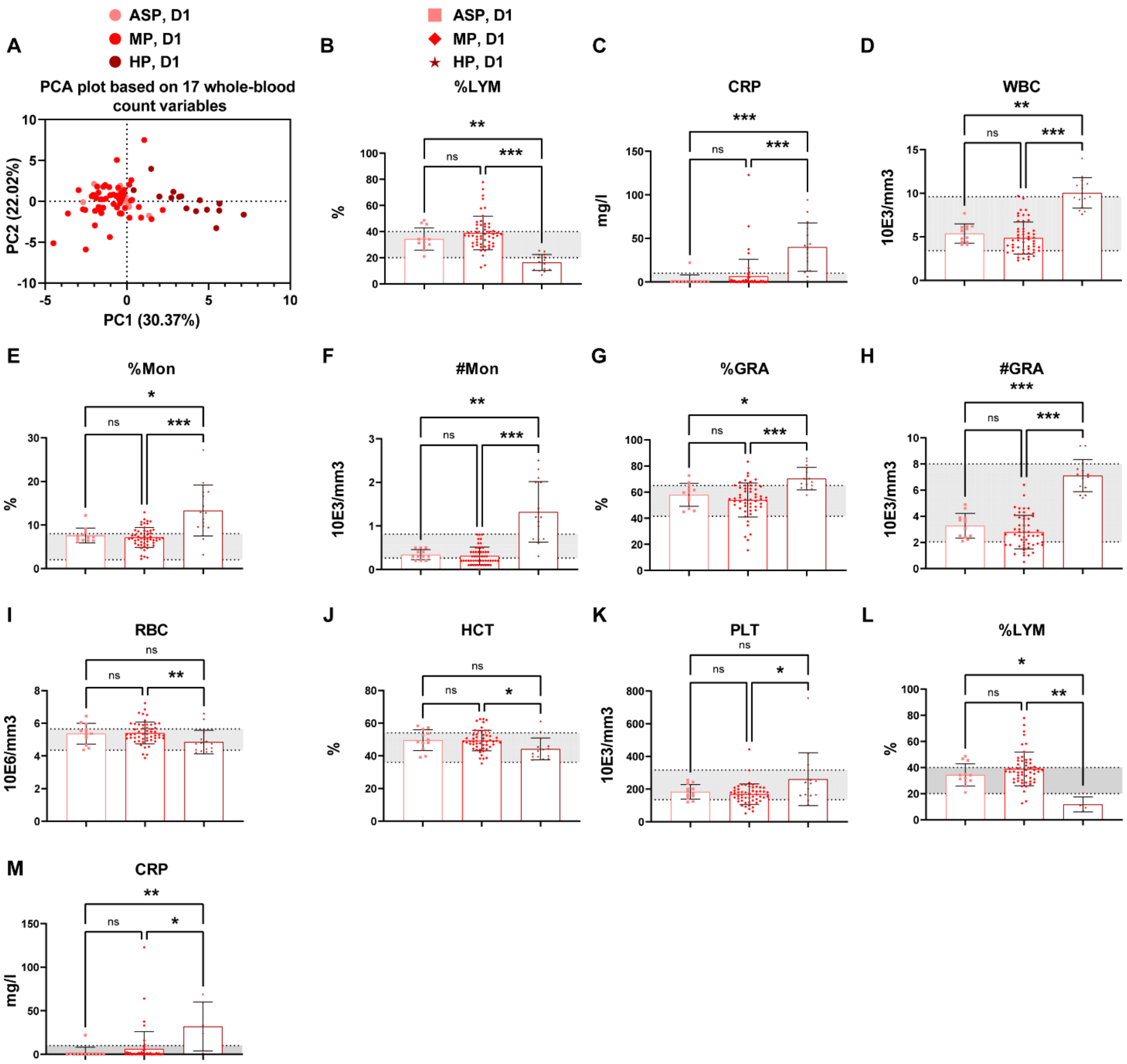


**Figure S1. Whole-blood-count analysis of asymptomatic, mild and hospitalized patients at day 1 of inclusion.**

**A**, PCA plot of the samples from different patient groups at D1 of inclusion based on 17-parameter whole-blood-count analysis. The percentage of explained variance is shown for each axis. ASP, asymptomatic patients, n=12; HP, hospitalized patients, n=15; MP, mild patients, n=53; D1, day 1.
**B, E, G**, the percentages of Lymphocytes (LYM, **B**), monocytes (Mon, **E**) and granulocytes (GRA, **G**) among white blood cells.
**C**, CRP (C-reactive protein) levels in different patient groups at D1 of inclusion.
**D, F, H, I, K**, Number of white blood cells (WBC, **D**), monocytes (Mon, **F**), granulocytes (GRA, **H**), red blood cells (RBC, **I**) and platelets (PLT, **K**) per µl ($mm^3$).
**J**, the hematocrit (HCT) levels (%) from different patients groups at D1 of inclusion.
**L, M**, Percentages of LYM in white blood cells (**L**) or CRP levels (**M**) from all ASP, all MP and four selected HP. To ensure the comparability with MP, we only selected four HP with at most 7-day delay from the onset of first symptoms to the inclusion day in these two panels. The data in **L** and **M** are identical to that in **B** and **C** except for HP.
Data represent individual values from all biological replicates; Mean ± standard deviation (S.D.); P-value was determined by the Kruskal-Wallis (non-parametric) test and corrected using the Dunn's multiple comparisons test. ns, not significant, *p<=0.05, **p<=0.01 and ***p<=0.001. Gray shading indicates the reported normal range for those different laboratory parameters. Related to Figure 1.

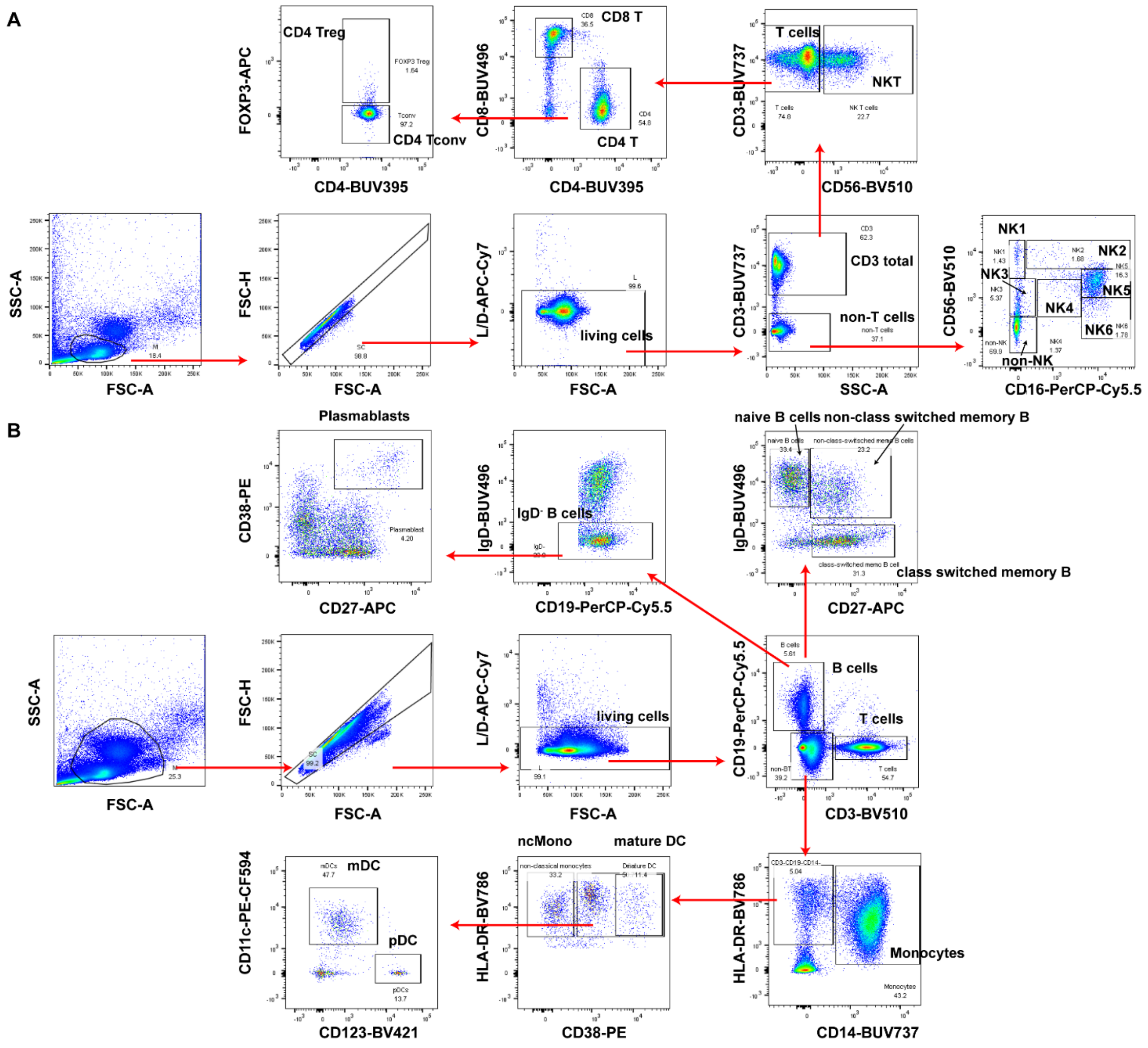


**Figure S2. General gating strategy to identify different immune subsets analysed in our study.**

**A**, Lymphocyte gating strategy (not including B cells). Treg, FOXP3$^+$CD4 regulatory T cells; Tconv, FOXP3$^-$CD4 conventional T cells; NK, natural killer cells; NKT, natural killer T cells.

**B,** Gating strategy to identify B cells, (non-) class-switched memory B cells, naïve B cells, T cells, subsets of monocytes, DC, mDC, pDC, mature DC and plasmablasts. ncMono, non-classical monocytes; DC, dendritic cells; mDC, myeloid DC; pDC, plasmacytoid DC.

Due to limited space, the functional markers or their combinations among each subset were not displayed here. It is also worthy to note that we used three different panels to perform a deep immunophenotyping analysis by flow cytometry. For some markers, different fluorochromes were used in different panels. Related to Figures 2 and 3.

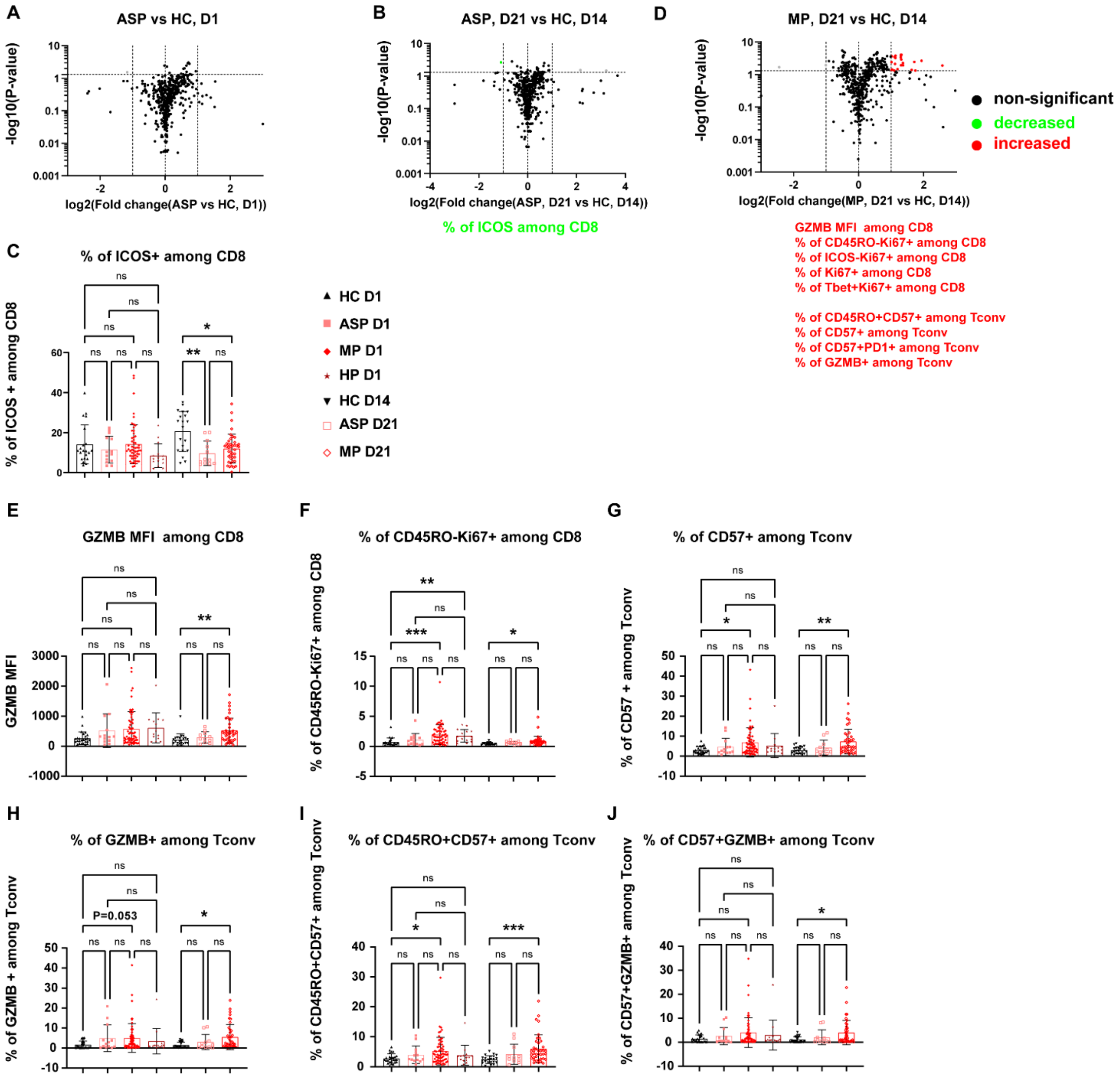


## Figure S3. Extended comparisons of immune subset responses between various groups.

**A**, **B**, Volcano plots showing the immune responses in ASP vs HC at D1 (baseline) (**A**) or ASP at D21 vs HC at D14 (**B**) following inclusion. Significant increased or decreased subsets (p<=0.05 and change fold >=2) were marked in red or green, respectively. The gray dot represents the subset not showing a significant change after applying the Dunn's multiple comparison test.

**C**, Frequency of ICOS$^{+}$ cells among CD8 T cells of different participant groups.

**D**, Volcano plot showing responses of different immune subsets in MP at D21 following inclusion relative to HC at D14 following inclusion. Significantly increased or decreased subsets (p<=0.05 and change fold >=2) were marked in red or green, respectively. The gray dot represents the subset not showing a significant change after applying the Dunn's multiple comparison test.

**E**, GZMB MFI (Geometric mean) among CD8 T cells from different groups.

**F**, Frequency of CD45RO$^{-}$Ki67$^{+}$ cells among CD8 T cells from different groups.

**G, H, I, J**, Frequency of CD57$^{+}$ cells (**G**), GZMB$^{+}$ cells (**H**), CD45RO$^{+}$CD57$^{+}$ cells (**I**) or CD57$^{+}$GZMB$^{+}$ cells (**J**) among CD4 Tconv cells (FOXP3$^{-}$CD4 T conventional cells) from different groups.

Data represent individual values from all biological replicates; Mean± standard deviation (S.D.); P-value was determined by the Kruskal-Wallis (nonparametric) test and corrected using the Dunn's multiple comparisons test. ASP,

asymptomatic patients, n=14; HC, household controls, n=26; HP, hospitalized patients, n=15; MP, mild patients, n=63; D1/D14/D21, day 1/day 14/day 21. ns, not significant, *p<=0.05, **p<=0.01 and ***p<=0.001. Related to Figure 2.

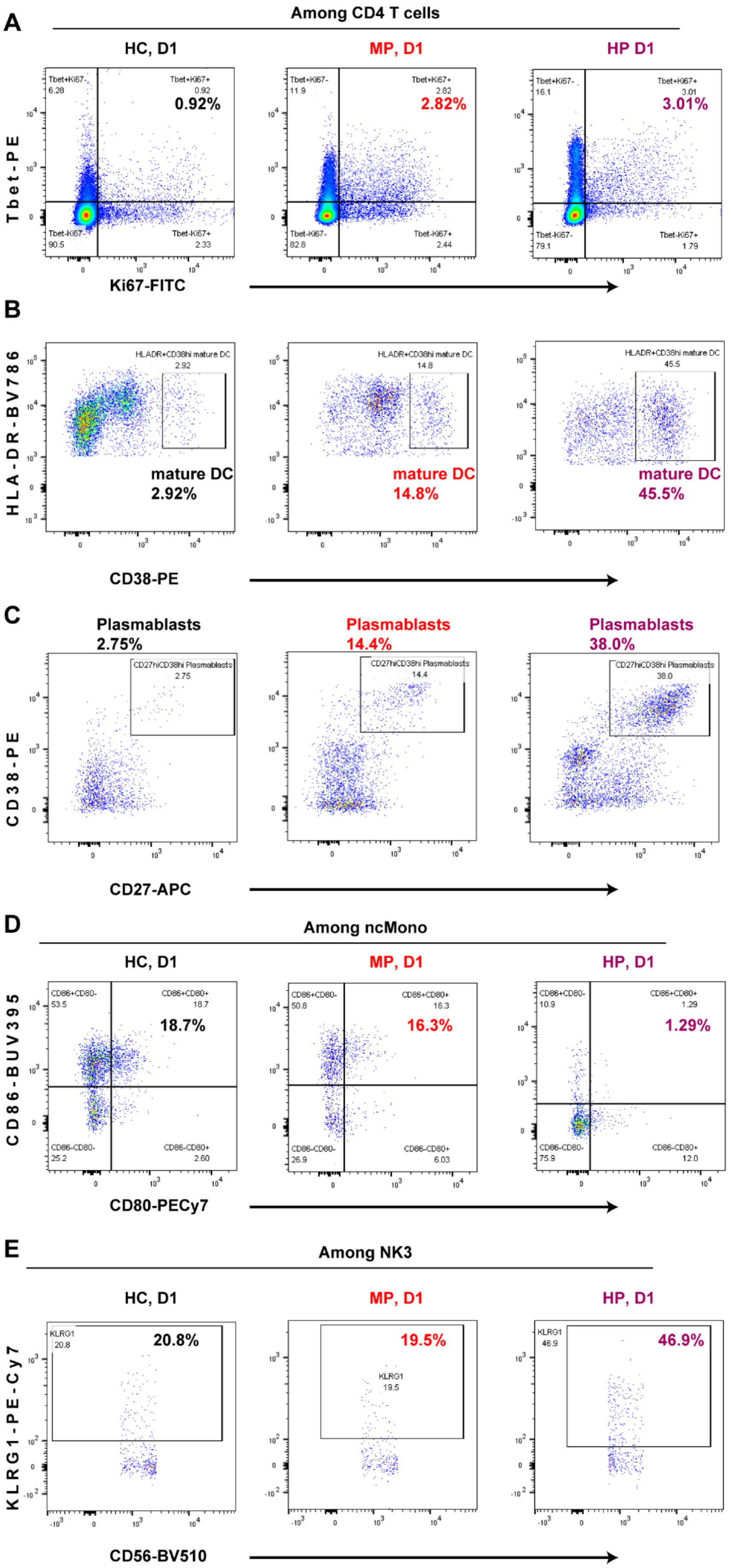

A
Among CD4 T cells
HC, D1
MP, D1
HP D1
Tbet-PE
Ki67-FITC
0.92%
2.82%
3.01%
B
HLA-DR-BV786
CD38-PE
mature DC
2.92%
14.8%
45.5%
C
Plasmablasts
2.75%
14.4%
38.0%
CD38-PE
CD27-APC
D
Among ncMono
CD86-BUV395
CD80-PECy7
18.7%
16.3%
1.29%
E
Among NK3
KLRG1-PE-Cy7
CD56-BV510
20.8%
19.5%
46.9%

**Figure S4. Extended representative flow cytometry plots in comparison of different groups at day 1 of inclusion.**

**A**, Representative flow-cytometry plots of the expression of Ki67 and Tbet among CD4 T cells from different participant groups. HC, household controls; HP, hospitalized patients; MP, mild patients; D1, day 1.
**B, C**, Representative flow-cytometry plots of the expression of CD38 and HLA-DR showing mature DC (**B**) or the expression of CD38 and CD27 showing plasmablasts (**C**) from different participant groups.
**D**, Representative flow-cytometry plots of the expression CD86 and CD80 among non-classical monocytes (ncMono) from different groups.
**E**, Representative flow-cytometry plots of the expression KLRG1 and CD56 among NK3 from different groups.
Related to Figures 2 and 3.

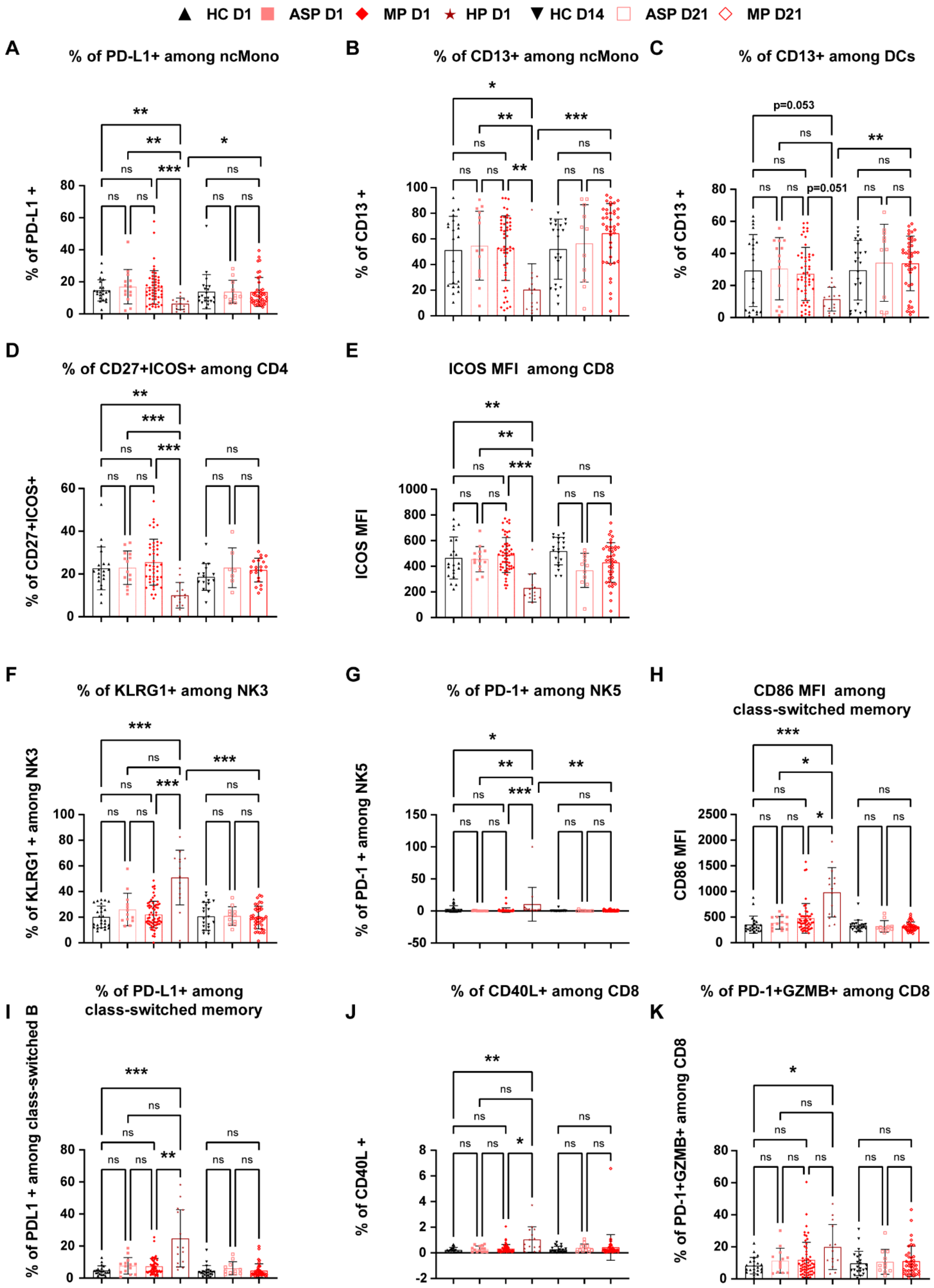

HC D1
ASP D1
MP D1
HP D1
HC D14
ASP D21
MP D21
A
% of PD-L1+ among ncMono
% of PD-L1 +
B
% of CD13+ among ncMono
% of CD13 +
C
% of CD13+ among DCs
% of CD13 +
p=0.053
p=0.051
D
% of CD27+ICOS+ among CD4
% of CD27+ICOS+
E
ICOS MFI among CD8
ICOS MFI
F
% of KLRG1+ among NK3
% of KLRG1 + among NK3
G
% of PD-1+ among NK5
% of PD-1 + among NK5
H
CD86 MFI among class-switched memory
CD86 MFI
I
% of PD-L1+ among class-switched memory
% of PDL1 + among class-switched B
J
% of CD40L+ among CD8
% of CD40L +
K
% of PD-1+GZMB+ among CD8
% of PD-1+GZMB+ among CD8

**Figure S5. Extended analysis of early-stage immune features characterizing mild patents from hospitalized ones.**

**A**, **B**, Frequency of cells expressing PD-L1 (**A**) or CD13 (**B**) among ncMono (non-classical monocytes) from different participant groups.
**C**, Frequency of $CD13^+$ cells among HLA-$DR^+CD38^+$ DCs (including $CD38^{high}$ cells, refer to Fig. S2).
**D, E,** Frequency of $CD27^+ICOS^+$ cells among CD4 cells (**D**) or ICOS MFI (Geometric mean) (**E**) among CD8 T cells.
**F, G**, Frequency of $KLRG1^+$ cells among NK3 (**F**) or frequency of PD-$1^+$ cells among NK5 (**G**).
**H, I**, CD86 MFI (**H**) or the frequency of PD-$L1^+$ cells (**I**) among class-switched memory B cells.
**J, K**, Frequency of $CD40L^+$ cells (**J**) or frequency of PD-$1^+GZMB^+$ cells (**K**) among CD8 T cells.
Data represent individual values from all biological replicates; Mean± standard deviation (S.D.); P-value was determined by the Kruskal-Wallis (nonparametric) test and corrected using the Dunn's multiple comparisons test. ASP, asymptomatic patients, n=14; HC, household controls, n=26; HP, hospitalized patients, n=15; MP, mild patients, n=63; D1/D14/D21, day 1/day 14/day 21. ns, not significant, *p<=0.05, **p<=0.01 and ***p<=0.001. Related to Figure 3.

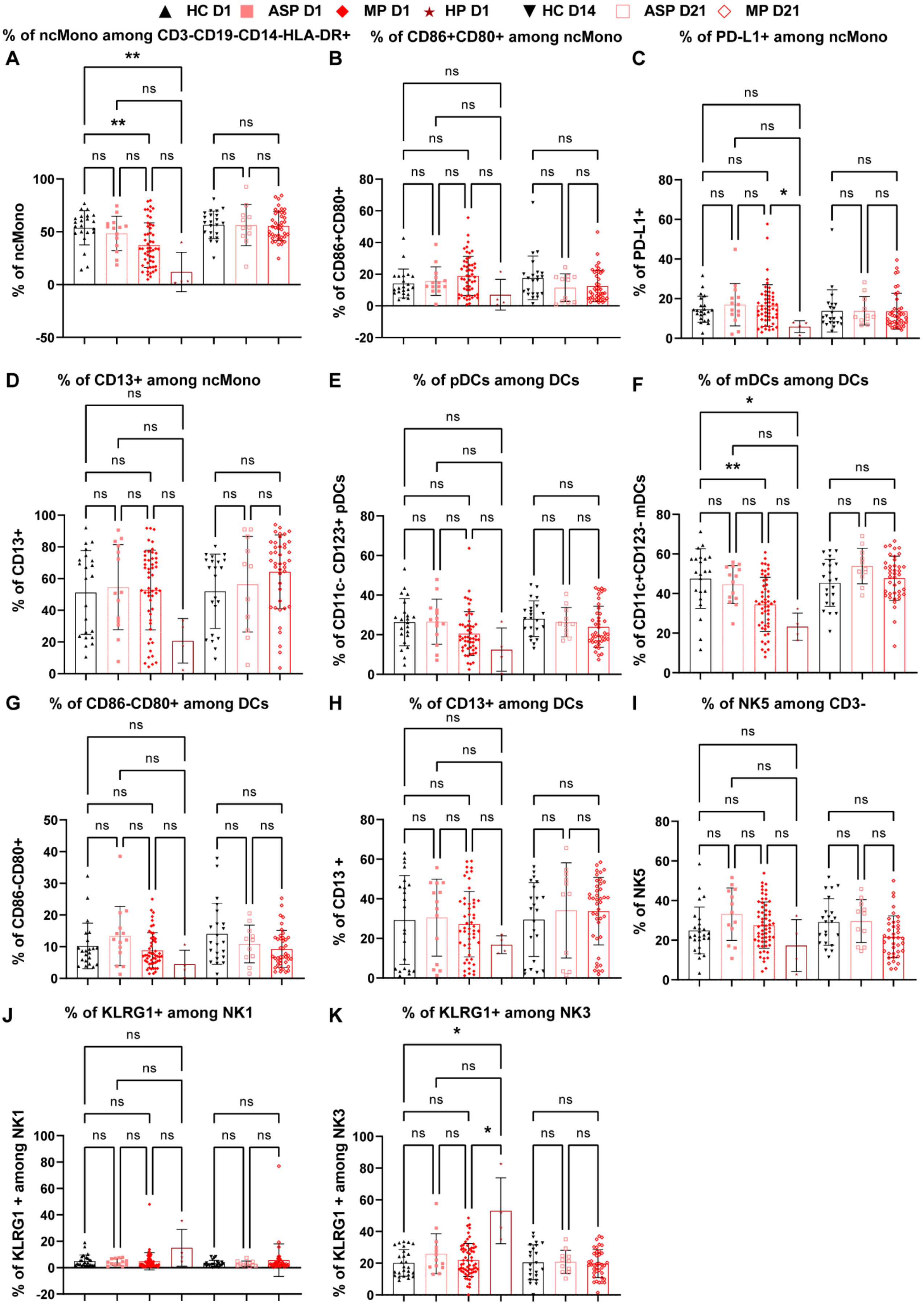
HC D1
ASP D1
MP D1
HP D1
HC D14
ASP D21
MP D21
A
% of ncMono among CD3-CD19-CD14-HLA-DR+
% of ncMono
B
% of CD86+CD80+ among ncMono
% of CD86+CD80+
C
% of PD-L1+ among ncMono
% of PD-L1+
D
% of CD13+ among ncMono
% of CD13+
E
% of pDCs among DCs
% of CD11c- CD123+ pDCs
F
% of mDCs among DCs
% of CD11c+CD123- mDCs
G
% of CD86-CD80+ among DCs
% of CD86-CD80+
H
% of CD13+ among DCs
% of CD13 +
I
% of NK5 among CD3-
% of NK5
J
% of KLRG1+ among NK1
% of KLRG1 + among NK1
K
% of KLRG1+ among NK3
% of KLRG1 + among NK3

**Figure S6. Sub-cohort analysis on impaired early-stage responses of non-classical monocytes, DC and NK cells in four hospitalized COVID-19 patients with a shorter prodromal phase.**

**A, B, C, D**, Percentage of ncMono (non-classical monocytes) among CD3-CD19-CD14-HLA-DR+ cells (**A**), percentages of CD86+CD80+ population among ncMono (**B**), percentage of PD-L1+ subset (**C**) or CD13+ population (**D**) among ncMono in the selected four HP at D1 in comparison with other groups. To ensure the comparability in terms of infection stage between different severity groups, we only selected four HP with at most 7-day delay from the onset of first symptoms to the inclusion day in this Supplementary Figure.
**E, F**, Percentage of pDCs (**E**) or mDCs (**F**) among total HLA-DR+CD38+ DCs (for gating strategy, refer to Fig. S2)
**G**, **H**, Percentage of CD86-CD80+ subset (**G**) or CD13+ cells (**H**) among total HLA-DR+CD38+ DCs.
**I**, Percentage of NK5 among CD3- cells
**J, K**, Percentage of KLRG1+ subset among NK1 cells (**J**) or among NK3 cells (**K**).
Of note, the values of this figure might be identical to that of the related figures within all the groups except for HP at D1. As argued above and in the text, this figure is not resulted by the duplicated usage of the same data, but to show a different analysis strategy. Data represent individual values from all biological replicates; Mean± standard deviation (S.D.); P-value was determined by the Kruskal-Wallis (nonparametric) test and corrected using the Dunn’s multiple comparisons test. ASP, asymptomatic patients, n=14; HC, household controls, n=26; HP, hospitalized patients, n=4; MP, mild patients, n=63; D1/D14/D21, day 1/day 14/day 21. ns, not significant, *p<=0.05, **p<=0.01 and ***p<=0.001. Related to Figure 3.

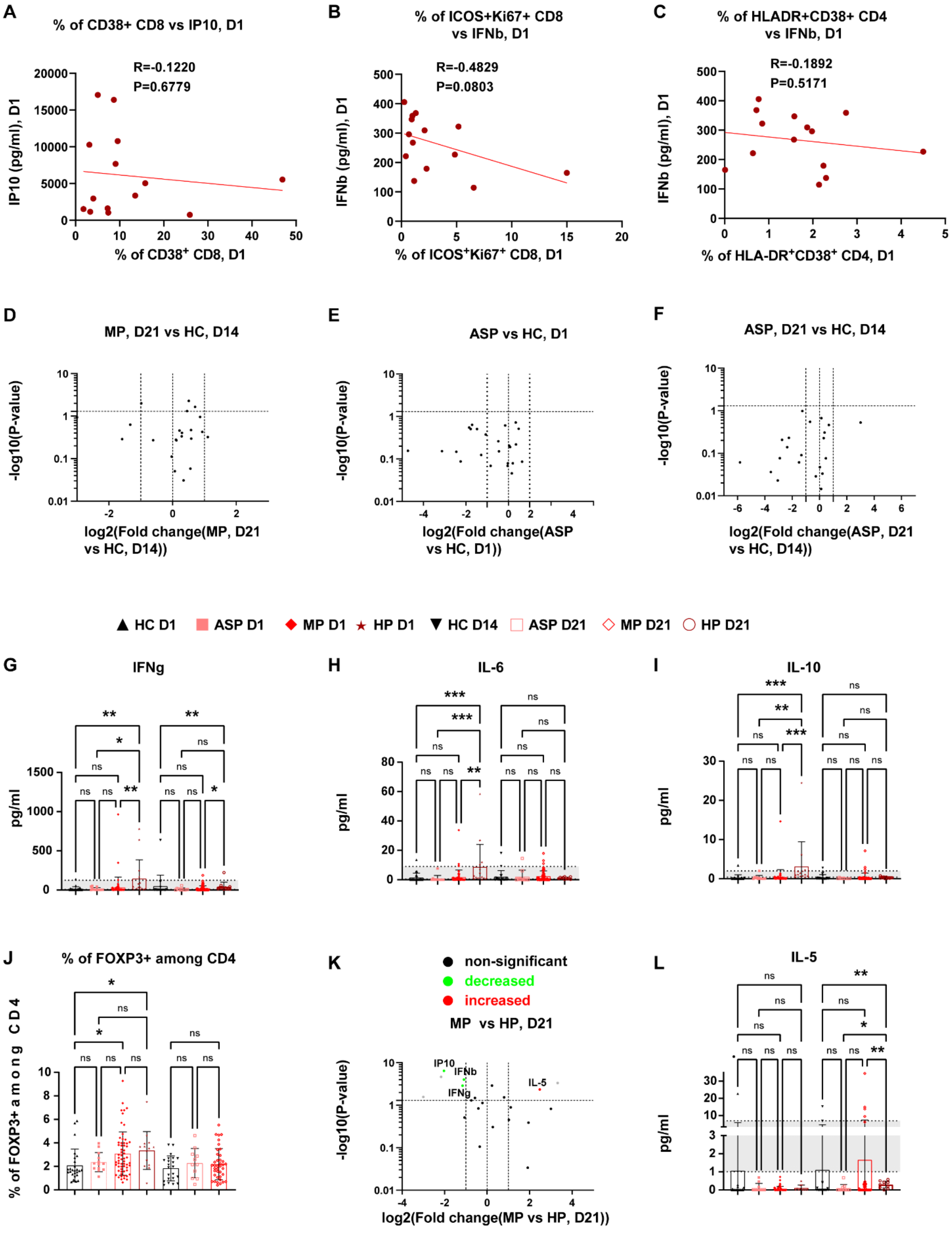
A
% of CD38+ CD8 vs IP10, D1
R=-0.1220
P=0.6779
IP10 (pg/ml), D1
% of CD38+ CD8, D1
B
% of ICOS+Ki67+ CD8 vs IFNb, D1
R=-0.4829
P=0.0803
IFNb (pg/ml), D1
% of ICOS+Ki67+ CD8, D1
C
% of HLADR+CD38+ CD4 vs IFNb, D1
R=-0.1892
P=0.5171
IFNb (pg/ml), D1
% of HLA-DR+CD38+ CD4, D1
D
MP, D21 vs HC, D14
-log10(P-value)
log2(Fold change(MP, D21 vs HC, D14))
E
ASP vs HC, D1
-log10(P-value)
log2(Fold change(ASP vs HC, D1))
F
ASP, D21 vs HC, D14
-log10(P-value)
log2(Fold change(ASP, D21 vs HC, D14))
HC D1
ASP D1
MP D1
HP D1
HC D14
ASP D21
MP D21
HP D21
G
IFNg
pg/ml
H
IL-6
pg/ml
I
IL-10
pg/ml
J
% of FOXP3+ among CD4
% of FOXP3+ among CD4
K
non-significant
decreased
increased
MP vs HP, D21
IP10
IFNb
IFNg
IL-5
-log10(P-value)
log2(Fold change(MP vs HP, D21))
L
IL-5
pg/ml

**Figure S7. Extended comparison of serological cytokine/chemokine responses between different participant groups at day 1 or day 21 following inclusion.**

**A, B**, Correlation between the frequency of CD38+ cells among total CD8 T cells and circulating IP10 levels (**A**), or between the frequency of ICOS+Ki67+ among total CD8 T cells and circulating IFN-β levels (**B**) in HP at D1 of inclusion. R, Pearson correlation coefficient. Different groups were marked by different indicated colours.
**C**, Correlation between the frequency of HLA-DR+CD38+ cells among total CD4 T cells and circulating IFN-β levels in HP at D1. Pearson correlation coefficient.
**D**, Volcano plot showing 24 cytokine/chemokine responses of MP at D21 versus HC at D14 of inclusion.
**E**, Volcano plot showing 24 cytokine/chemokine responses of ASP versus HC at D1 following inclusion.
**F**, Volcano plot showing 24 cytokine/chemokine responses of ASP at D21 versus HC at D14 of inclusion. The gray dot represents the analytes showing a significant change but displaying values lower than the reported normal physiological levels in different groups, even in HC.
**G, H, I, L,** Scatter dot plots of IFN-γ (**G**), IL-6 (**H**), IL-10 (**I**) and IL-5 (**L**) of different participant groups.
**J**, Frequency of $FOXP3^{+}$ Treg cells among total CD4 T cells.
**K**, Volcano plot showing 24 cytokine/chemokine responses of MP versus HP at D21 following inclusion.
Data represent individual values from all biological replicates; Mean± standard deviation (S.D.); P-value was determined by the Kruskal-Wallis (nonparametric) test and corrected using the Dunn’s multiple comparisons test. ASP, asymptomatic patients, n=14; HC, household controls, n=26; HP, hospitalized patients, n=15; MP, mild patients, n=63; D1/D14/D21, day 1/day 14/day 21. ns , not significant, *p<=0.05, **p<=0.01 and ***p<=0.001. Gray shading indicates the reported normal range for different cytokines. Related to Figure 4.

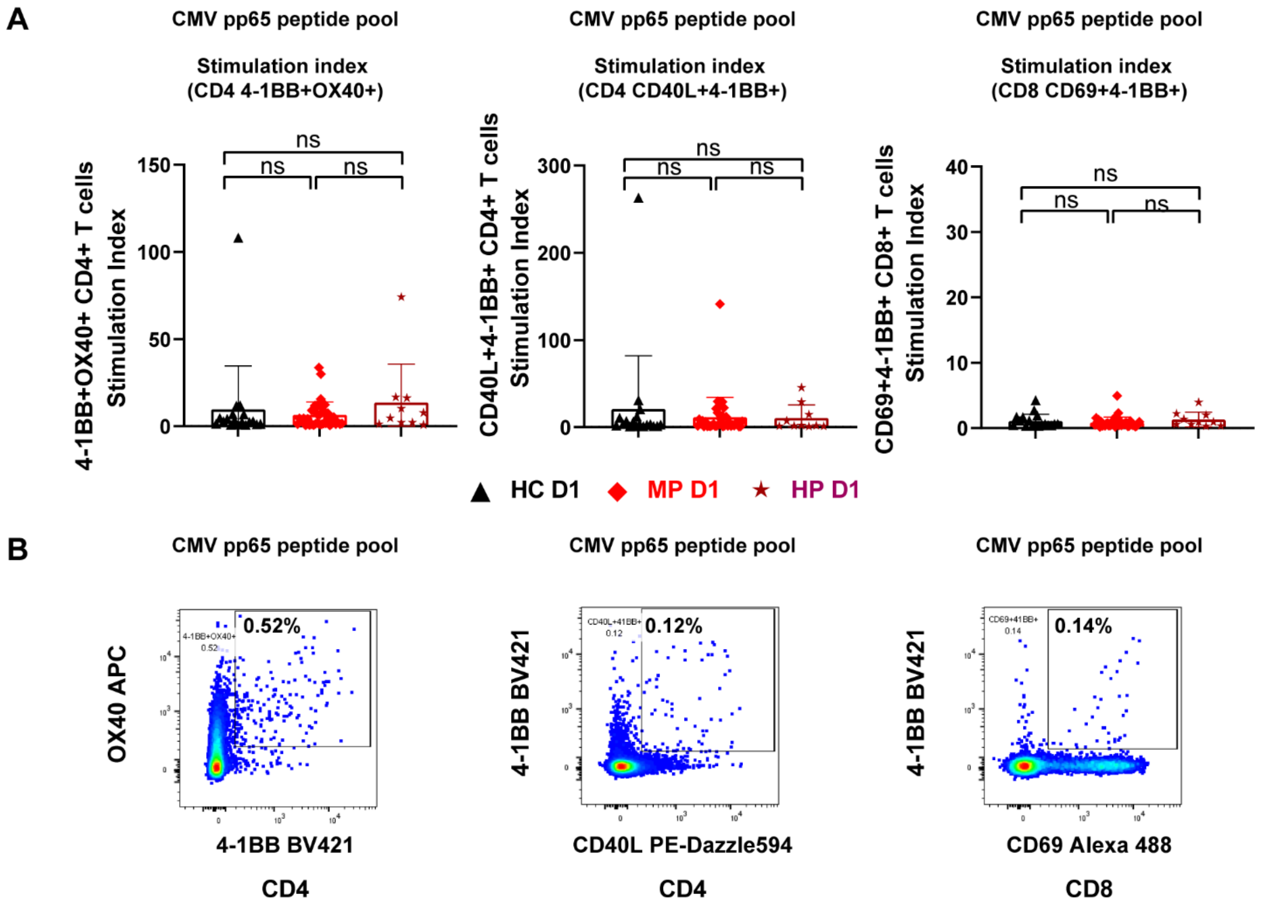


**Figure S8. No difference in the AIM response to the restimulation of the CMV peptide pool between household controls, mild and hospitalized patients at day 1.**

**A**, Normalized CMV-specific response (stimulation index, SI) among total CD4 T cells quantified by 4-1BB/CD137 and OX40/CD134 double positive cells (left panel), among total CD4 T cells quantified by CD40L and 4-1BB double positive cells (middle panel), or among total CD8 T cells quantified by CD69 and 4-1BB double positive cells (right panel) following stimulation with the CMV pp65 peptide pool or negative control in HC, MP or HP at D1. The normalized value (SI) was calculated using the original percentage in the presence of the CMV peptide pool divided by the percentage in negative control (H20) of the same sample (also refer to **STAR Methods**).

**B**, Representative flow-cytometry plots of 4-1BB/CD137 and OX40/CD134 among CD4 T cells (left panel), of CD40L and 4-1BB among CD4 (middle panel), or of CD69 and 4-1BB among CD8 T cells (right panel).

Data represent individual values from all biological replicates. Mean± standard deviation (S.D.). P-value from **A** was determined by non-paired two-tailed Student *t* test. HC, household controls, n=18; HP, hospitalized patients, n=10; MP, mild patients, n=40; D1, day 1. ns, not significant, *p<=0.05, **p<=0.01 and ***p<=0.001. Related to **STAR Methods** and Figure 6.

## Supplementary Tables

**Table S1. Demographics of our analysed longitudinal COVID-19 cohort.**

| Clinical characteristics of the cohort | All patients, D1 | Asymptomatic, D1 | Mild, D1 | Hospitalized, D1 | Household controls, D1 | All patients, D21 | Asymptomatic, D21 | Mild, D21 | Household Controls, D14 |
|---|---|---|---|---|---|---|---|---|---|
| **median (IQR)** | | | | | | | | | |
| **Age (years)** | 44 (30-53) | 44 (24.5-55.5) | 38 (28.5-48) | 57 (49-61) | 33.5 (26-40.75) | 40.5 (30.8-48.5) | 44 (29-53) | 40 (31-48) | 33.5 (27-42.25) |
| **BMI** | 25.7 (23.5-28.6) | 24.8 (22.9-26.6) | 25.8 (23.5-29.4) | 26.64 (24.9-29.1) | | 25.9 (23.9-27.9) | 24.8 (22.3-26.1) | 26.3 (24.4-28.7) | |
| **concerned n (percentage among the given category)** | | | | | | | | | |
| **Male (%)** | 54 (63.5%) | 9 (69.2%) | 39 (63.9%) | 8 (57.1%) | 7 (30.4%) | 33 (66%) | 8 (72.7%) | 25 (64.1%) | 7 (35%) |
| **Smoking (%)** | 14 (18.7%) | 3 (23.1%) | 11 (18.1%) | | | 9 (18%) | 2 (18.2%) | 7 (18%) | |
| **Former smoker (%)** | 16 (21.4%) | 2 (15.4%) | 14 (23%) | | | 12 (24%) | 2 (18.2%) | 10 (25.7%) | |
| **Whole Blood Count parameters: median (IQR)** | | | | | | | | | |
| **WBC, white blood cell count (10E3/uL)** | 5.25 (3.9-7.625) | 5.3 (4.4-6.1) | 4.6 (3.5-5.7) | 9.6 (8.85-11.05) | | | | | |
| **#Lymphocyte, LYM (10E3/uL)** | 1.65 (1.2-2.1) | 1.8 (1.4-2.1) | 1.6 (1.2-2.1) | 1.6 (1-2.1) | | | | | |
| **%Lymphocyte, LYM (%)** | 34.05 (25.575-40.75) | 33 (29.8-37.7) | 36.9 (31-43.9) | 16.8 (10.95-21.3) | | | | | |
| **#Monocyte, MON (10E3/uL)** | 0.3 (0.2-0.6) | 0.3 (0.28-0.43) | 0.2 (0.2-0.4) | 1.2 (0.7-2) | | | | | |
| **%Monocyte, MON (%)** | 7.3 (6.4-9.65) | 7.15 (6.5-7.9) | 6.9 (5.8-8.5) | 12.8 (9.7-17.1) | | | | | |
| **#Granulocyte, GRA (10E3/uL)** | 3.05 (2.175-4.6) | 3.35 (2.5-3.8) | 2.6 (1.8-3.8) | 7.2 (6.25-7.55) | | | | | |
| **%Granulocyte, GRA (%)** | 58.1 (50.9-65.2) | 59.7 (52.1-63) | 54.7 (48.7-62.7) | 67.8 (64.15-77.05) | | | | | |
| **Red Blood Cell, RBC (10E6/uL)** | 5.265 (4.865-5.7125) | 5.41 (5.0-5.7) | 5.31 (5.1-5.9) | 4.73 (4.335-4.945) | | | | | |

| | | | | | | | | | |
|---|---|---|---|---|---|---|---|---|---|
| **Hemoglobin, HGB (g/dl)** | 15.5 (14.1-17.025) | 15.6 (15.1-17.4) | 15.8 (14.7-17) | 13.9 (13.05-14.85) | | | | | |
| **Hematocrit, HCT (%)** | 48.25 (44.05-52.65) | 49.1 (46.9-54.4) | 48.8 (45.6-52.8) | 42.9 (39.5-45.7) | | | | | |
| **Platelet, PLT (10E3/uL)** | 175 (144-212) | 179.5 (154.8-209.3) | 167 (130-199) | 235 (163-286.5) | | | | | |
| **Mean Cell Volume, MCV (um3)** | 92 (89-94) | 92.5 (89.8-95) | 92 (89-94) | 91 (90-93) | | | | | |
| **Mean Cell Hemoglobin, MCH (pg)** | 29.35 (28.5-30.85) | 29.35 (28.5-30.3) | 28.9 (28.5-30.7) | 30.4 (29.9-31) | | | | | |
| **Mean Cell Hemoglobin Concentration, MCHC (g/dL)** | 32.1 (31.6-32.6) | 31.9 (31.8-32.4) | 32 (31.4-32.4) | 33.3 (33-33.5) | | | | | |
| **Red Cell Distribution Width, RDW (%)** | 14.35 (14-14.7) | 14.45 (14.1-14.6) | 14.4 (14.1-14.8) | 14.1 (13.95-14.55) | | | | | |
| **Mean Platelet Volume, MPV (um3)** | 8.4 (8-8.8) | 8.35 (8.1-9.0) | 8.4 (8-8.7) | 8.3 (8-8.6) | | | | | |
| **C-reactive protein, CRP (mg/L)** | 0.65 (0-10.075) | 0 (0-0.03) | 0.4 (0-1.6) | 38 (18.4-58) | | | | | |
| **Comorbidity: concerned n (the percentage among the given category)[§]** | | | | | | | | | |
| **Hypertension** | 8(10.7%) | 2(15.4%) | 6(9.9%) | | | 6(12%) | 1(9.1%) | 5 (12.9%) | NA* |
| **Chronic heart disease, including congenital heart disease (except hypertension)** | 5(6.7%) | 1(7.7%) | 4(6.6%) | | | 2 (4%) | 1(9.1%) | 1(2.6%) | NA |
| **Chronic lung disease (except asthma)** | 0 (0%) | 0 (0%) | 0 (0%) | | | 0 (0%) | 0 (0%) | 0 (0%) | NA |
| **Asthma (clinical diagnosis made)** | 3 (4%) | 0 (0%) | 3 (5%) | | | 0 (0%) | 0 (0%) | 0 (0%) | NA |
| **Chronic kidney disease** | 0 (0%) | 0 (0%) | 0 (0%) | | | 0 (0%) | 0 (0%) | 0 (0%) | NA |
| **Renal failure requiring dialysis** | 0 (0%) | 0 (0%) | 0 (0%) | | | 0 (0%) | 0 (0%) | 0 (0%) | NA |
| **Moderate or severe liver disease** | 1(1.4%) | 0 (0%) | 1 (1.7%) | | | 1 (2%) | 0 (0%) | 1(2.6%) | NA |
| **Mild liver disease** | 1(1.4%) | 1 (7.7%) | 0 (0%) | | | 1 (2%) | 1 (9.1%) | 0 (0%) | NA |
| **Chronic neurological disorder** | 1(1.4%) | 0 (0%) | 1 (1.7%) | | | 0 (0%) | 0 (0%) | 0 (0%) | NA |

| | | | | | | | | | |
|---|---|---|---|---|---|---|---|---|---|
| **Cancer** | 0 (0%) | 0 (0%) | 0 (0%) | | | 0 (0%) | 0 (0%) | 0 (0%) | NA |
| **Chronic hematologic disease** | 3 (4%) | 0 (0%) | 3 (5%) | | | 1 (2%) | 0 (0%) | 1(2.6%) | NA |
| **HIV / AIDS** | 0 (0%) | 0 (0%) | 0 (0%) | | | 0 (0%) | 0 (0%) | 0 (0%) | NA |
| **Obesity (clinical diagnosis made)** | 4(5.4%) | 0 (0%) | 4 (6.6%) | | | 4 (8%) | 0 (0%) | 4(10.3%) | NA |
| **Diabetes with associated complications** | 0 (0%) | 0 (0%) | 0 (0%) | | | 0 (0%) | 0 (0%) | 0 (0%) | NA |
| **Uncomplicated diabetes** | 5 (6.7%) | 0 (0%) | 5 (8.2%) | | | 2 (4%) | 0 (0%) | 2(5.2%) | NA |
| **Rheumatologic disease** | 1 (1.4%) | 0 (0%) | 1 (1.7%) | | | 1 (2%) | 0 (0%) | 1(2.6%) | NA |
| **Malnutrition** | 1(1.4%) | 0 (0%) | 1 (1.7%) | | | 1 (2%) | 0 (0%) | 1(2.6%) | NA |
| **Chronic obstructive pulmonary disease (COPD)** | 1(1.4%) | 0 (0%) | 1 (1.7%) | | | 0 (0%) | 0 (0%) | 0 (0%) | NA |
| **Other notable diseases or risk factors** | 12(16%) | 1 (7.7%) | 11(18.1%) | | | 8 (16%) | 0 (0%) | 8(20.6%) | NA |

§, the total number of patients here only refer to asymptomatic and mild patients, but not hospitalized patients.
*, NA or empty, no information available.
IQR: 25% percentile-75% percentile.
n, the number of participants analyzed in the given category.
Related to Figure 1 and **STAR Methods**.
